# Immunogenicity of glycans on biotherapeutic drugs produced in plant expression systems—The taliglucerase alfa story

**DOI:** 10.1371/journal.pone.0186211

**Published:** 2017-10-31

**Authors:** Bonita Rup, Sari Alon, Bat-Chen Amit-Cohen, Einat Brill Almon, Raul Chertkoff, Yoram Tekoah, Pauline M. Rudd

**Affiliations:** 1 Bonnie Rup Consulting, LLC, Reading, Massachusetts, United States of America; 2 Product Development, Protalix LTD, Carmiel, Israel; 3 Research and Development, Protalix LTD, Carmiel, Israel; 4 National Institute for Bioprocessing Research and Training, Dublin, Ireland; 5 Bioprocessing Technology Institute, AStar, Singapore; Baylor Health Care System, UNITED STATES

## Abstract

Plants are a promising alternative for the production of biotherapeutics. Manufacturing *in-planta* adds plant specific glycans. To understand immunogenic potential of these glycans, we developed a validated method to detect plant specific glycan antibodies in human serum. Using this assay, low prevalence of pre-existing anti-plant glycan antibodies was found in healthy humans (13.5%) and in glucocerebrosidase-deficient Gaucher disease (GD) patients (5%). A low incidence (9% in naïve patient and none in treatment experienced patients) of induced anti-plant glycan antibodies was observed in GD patients after up to 30 months replacement therapy treatment with taliglucerase alfa, a version of human glucocerebrosidase produced in plant cells. Detailed evaluation of clinical safety and efficacy endpoints indicated that anti-plant glycan antibodies did not affect the safety or efficacy of taliglucerase alfa in patients. This study shows the benefit of using large scale human trials to evaluate the immunogenicity risk of plant derived glycans, and indicates no apparent risk related to anti-plant glycan antibodies.

## Introduction

Genetically engineered plants or plant cells can now be used to express plant-derived pharmaceutical proteins (biopharmaceuticals) or vaccines on an industrial scale [1, 2]. The advantages of the plant cell expression systems include large-scale production capacity, lack of animal pathogen contamination and low cost of biomass production compared to mammalian systems [3], in addition to the production in a GMP environment [4].

Although plants, in common with other eukaryotic organisms, produce glycoproteins with N-glycans attached to asparagine residues, these glycans differ in structure from those of mammals [5]. Plant glycans may contain an α(1,3)-fucose linked to the proximal N-acetylglucosamine (GlcNAc) residue and/or a β(1,2)-xylose residue attached to the bisecting mannose of the glycan core, which are not present in mammalian proteins [3].

The immunogenic effect of the plant glycan moieties has been the basis of much debate in the literature. Following identification of IgE antibodies in allergic patients that cross react with these structures on glycoproteins from a variety of species, the β(1,2) xylose and α(1,3) fucose structures have been designated as cross-reactive carbohydrate determinants [6]. However, further studies have indicated that anti-cross-reactive carbohydrate determinant antibodies do not bind equally to all glycans with α(1,3) fucose and/or β(1,2) xylose, indicating that other features of the glycan structure or protein play a critical role in the binding [7]. Many studies have evaluated the potential relevance of plant-derived carbohydrate epitopes for the diagnoses and treatment of allergic diseases [8–12], but no correlation between the presence of carbohydrate-specific IgEs and clinical effects has been demonstrated.

Concerns have been raised that these unique glyco-epitopes could elicit unwanted immunogenic responses when plant derived biopharmaceuticals and vaccines are administered to humans [13–15]. Thus, various animal models have been studied to elucidate the immunogenicity of plant-derived glycoproteins [16–24]. The observed immunogenicity of plant glyco-epitopes in some laboratory animals immunized with plant proteins carrying these epitopes raises questions about their potential immunogenicity risk in the context of human therapy [13, 25]. Although studies in humans have reported pre-existing antibodies to various non-human components of biotherapeutic products, such as antibodies to animal host cell-derived proteins [26], bovine serum albumen [27] or polyethylene glycol [28] in the general population, in the case of anti-plant glycan antibodies, the data are controversial [13, 29, 30]. To date, only a few clinical examples have involved the study of the natural prevalence of IgG antibodies against plant-glycans in the general human population. Bardor et al. [13] investigated the presence of total antibodies raised against plant-glycans in 53 nonallergic human blood donors and concluded that sera from about 50% of non-allergic blood donors contain antibodies specific for core β(1,2)-xylose, whereas 25% have antibodies against core α(1,3)-fucose. In another study, Landry et al. [29] reported that in a Phase I clinical trial for a plant-derived vaccine against Avian H5N1 Influenza, only 7/48 subjects (14.6%) had detectable levels of IgG recognizing plant N-glycans prior to vaccination. An extended study by Ward et al. [30] found that 19.2% of the subjects were positive for IgG antibodies to plant glyco-epitopes prior to vaccination. Additionally, 34% of the subjects developed transient IgG, and in some cases IgE, to plant glyco-epitopes after vaccination, but no subject mounted an IgE response to the xylose and fucose containing motifs and no subject developed allergic/hypersensitivity response.

Taliglucerase alfa (TGA) (Protalix Biotherapeutics, Carmiel, Israel), an acid β-glucosidase Enzyme Replacement Therapy (ERT) for treatment of Gaucher Disease (GD), is the first plant cell-expressed biotherapeutic approved for use in humans [31]. The various β-glucocerebrosidase based drugs used for Gaucher ERT are produced in different cell types, resulting in differences in glycosylation, but in all cases, most of the glycans have terminal mannose residues and the differences in glycan structures do not seem to affect enzyme activity, substrate recognition, stability, uptake into macrophages, or uptake to organs [32]. Furthermore, currently available efficacy data derived from clinical trials suggest that there are no significant differences between TGA, produced in plant cell culture, and other β-glucocerebrosidase based products produced in mammalian cells in achieving clinical improvement in all main parameters of GD [33–35].

In the case of TGA, a tri-mannose glycoform with the addition of β(1,2) xylose and α(1,3) fucose is present at above 90% of the total glycan pool [32]. The plant glycan structures on TGA are likely to be surface exposed [36] and therefore may represent potentially unique epitopes for binding or induction of antibodies against the plant glycans. In a Phase I clinical trial in healthy human volunteers using TGA, no obvious adverse side effects that could be attributed to these N-glycan residues have been reported and no anti-drug antibodies (ADA) were detected [3]. Since therapeutic proteins can be associated with the development of ADA, an extended study in humans is necessary to fully evaluate the influence of the plant-derived glycans. Being the only recombinant plant-derived human therapeutic approved and on the market to-date, TGA is a good candidate to be used for such an extended analysis of plant glycan-specific immunogenicity.

In previously described studies of anti-plant glycan antibody immunogenicity [13, 29, 30], there was limited information on the assays used and on how criteria for distinguishing positive and negative samples were established. Immunogenicity evaluations and relevant characterization of detected ADA are necessary parts of biotherapeutic clinical safety and efficacy investigations. These evaluations should be conducted using validated assays to detect and characterize ADA and rigorous statistical methods for establishing cut-points for distinguishing positive and negative samples. For the TGA clinical immunogenicity evaluation, validated bioanalytical methods were developed and used to detect ADA to the TGA and to the plant glycan epitopes on TGA. The outcome of this study, which is the first large scale and long term report based on clinical data, is a substantial addition to the understanding of the capacity of plant derived glycans to induce unwanted immunogenic effects in patients.

## Materials and methods

### Materials

All materials were purchased from Sigma, unless otherwise indicated. Flat-bottomed Nunc MaxiSorp ELISA plates were purchased from Nalge Nunc International (Rochester, NY). TGA was provided by Protalix Ltd. Mouse monoclonal anti-TGA antibodies were prepared by Green Mountain Antibodies (Burlington, VT). Rabbit anti-horse radish peroxidase (HRP) antibodies Cat #AS09-549 and Cat #P7899 were purchased from Agrisera and Sigma, respectively, and used as a positive control. Monoclonal anti-TGA antibody produced in mice was a gift from Pfizer Inc. and was used as a negative control. HRP (Cat #P8150 and #P8250) was purchased from Sigma. Trypsin was acquired from Roche Diagnostics GmbH, Mannheim, Germany and peptide N-glycosidase A from Europa Ltd, Cambridge, UK. Pooled and individual normal human sera were obtained from Bioreclamation (Hicksville, NY). GD sera positive for anti-TGA antibodies were obtained from the clinical trials detailed below.

### Normal human serum samples

Individual normal human serum samples were acquired from Bioreclamation IVT (Westbury, NY). A total of 52 adult samples (26 male and 26 female) were analysed to determine the assay cutpoint, which was used to distinguish between positive and negative samples to anti-plant glycan antibodies.

### Clinical serum samples

**Fig 1:**
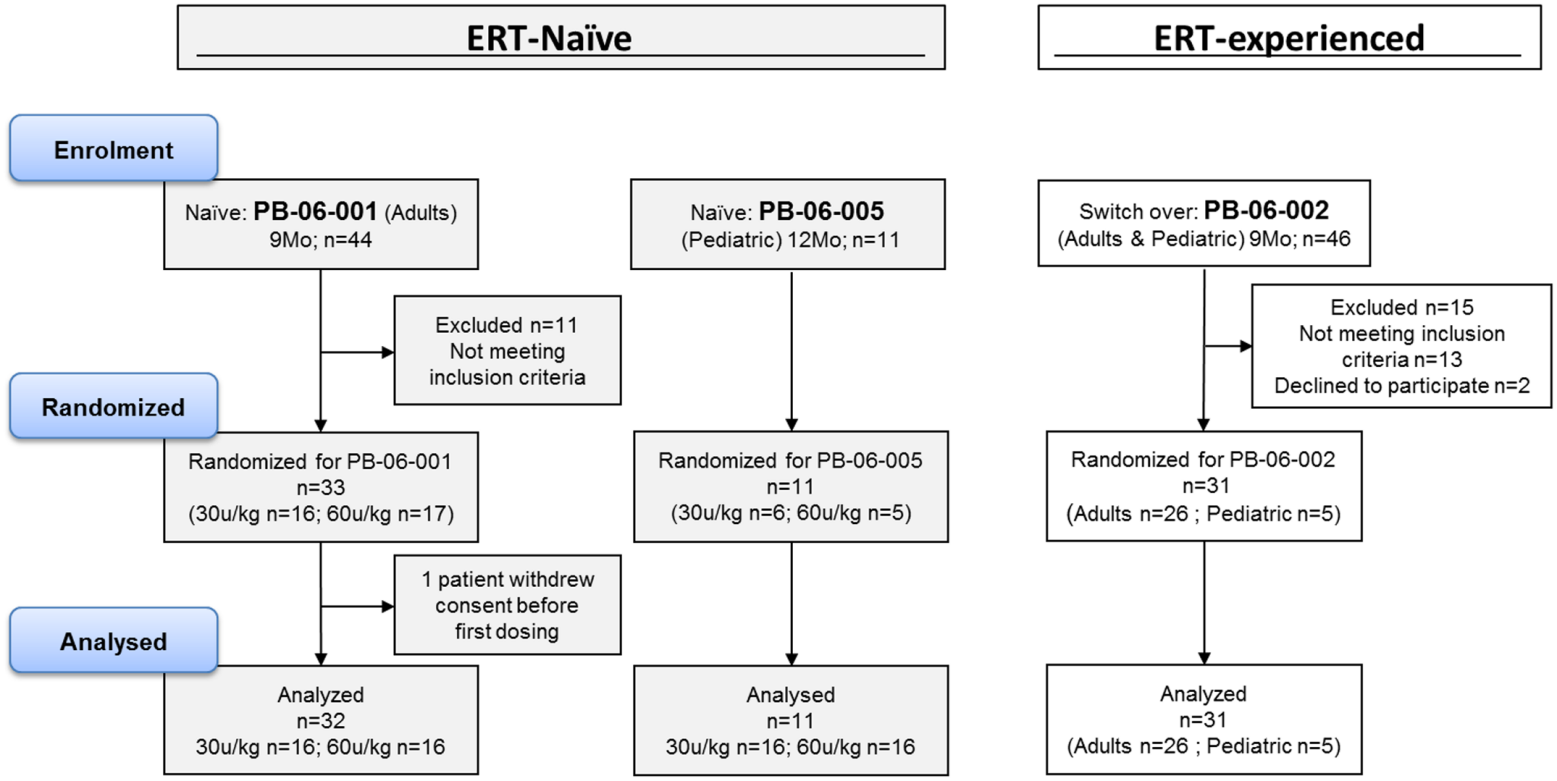
A Consort flowchart of the clinical studies included in the study. Consort flowchart shows all three studies with original enrolment, number of excluded subjects and the final amount of subjects included in the immunogenicity tests.

Patient serum samples were obtained, following written consent of adults and obtained consent from parents or guardians of the minors included in the various clinical studies indicated below. The consent forms were signed and dated by the subjects or parent/guardian before he/she was exposed to any protocol-specific procedure. Samples that were tested positive for ADA (and had sufficient remaining volume for additional characterization analysis) were further analysed for anti-plant glycan antibodies. The samples originated from the following clinical trials: Study PB-06-001, ‘A Phase III Multicenter, Randomized, Double-Blind Trial to Assess the Safety and Efficacy of Two Parallel Dose Groups of Plant Cell Expressed Recombinant Human Glucocerebrosidase (prGCD) in Patients with Gaucher Disease’. ERT-naïve adult patients (N = 32) with a total treatment duration of 9 months (study started on August 2007 and ended September 2009) ClinicalTrials.gov NCT00376168 [37]. Study PB-06-002, ‘A Phase 3 Multicenter, Open-label, Switchover Trial to Assess the Safety and Efficacy of Plant Cell Expressed Recombinant Human Glucocerebrosidase (Taliglucerase alfa) in Patients with GD Treated with Imiglucerase (Cerezyme^®^) Enzyme Replacement Therapy.’ Adult and pediatric patients (N = 26 and 5, respectively) with a total treatment duration of 9 months (study started on December 2008 and ended January 2013) ClinicalTrials.gov: NCT00712348 [38]. Study PB-06-005, ‘A Multicenter, Double-blind, Randomized Safety and Efficacy Study of Two Dose Levels of TGA in Pediatric patients with Gaucher Disease’. Pediatric patients (N = 11) with a total treatment duration of 12 months. (study started on August 2010 and ended July 2012) ClinicalTrials.gov: NCT01132690 [39]. For all 3 studies, a power analysis was used to calculate sample size. The majority of the protocol deviations in these studies were due to visits outside of scheduled window or study procedures that were not performed according to protocol schedule. One patient from study PB-06-001, who had a major protocol violation (positive urine pregnancy test), was discontinued from the study after Visit 10 (Week 18) and was excluded from the study population. None of the protocol deviations affect data analysis. A Sponsor review of the complete list of Protocol Deviations concluded that none of the protocol deviations significantly affected the completeness, accuracy, and/or reliability of the study data, nor were the subject's rights, safety, or well-being affected. A consort representing these three studies is shown in Fig 1. All additional data on these clinical trials are available at https://clinicaltrials.gov/.

### Glycosylation analysis of HRP

The plant glycoprotein HRP contains glycans with a high similarity to the plant glycan structures that are found on TGA. Since HRP has a distinct protein backbone that differs from TGA, but has glycans similar to TGA, antibodies that react both with HRP and TGA are considered specific for the common plant glycan structures. To verify that the HRP used in this testing contains glycans similar to TGA, we analysed several lots of HRP for their glycan composition. Glycan analysis of HRP was based on the method described by Tekoah et al. [32], which was also used for glycan analysis of TGA. In short, HRP proteins (~200 μg) were separated on sodium dodecyl sulphate polyacrylamide gel electrophoresis (SDS-PAGE) following reduction and alkylation. N-linked glycans were released from gel slices by incubation with trypsin, followed by peptide N-glycosidase A [40]. The glycans were fluorescently labelled with 2-aminobenzamide and run on normal phase (NP) HPLC (Waters, Milford, MA). Simultaneous exoglycosidase sequencing of the released glycan pool was performed as described previously [40]. The retention times of the individual glycans were compared with those of a standard partial hydrolysate of dextran, giving a ladder of glucose units. The following exoglycosidases were used: Xanthomonas β-1,2-xylosidase (Calbiochem, San Diego, CA), bovine testes β-galactosidase, bovine kidney α-fucosidase, Jack bean β-hexosaminidase, and Jack bean α-mannosidase (Prozyme, Hayward, CA) at concentrations suggested by suppliers. Results were compared with an internal and a public database [41] for the assignment of glycan structures. Results were reported for glycans above 1% relative abundance.

### Enzyme-linked immunosorbent assay (ELISA) for detection of plant-derived glycans

A competitive ELISA based assay was developed in order to detect antidrug antibodies (ADA) specific for the glycan moieties on taliglucerase alfa. In brief, serum samples are diluted at minimal required dilution (MRD, 1:30) and pre-incubated in the presence or absence of 200 μg/mL HRP, for 1 hour. Following the pre-incubation step, samples are loaded on microtiter plates, pre-coated with taliglucerase alfa and blocked with 2% bovine serum albumin (BSA), and incubated for an additional 2 hours at RT. Following a wash step, a recombinant Protein A/G-Alkaline Phosphatase (AP) Conjugate (cat# 32391, Pierce, Rockford, IL) is added at a 1:10000 dilution and incubated for 1.5 hours at RT. After an additional washing step, a phosphatase substrate (BluePhos Cat # 50-88-00, KPL, Gaithersburg, MD.) is added and incubated for approximately 20 min. Following the addition of a stop solution (AP stop solution cat #50-89-00, KPL, Gaithersburg, MD) the intensity of the color produced is measured by OD at 630 nm and is proportional to the level of the bound antibody. All dilution steps are performed using 1% BSA.

### Statistics

The population distribution, log transformation and outlier identification was performed using JMP software. Cut-point calculation was performed according to standard recommendations [42] on Microsoft Excel and clinical data analysis was performed using SAS software.

## Results

### Immunogenicity testing

A multi-tiered approach was used for immunogenicity evaluation of patient samples. Specifically, all samples from the clinical trials were first screened for total anti-drug antibodies (ADA) using a screening assay, then confirmed using a separate assay. The screening and the confirmatory assays could detect all ADA, but did not distinguish between antibodies to the protein backbone or to the glycan moieties. The confirmed anti-TGA ADA positive samples were further characterized using a competitive assay, with HRP as a competitor, in order to evaluate the presence of antibody specificity to plant glycans.

All the assays for immunogenicity testing were validated according to recommendations of Shankar et al and the FDA Guidance for Industry Assay Development for Immunogenicity Testing of Therapeutic Proteins recommendations [42, 43].

### Assay development and validation

#### Characterization of HRP glycans

HRP is a plant glycoprotein that has been reported to contain glycans with a high similarity to the plant glycan structures that are found on TGA. Furthermore, since HRP has a distinct protein backbone that differs from TGA, but has glycans similar to TGA, antibodies that react both with HRP and TGA may be concluded to be specific for the common plant glycan structures. To verify that the HRP used in this testing contains glycans similar to TGA, we analysed several lots of HRP for their glycan composition. Table 1 summarizes the main glycans (above 1% relative abundance) found in a representative batch of TGA and HRP. The most abundant glycan in both proteins was found to be a tri-mannose glycan with a core α(1,3)-fucose and a bisecting β(1,2)-xylose (~90% of the total glycan pool).

**Table 1:** Glycan composition of TGA and HRP.

Glycan acronym ^a^		F(3)M2X	M3X	F(3)M3	F(3)M3X	F(3)XA1	F(3)M4X
Protein	Batch number	Relative percent of glycans					
**TGA**	**148694**	5	5	ND ^b^	90	ND	ND
**HRP**	**Cat#P8250/lot** **#070M1621V**	ND	4	4	88	1	3

^a^ Glycan acronym: A1 = (GlcNAc)Man_3_-GlcNAc_2_; F(3) = α(1,3) linked core Fucose; M = mannose; Mx = Man_x_-GlcNAc_2_; X = β(1,2) linked bisecting xylose.

^b^ ND: not detected below 1% of total glycan pool.

#### Assay format

**Fig 2:**
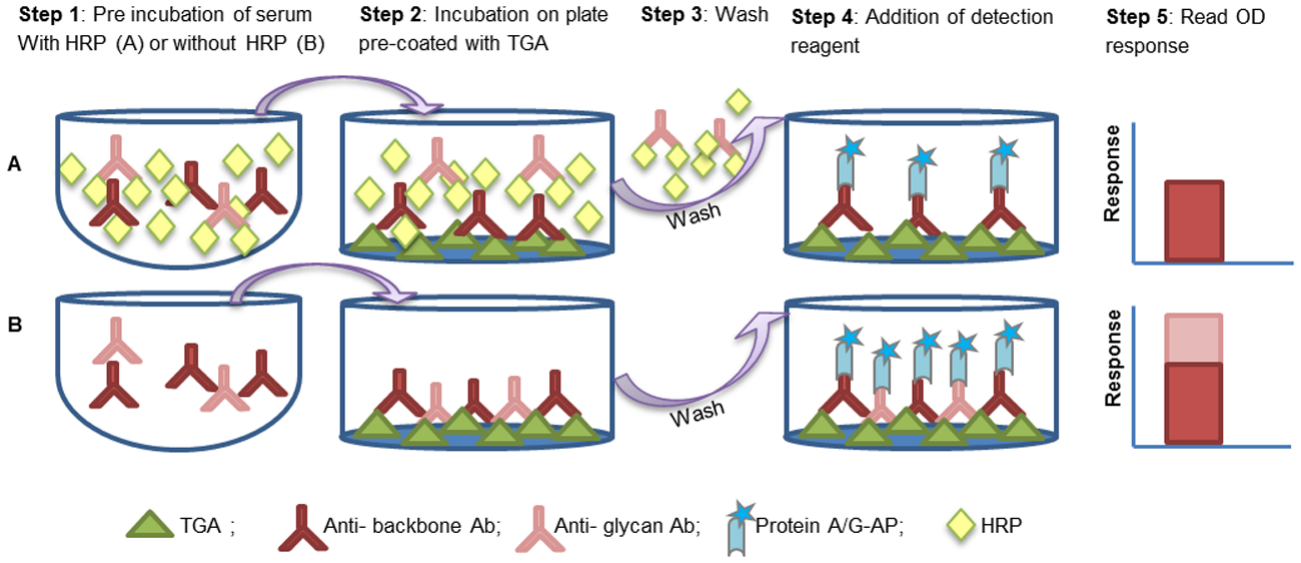
Schematic representation of the assay. A stepwise format (1–5) was developed for the binding of serum antibodies to TGA (A) with HRP and (B) without prior HRP incubation, showing a response reduction in the presence of HRP.

A competitive ELISA format was implemented using HRP as a competitor for the detection of antibodies specific to plant glycans moieties on TGA. Aliquots of each serum sample were pre-incubated, either with or without the HRP competitor, and then loaded on a TGA-coated ELISA plate. During the pre-incubation step, the antibody population targeted against the plant glycans on TGA could bind to the HRP glycans. The antibodies associated with HRP were then blocked from binding to TGA on the pre-coated wells and washed out in the wash step, leading to a reduction in the OD response, compared to the parallel aliquot pre-incubated without HRP (Fig 2). The reduction was calculated as percent HRP immunodepletion as follows:

$$\%HRP\;immunodepletion\;=\;100\times (1-{OD}_{with\;HRP}/{OD}_{without\;HRP}).$$

#### Establishment of assay positive and negative controls

About 40 different anti-glucocerebrosidase polyclonal and monoclonal antibodies and commercially available anti-plant glycan antibodies were tested as candidates for negative controls (NC) and positive controls (PC) for the assay. NCs were defined as TGA-binding antibodies that interact with the TGA backbone, but do not recognize the plant glycan structures on the protein. PCs were defined as TGA-binding antibodies that interact with the plant glycan structures of TGA, but do not recognize the protein backbone.

**Fig 3:**
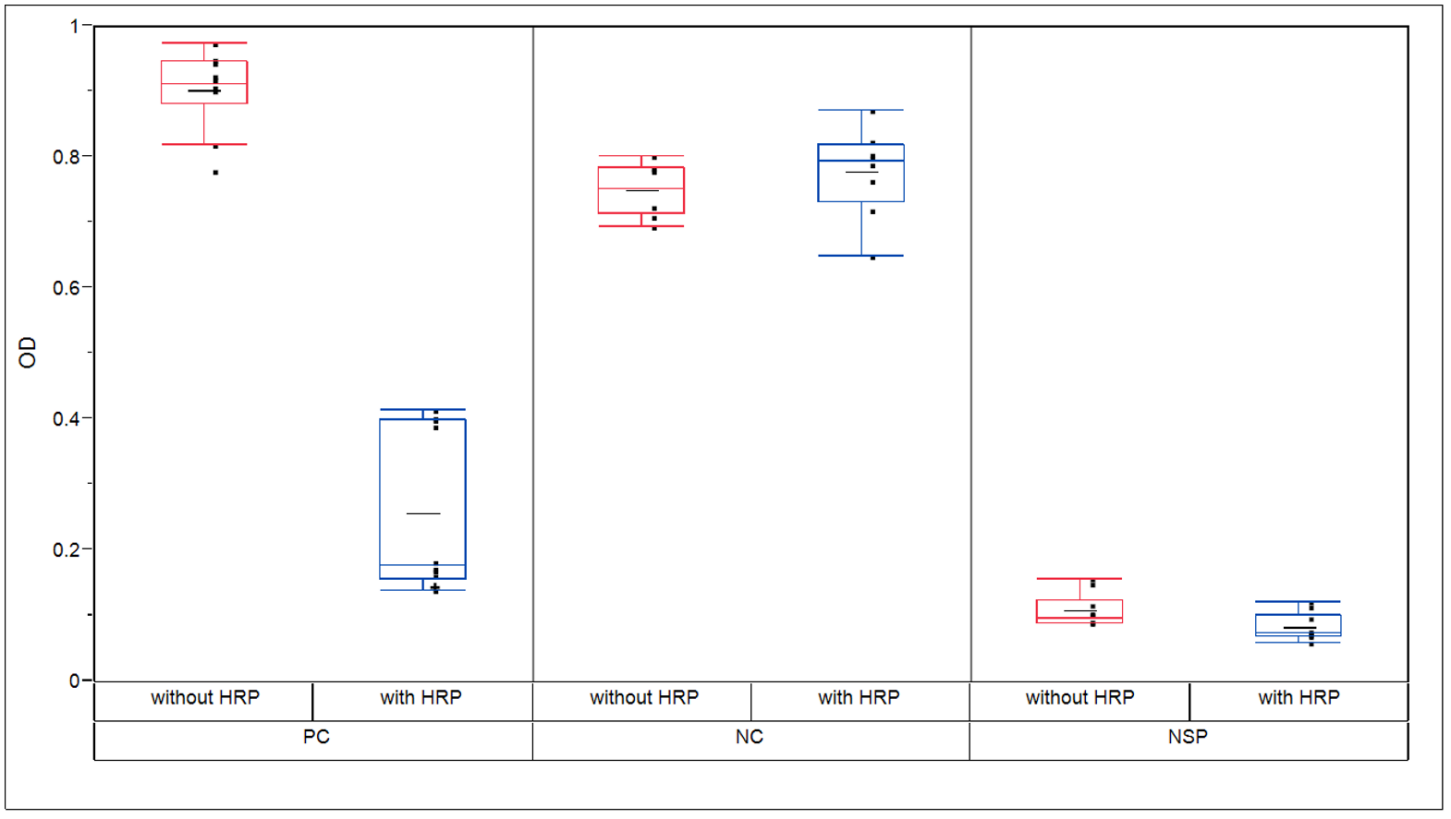
Interaction of assay controls with TGA with and without HRP. Assay controls were tested on TGA coated plates, with and without HRP competitor and mean absorbance was measured by OD. PC showed high initial OD and inhibition of ~80% with the addition of HRP, indicating recognition of plant glycans. NC showed high OD, both with and without HRP, indicating recognition of protein backbone. Normal serum pool (NSP) represented serum background levels with low OD, both with and without HRP, indicating low reactivity to TGA. Results, presented as a box plot graph, include data from 3 independent runs, showing individual measurements together with mean.

Following the PC screening process a commercially available anti-HRP antibody (see Materials) produced in rabbits (Rb anti-HRP) was selected. This PC antibody had the ability to bind both TGA (resulting in high OD in the assay when pre-incubated in the absence of HRP) and HRP (resulting in significantly lower OD when pre-incubated in the presence of HRP). The inhibition of the PC by HRP reached about 80% (Fig 3), indicating that the antibody interacted with the plant glycans and not the protein backbone of TGA. The NC screening process identified some monoclonal anti-TGA antibodies that interacted specifically with TGA, but not with HRP. The chosen NC showed no inhibition by HRP (Fig 3). This indicated that the NC antibody reacted with the protein backbone of TGA and not the plant glycans.

The PC and NC were spiked into a normal serum pool (NSP) sample, in order to simulate the patient serum samples. For evaluation of background response, we tested the reactivity of un-spiked NSP with TGA and its inhibition by HRP. As can be seen in Fig 3, low OD values were measured, indicating a low response to TGA by factors that may be present in NSP.

#### Assay cut-point determination

The assay cut-point is defined as the level of response at or above which a sample is defined to be positive and below which it is defined to be negative [44]. In order to determine the cut-point for our assay, 52 serum samples from healthy individual subjects were tested in three independent runs and measured in duplicates with and without HRP.

**Fig 4:**
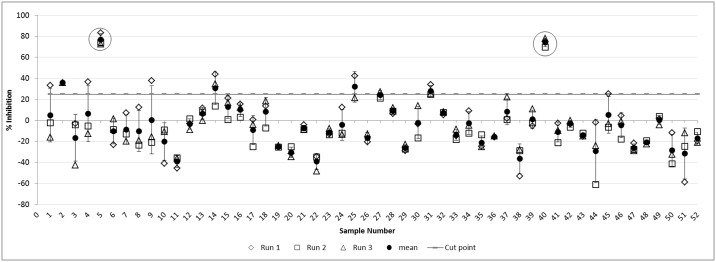
Assay cut-point determination. Percent (%) Immunodepletion by HRP of healthy individual serum samples. The data show three independent runs and their mean± standard deviation. Individuals 5 and 40 (highlighted) were identified as outliers, and were excluded from the calculation.

The assay cut-point was calculated based on the method described by Shankar et al. [42] for calculation of a cut-point for a competitive inhibition test format. Briefly, the logarithm of the ratio of the OD response, of the sample with HRP versus the sample without HRP was calculated to give the log ratio value for each individual sample. These values were used to identify outliers and to calculate the assay cut-point (based on HRP immunodepletion). The values of the log ratio for each run (52 serum samples) were analysed for population distribution using JMP software and outliers were identified using the outlier boxplot tool. Outliers from each data set (run) were excluded and re-analysis was performed until no outliers remained in the data set. A total of 13 individual measurements, out of the 156 measured (52×3) were excluded as outliers. Of these 13 individual measurements, 6 measurements, representing 2 individuals, were totally excluded from the statistical analysis (red data points in Fig 4). The mean and standard deviation (SD) of the log ratio of the remaining 143 results (representing 50 individuals) were calculated to give values of 0.022 and 0.089, respectively. These values were further used to calculate the assay cut-point, based on a one sided confidence interval of 95% (1.645×SD) of the log ratio. Finally, the cut-point for this assay was calculated as 100 × (1 − *antilog*(*Mean* − 1.645 × *SD*)) and found to be 24.98% immunodepletion.

#### Validation of assay parameters

Assay precision, robustness and sensitivity were measured as part of the method validation. Assay precision was estimated for both intra-run and inter-run and was measured as percent coefficient of variation (% CV). For intra-run, 6 duplicate sets of the assay controls (high, medium and low PC concentrations, NC and NSP) were each tested in a single validation run. For inter-runs precision testing, 1 duplicate set of each of the above assay controls was tested in all validation runs. Intra-run precision was 12.24% CV or lower and the overall precision of the inter-runs (a total of 16 runs) was calculated as 23.86% CV or below.

Robustness was assessed by comparing different plate readers, plate washers and performance by different analysts. All robustness measurements of the PC and NC mean OD values met the acceptance criteria of within ±30% difference set for the assay. Results indicated that the assay was robust under the conditions tested.

The assay sensitivity is the lowest concentration at which antibodies specific to plant glycans present on TGA are still detectable (i.e. sample yields an OD value above the mean OD value of plate NSP controls, and at or above the assay cutpoint). Assay sensitivity was assessed by testing serial dilutions of the high PC from an initial concentration of 1000 ng/mL down to 7.8 ng/mL; each series was tested in 3 independent runs. The sensitivity of the assay was calculated for each run and the mean value of 64.27 ng/mL (ranging between 55.85–77.04 ng/mL) was determined as the assay sensitivity based on the PC.

#### Competitive immunodepletion ELISA for detection of antibodies to plant-derived glycans

TGA was bound to the ELISA plate and HRP, which contains similar plant glycan structures as in TGA, was used as a competitor in an immunodepletion step; antibodies specific to plant glycans bound to the competitor (HRP) in a pre-incubation step, which prevented these antibodies from binding to the TGA coated plate.

Serum samples were first diluted at minimal required dilution (1:30), using 1% bovine serum albumin (BSA) in PBS, and then separate aliquots were pre-incubated in the presence or absence of 200 μg/mL HRP for 1 hour at room temperature. Following the pre-incubation step, samples were loaded and incubated for 2 hrs at RT on ELISA plates that were previously coated overnight with 0.5μg/mL TGA (in 50 mM carbonite/bicarbonate buffer) and blocked with 2% bovine serum albumin (BSA). Following a wash step, a recombinant Protein A/G-Alkaline Phosphatase (AP) Conjugate (cat# 32391, Pierce, Rockford, IL) was added at a 1:10,000 dilution and incubated for 1.5 hours at RT. After an additional wash step, a phosphatase substrate (BluePhos Cat # 50-88-00, KPL, Gaithersburg, MD.) was added and incubated for approximately 20 min. Following the addition of a stop solution (AP stop solution cat #50-89-00, KPL, Gaithersburg, MD) the intensity of the colour produced was measured by optical density (OD) at 630 nm and was proportional to the level of the plate-bound antibody.

#### Assay validation

A competitive inhibition assay format was implemented using HRP as a competitor for the detection of antibodies specific to plant glycan moieties on TGA, since it was found to have similar glycans to TGA. Assay details can be found in the Methods section. Negative controls (NC) and positive controls (PC) for the assay were established (Fig 3) and the assay cut-point was calculated and found to be 24.98% immunodepletion (by HRP), with a sensitivity of 64.27 ng/mL based on the PC.

Using this assay, the data from 52 healthy individuals was evaluated by 3 independent runs (Fig 4). Seven subjects (13.5%) demonstrated the presence of pre-existing anti-glycan antibodies.

### Measurement of anti-plant glycan antibodies in clinical study samples

Samples from 3 different phase III clinical studies were evaluated for the presence of anti-plant glycan antibodies. For the analysis, 2 categories were used, ERT-naïve and ERT-experienced patients, since these separate groups may have a different antibody pattern (pre-existing/baseline, or treatment induced ADA and/or anti-plant glycans antibodies). Two studies, PB-06-001 and PB-06-005, included ERT-naïve patients (adult and pediatric, respectively) and a third switchover study, PB-06-002, included ERT-experienced patients that were previously treated with another β-glucocerebrosidase drug, imiglucerase.

**Fig 5:**
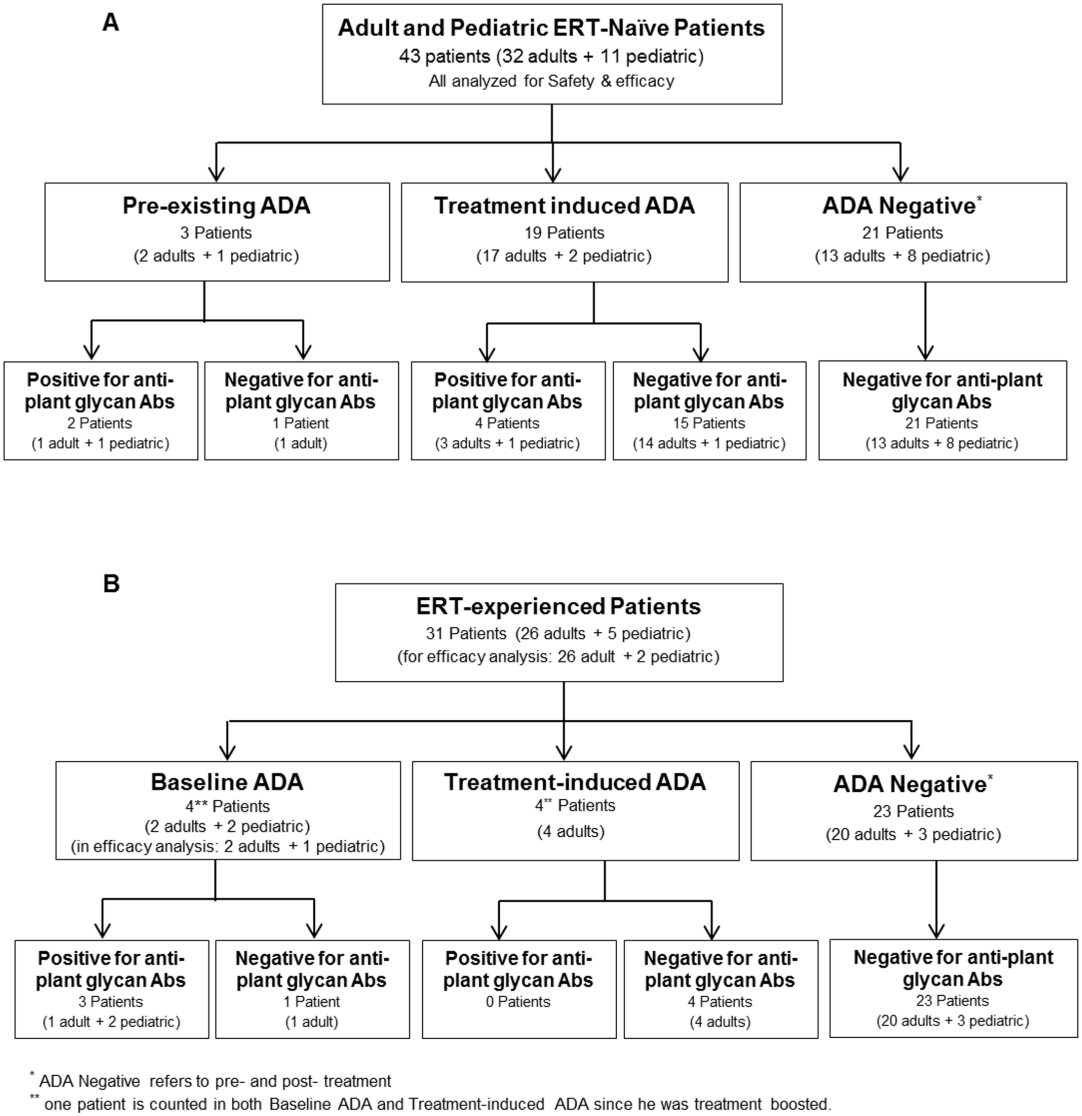
Total ADA and anti-plant glycan antibodies prevalence found in GD patient population treated with TGA. (A) Data evaluated from ERT-naïve patient samples and (B) ERT-experienced patient samples. *One patient of the ERT-experienced group, was counted for both baseline ADA and treatment induced ADA, since he was ADA positive at baseline and had a treatment-boosted following treatment with TGA (had ≥6-fold higher titer after TGA treatment).

Patients were considered positive for ADA if they had at least one sample positive for ADA. The ADA positive samples were further assessed for anti-plant glycan antibodies. Fig 5A and 5B summarize the assessment of samples that contained anti-plant glycan antibodies in ERT-naïve and ERT-experienced patients, respectively.

#### ERT-naïve patients

In clinical trials of ERT-naïve patients (n = 43: 32 adults and 11 pediatric), 3 tested positive for ADA at baseline, prior to TGA treatment (Fig 5A) of which, 2 were identified as positive for antibodies to the plant glycans (5% of the entire naïve population n = 43). These 2 patients had low ADA titers and they became negative for ADA with time. An additional 19 patients developed ADA following the treatment with TGA. Of the 19, who developed ADA during TGA treatment, only 4 had detectable antibodies to the plant glycans (9% of the entire naïve population).

#### ERT-experienced patients

The clinical trial of patients who switched from imiglucerase to TGA therapy included 31 patients (26 adult and 5 pediatric), of which, 4 tested positive for ADA (any TGA epitopes) at baseline, prior to treatment with TGA (Fig 5B). Further characterization of these serum samples showed that 3 of the 4 tested positive for antibodies to the plant glycans at baseline. These 3 patients were ADA positive prior to treatment with TGA and became ADA-negative after the switch to TGA. The fourth patient had an ADA treatment-boosted response [45] after the switch to TGA, but tested negative for antibodies to the plant glycans. This patient was counted twice (both in the baseline ADA positive and in the treatment induced ADA groups). In addition, 4 patients developed ADA during treatment with TGA (treatment induced ADA). All of them tested negative for antibodies to the plant glycans.

### Impact of anti-glycan antibodies on efficacy and safety of TGA

Of a total of 74 patients analysed for ADA, 71 were evaluated for the clinical safety and efficacy to assess the impact of anti-plant glycan antibody positive samples. The data of the additional 3 ERT-experienced pediatric patients were not available at the time of safety and efficacy analysis, but these patients were evaluated separately.

#### Clinical safety

The treatment-related and all causality adverse events (AEs) experienced by patients who were found to be positive for anti-plant glycan antibodies (n = 8) were compared with patients who were negative for anti-plant glycan antibodies (n = 63), i.e. those who were negative for anti-TGA ADA or had no detected antibodies to plant-glycans.

In patients found negative for anti-plant glycan antibodies, treatment-related AEs were Hypersensitivity (n = 4), Infusion related reaction (n = 4), Headache (n = 4), Pruritus (n = 3) and Arthralgia (n = 2), but, there were no treatment-related AEs in the patients who were found positive to anti-plant glycan antibodies. Therefore, the presence of detectable anti-plant glycan antibodies was not associated with a discernible trend in treatment-related AEs.

All causality AEs experienced by the patients who were found positive to anti-plant glycan antibodies, were in greater frequency than amongst patients considered to be negative to anti-plant glycan antibodies. These AEs included Diarrhoea (n = 2, 25% vs. n = 8, 12.7%), Vomiting (n = 3, 37.5% vs. n = 8, 12.7%), Influenza (n = 2, 25% vs. n = 7, 11.1%), Arthralgia (n = 3, 37.5% vs. n = 13, 20.6%), Pain in extremity (n = 2, 25% vs. n = 11, 17.5%) and Headache (n = 3, 37.5% vs. n = 12, 19%).

Although the presence of detectable anti-plant glycan antibodies appeared to be associated with a greater frequency of certain all causality events, the low number of patients in anti-plant glycan antibody positive group and the low number of events makes comparison challenging because a single occurrence of an AE has a large effect on the calculation of frequency. With regards to treatment related AEs, it should be noted that the presence of detectable anti-plant glycan antibodies was not associated with a discernible trend in treatment-related AEs and no clear demonstrated correlation between the appearance of anti-plant glycan antibodies and an increased clinically relevant risk associated with immunogenicity was observed.

The safety data of the three additional patients, whose data were not available at the time of safety and efficacy vs. anti-plant glycan antibodies analysis, show a very small number of AEs (headache, cough and upper respiratory tract infection) in 2 out of the three patients and all were reported as non-treatment related.

#### Clinical efficacy

GD clinical symptoms include hepatosplenomegaly, anaemia, thrombocytopenia and skeletal deterioration which resulted from glucosylceramide accumulation in the lysosomes of macrophages found in the liver, spleen and bone marrow [46, 47]. The primary efficacy endpoint of the ERT-naïve patients (studies PB-06-001 and PB-06-005) was reduction in spleen volume. The major secondary end points included improvement in anaemia, thrombocytopenia, and reduction in liver volume. The efficacy endpoints of the ERT-experienced patients (PB-06-002) included stability in spleen volume, anaemia, thrombocytopenia and liver volume.

A post-hoc assessment of absolute and percent change from baseline in the 4 efficacy endpoints (spleen volume, liver volume, hemoglobin and platelet count) was performed. Although, the group of patients tested positive for anti-plant glycan antibodies was rather small in size (n = 8), it was still clear, as shown in Table 2, that these patients had a similar efficacy pattern compared to the ADA negative patients. This indicates that the presence of anti-plant glycan antibodies had no influence on the efficacy of TGA treatment.

**Table 2:** Efficacy parameters and mean response by anti-plant glycan antibody—Status of available data at end of study.

Efficacy Parameter	ERT-naïve				ERT-experienced	
	adults		pediatric		adults & pediatric ^a^	
	PB-06-001		PB-06-005		PB-06-002	
	Anti-Glycan ADA					
	Positive	Negative	Positive	Negative	Positive	Negative
**Spleen Volume**						
**Patients**	n = 4	n = 25	n = 2	n = 9	n = 2	n = 20
**Absolute Change from Baseline (mL)**	-569	-689	-659	-402	-5.1	-70.9
**% Change from Baseline**	-31.2	-33.1	-47.0	-31.4	-0.5	-6.6
**Liver Volume**						
**Patients**	n = 4	n = 25	n = 2	n = 9	n = 2	n = 23
**Absolute Change from Baseline (mL)**	-373	-302	-207	-99.2	74	-78.2
**% Change from Baseline**	-13.1	-10.4	-21.6	-7.1	5.3	-4.0
**Hemoglobin**						
**Patients**	n = 4	n = 25	n = 2	n = 9	n = 2	n = 25
**Absolute Change from Baseline (gr/dL)**	2.4	1.8	1.9	1.4	-0.6	-0.2
**% Change from Baseline**	25.9	17.3	18.9	13.8	-4.7	-1.7
**Platelet Count**						
**Patients**	n = 4	n = 28	n = 2	n = 9	n = 2	n = 25
**Absolute Change from Baseline (/mm3)**	5500	29332	68500	55444	-7500	-2351
**% Change from Baseline**	10.5	48.0	86.7	42.3	-3.3	-1.4

^a^* Not including last 3 patients.

#### ERT-Naïve patients

The assessment of the major clinical efficacy endpoints versus anti-plant glycan antibodies status at end of study was done for the ERT-naïve adults (n = 32, 9 months) and pediatric (n = 11, 12 month) patients. The clinical efficacy data was evaluated versus the anti-plant glycan status. The anti-plant glycan positive group included 6 patients (4 adults and 2 pediatric) and the negative group included 37 patients (28 adults and 9 pediatric).

The results show improvement (reduction) of the spleen and liver volume in pediatric and adult patients in both the positive (n = 6) and negative (n = 37;) anti-plant glycan groups. In the adult patients the reduction of spleen and liver volume was similar for both groups. In the ERT-naïve pediatric patients positive for anti-plant glycan antibodies, a higher reduction in spleen and liver volume was shown compared to the negative group (Table 2).

Hemoglobin levels show a similar improvement (increase) in pediatric and adult patients in both the positive and negative anti-plant glycan groups.

Improvement of platelet count (increase) was shown in pediatric and adult patients in both the positive and negative anti-plant glycan groups. In the adult patients, positive for anti-plant glycan antibodies, the increase was lower than the negative group. The pediatric patients, positive for anti-plant glycan antibodies were found to have a higher increase compared to the negative group.

#### ERT-experienced patients

A similar assessment was done for the ERT-experienced adults (n = 26) and pediatric (n = 5) patients (9 months). The anti-plant glycan positive group included 3 patients (1 adult and 2 pediatric) and the negative group included 28 patients (25 adults and 3 pediatric).

The results show stability, with changes of less than 10%, of the spleen volume, liver volume, hemoglobin and platelet count in ERT-experienced patients in both the positive and negative anti-plant glycan groups.

The efficacy data of the three additional patients, whose data were not available at the time of safety and efficacy analysis versus anti-plant glycan antibodies status, show stability to slight improvement in hematological parameters (platelet count and hemoglobin) and organ volume (spleen and liver).

## Discussion

Different factors may elicit unwanted immunologic responses to biotherapeutic drugs. Immunogenicity of non-mammalian plant glycans on therapeutic glycoproteins and vaccines produced in plants was studied and discussed in previous articles [13, 29, 30]. The glycan moieties are the main structural difference between taliglucerase alfa and the other two ERTs approved for treatment of GD, which are produced by cultured mammalian cells [32]. Concern has been expressed that plant recombinant products, such as taliglucerase alfa, might be more immunogenic than the ERTs produced in mammalian cells because of their unique plant glycan structures. In order to properly evaluate the prevalence of anti-plant glycan antibodies in the healthy population, and the impact of plant glycans on immunogenicity post-treatment with the plant-derived biotherapeutic drug, we developed and validated a precise and sensitive ELISA for measurement of antibodies directed against the plant glycan structures on TGA. The ELISA was based on an immunodepletion step, using HRP as a competitor. HRP was chosen since it contains plant specific glycan structures [48] also present on TGA (Table 1) but has a distinct protein backbone, and thus its presence leads to a depletion of antibodies specific to the plant glycan structures.

We identified a negative control antibody that specifically binds the TGA backbone and does not recognize the plant glycan structures on the protein and a positive control (anti-HRP) that interacts only with the plant specific glycans and not the protein backbone of TGA. For demonstrating specificity, we used different plant derived proteins (HRP and TGA) as competitors and different β-glucocerebrosidase molecules lacking plant glycan structures to capture anti-TGA antibodies, in order to confirm that the method only detects antibodies specific to the plant glycans. Rigorous statistical methods were used to establish the cut-point for distinguishing positive and negative samples. In doing so, we were able to demonstrate that the optimized assay is robust, precise and sensitive and is appropriate for the measurement of anti-TGA antibodies directed against the plant glycan structures on TGA.

In the healthy population, we were able to show, using the validated method, that 13.5% of the subjects were positive for the presence of anti-plant glycan antibodies. Our data are consistent with some previous publications, showing that less than 20% of the normal healthy population has detectable levels of pre-existing antibodies to plant glycan motifs [29, 30], but, inconsistent with an earlier study by Bardor et al. [13] reporting that about 50% of non-allergic blood donors have antibodies specific for core β(1,2)-xylose in their sera, whereas 25% have antibodies against core α(1,3)-fucose. These high percentages should be addressed with caution, bearing in mind that they were acquired using an un-validated method, while taking into consideration that this can also be due to differences in assay format.

In the ADA results analysis, we considered the different population groups (adult/pediatric; naïve/switchover), and whether or not the patient was ADA positive pre- or post-treatment with TGA.

The observation of baseline ADA assay positive results in some subjects prior to exposure to biotherapeutic drugs is not unusual, especially when sensitive assays are used; however, the impact of pre-existing antibodies on post-treatment immune response, as well as on safety and efficacy is not consistent across different biotherapeutics [49, 50].

For 5 out of 74 GD patients (6.8%) that were positive for ADA and anti-plant glycan antibodies at baseline, post-treatment ADA titters were either stable or reduced following exposure to TGA. In an additional evaluation of clinical outcomes and AEs, these 5 patients did not show different trends of safety and efficacy parameters, compared to the remaining study population. Although based on a small number of patients with pre-existing antibodies, these results suggest that some pre-existing antibodies are specific for plant glycan structures on TGA. This result (6.8%) is somewhat lower than what would be expected, since the healthy population evaluated in this study showed a total of 13.5% of the total subjects having pre-existing anti-plant glycan antibodies. This difference could result from immune dysregulation shown in GD patients [51]. In addition, the presence of these antibodies does not appear to increase risk of immune response to the plant derived drug based on the lack of observed post-treatment boosting of the ADA levels and based on safety and efficacy results.

Samples from 23 patients were found positive for treatment-induced ADA, of which 1 also had pre-existing ADA. Out of these patients, only 4 (5.4% of all 74 patients) were positive at one or more time points for antibodies to plant glycans. These were mostly associated with ADA titers lower than the median titer of 215.

Despite these low numbers, we tried to correlate safety and efficacy data with the presence of anti-plant antibodies. TGA shows equivalent, or better safety, when comparing between the three alternative β-glucocerebrosidase ERT treatments [52]. The presence of anti-plant glycan antibodies was not associated with treatment-related AEs and no clear correlation to increased clinical risk was observed. Assessment of efficacy data indicated no relationship between the presence of anti- plant glycan antibodies and the efficacy end-points for all types of patients.

To conclude, we developed a validated method for detection of antibodies directed specifically against plant glycans to assess the abundance of anti-plant glycan antibodies in the healthy human population. Furthermore, we were able to assess the presence of these antibodies in ADA positive TGA-treated GD patients, and correlate the results with safety and efficacy parameters. Based on these results, the prevalence of anti-plant glycan antibodies in the total population was found to be under 15% and does not increase after exposure to plant derived biotherapeutics. Furthermore, this response appears to be limited in magnitude and impact on safety and efficacy.

The data that was collected in taliglucerase alfa studies in general [37–39] and in particular in the current study, indicates that plant recombinant products are not more immunogenic than the mammalian ERTs despite their unique plant glycan structure. This was shown by the low prevalence of anti-plant glycan ADA as well as by the lack of correlation with efficacy reduction or with adverse events. In addition, a recent study by Kishnani and co-authors [53] showed that AEs associated with antibody positivity, in infusion associated reactions, did not occur with greater prevalence in taliglucerase alfa treated patients, compared to the other 2 ERTs for GD. In addition, it should be noted that most patients treated with TGA did not suffer adverse effects or loss of therapeutic benefit, regardless of presence ADA.

Limitations of this study are due to the small sample size, including a total of 74 patients. Of which, only few were positive for anti plant glycan antibodies (pre- and post- treatment). Therefore, further evaluation of plant glycan immunogenicity risk using a validated analysis method is needed for other biotherapeutics. Albeit, our results suggest that plant glycan structures do not pose a greater immunogenicity risk than any other epitope. Thus, plant derived biotherapeutics can be efficiently and safely given to humans.

## Supporting information

S1 File Protocol for clinical trial PB-06-001. (PDF)

S2 File Protocol for clinical trial PB-06-002. (PDF)

S3 File Protocol for clinical trial PB-06-005. (PDF)

S4 File Data set of anti-drug antibody results. (PDF)

S5 File CONSORT checklist. (PDF)
