# Supplementary material for: Immunogenicity of glycans on biotherapeutic drugs produced in plant expression systems—The taliglucerase alfa story
Source: PLoS One. 2017 Oct 31;12(10):e0186211. doi: 10.1371/journal.pone.0186211 (PMC5663370; doi:10.1371/journal.pone.0186211)
Supplement: S1 File — (PDF) [file pone.0186211.s001.pdf]

Protocol Title: A Phase III Multicenter, Randomized, Double-Blind Trial to Assess the Safety and Efficacy of Two Parallel Dose Groups of Plant Cell Expressed Recombinant Human Glucocerebrosidase (prGCD) in Patients with Gaucher Disease

Protocol Number: PB-06-001

Sponsor:
Protalix Biotherapeutics
2 Snunit street
Science Park
POB 455
Carmiel 20100, Israel
Ph: 972-4-988-9488
Fax: 972-4-988-9489

CRO:
Target Health Inc.
261 Madison Avenue, 24th Floor
New York, NY 10016
Ph: (212) 681-2100
Fax: (212) 681-2105

Cato Research Ltd.
2, Habarzel Street, 4th Floor
Ramat Hahayal
Tel Aviv, Israel 69710

Coordinating Investigator
Ari Zimran, M.D.
Associate Professor of Medicine
Director, Gaucher Clinic
Shaare Zedek Medical Center
Jerusalem, 91031, Israel
Ph: 972-2-6555673
Fax: 972-2-6517979

Study Sites: Multicenter

Confidentiality Statement
The confidential information in this document is provided to you as a Principal Investigator or consultant for review by you, your staff, and the applicable Institutional Review Board/Independent Ethics Committee. Your acceptance of this document constitutes agreement that you will not disclose the information contained herein to others without written authorization from the Sponsor, Protalix Biotherapeutics.

1. Protocol Synopsis

Title of Study	A Phase III Multicenter, Randomized, Double-Blind Trial to Assess the Safety and Efficacy of Two Parallel Dose Groups of Plant Cell Expressed Recombinant Human Glucocerebrosidase (prGCD) in Patients with Gaucher Disease
Study Number	PB-06-001
Investigational Product	Plant cell expressed recombinant human glucocerebrosidase (prGCD)
Indication	Gaucher disease
Study Sites/Location	Multicenter
Objectives	The objective of this study is to assess the safety and efficacy of prGCD in patients with significant signs and symptoms of Gaucher disease not treated with enzyme replacement therapy
Study Design	This will be a multi-center, randomized, double-blind, parallel group, dose-ranging trial to assess the safety and efficacy of prGCD in 30 untreated patients with Gaucher disease. Patients will receive IV infusion of prGCD every two weeks at the selected medical center. The duration of treatment will be nine months. At the end of the 9-month treatment period (21 visits, 38 weeks) eligible patients will be offered enrollment in an open-label extension study.
Number of Patients	30 patients
Diagnosis and Main Criteria for Inclusion	Key Inclusion Criteria:<br>• Males and females, 18 years or older<br>• Confirmed enzymatic diagnosis of Gaucher disease.<br>• Splenomegaly defined as greater than eight times the expected volume [measured volume divided by estimated volume (0.2% of body weight)] as determined by MRI volumetric analysis<br>• Female patients of child-bearing potential who agree to use a medically acceptable method of contraception, not including the rhythm method.<br>• Thrombocytopenia (defined as platelet counts below the lower limit of normal) and/or anemia (defined by hemoglobin level at least 1 g/dL below normal range according to sex and age).<br>• Patients who have not received ERT in the past

	or Patients who have not received ERT in the past 12 months and have a negative anti-glucocerebrosidase antibody<br>• Patients who have not received substrate reduction therapy (SRT) in the past 12 months.<br><br>Key Exclusion Criteria<br>• Currently taking another experimental drug for any condition<br>• Pregnant or nursing<br>• Presence of HIV and/or, HBsAg and/or hepatitis C infections<br>• Presence of any medical, emotional, behavioral or psychological condition that in the judgment of the Investigator would interfere with the patient's compliance with the requirements of the study.
Discontinuation from Study	Patients will be discontinued from treatment with study drug if:<br><br>• The patient requests to discontinue treatment<br>• Investigator feels that it is not in the best interest of the patient to continue treatment and/or if the investigator believes that the patient can no longer be compliant with the requirements of the study.<br><br>For any discontinuation, the Investigator will obtain all the required details and document the date and the main reason of the premature termination. If the reason for discontinuation is an AE, the specific event or the main laboratory abnormality will be recorded in the CRF. The Investigator will make thorough efforts to document the outcome. The Investigator will attempt to continue to follow the patient for the full duration of the study or at least for 30 days following discontinuation. If circumstances prevent the patient from completing all visits, every attempt will be made to complete all procedures listed in Section 11.8 Visit 20.
Treatment Groups	Treatment Group I: prGCD 30 units/kg<br>Treatment Group II: prGCD 60 units/kg
Drug Dosage and Route of Administration	prGCD 30 or 60 units/kg every 2 weeks by intravenous infusion over 1 hour
Duration of Treatment	38 weeks
Primary Efficacy Endpoint	Change from baseline in spleen volume measured by MRI at 9 months

Secondary Endpoint	Change from baseline in:<br>• Liver volume<br>• Platelet count<br>• Hemoglobin level<br>• Biomarkers (chitotriosidase and pulmonary and activation-regulated chemokine (PARC/CCL18)<br>• Proportion of patients with greater than 10% reduction in spleen volume at 9 months
Safety Endpoints	• Adverse events<br>• Clinical laboratory (hematology, biochemistry, urinalysis)<br>• Electrocardiogram<br>• Echocardiogram<br>• Pulmonary function tests
Statistical Analysis	

Table of Contents

1. Protocol Synopsis 2

2. Introduction 8

3. Objectives 9

3.1. Primary Efficacy Endpoint 9
3.2. Secondary Endpoints 9
3.3. Safety Endpoints 9
3.4. Additional Exploratory outcomes measurements 10
3.5. Pharmacokinetics 10

4. Principal Investigator 10

5. Institutional Review Board 10

6. Informed Consent 10

7. Patient Population, Number of Patients and Study Centers 11

8. Treatment Groups 11

9. Randomization 11

10. Patient Selection 11

10.1. Inclusion Criteria 11
10.2. Exclusion Criteria 12

11. Study Design 12

12. Study Visits 13

12.1. Screening (Visit 0, Day -21 ± 7) 13
12.2. Visit 1 (Baseline, Day 1) 13
12.3. Visits 2 – 6 (Weeks 2, 4, 6, 8, 10 ± 3 Days) 14
12.4. Visit 7 (Month 3, Week 12 ± 7 Days) 14
12.5. Visits 8 – 13 (Weeks 14, 16, 18, 20, 22, 24 ± 3 days) 15
12.6. Visit 14 (Month 6, Week 26 ± 7 days) 15
12.7. Visit 15 – 19 (Weeks 28, 30, 32, 34, 36 ± 3 days) 15
12.8. Visit 20 (Month 9, Week 38 ± 7 days) 16

13. Study Medication 16

13.1. Dosage 16
13.2. Formulation 17
13.3. Study Drug Administration 17
13.4. Packaging 17

13.5. Preparation and Labeling ... 17
13.6. Storage ... 17
13.7. Drug Accountability ... 18

14. Data Collection ... 18

14.1. Case Report Forms (CRFs) ... 18

15. Statistical Section ... 19

15.1. Introduction ... 19
15.2. Study Design ... 19
15.3. Study Endpoints ... 19
15.4. Sample Size Justification ... 20
15.5. Primary Efficacy Analyses ... 20
15.6. Secondary Analyses ... 20
15.7. Study Populations ... 20
15.8. Interim Safety Analysis ... 21
15.9. Safety Analysis ... 21
15.10. Pharmacokinetic Analysis ... 21

16. Safety Measurements ... 21

16.1. Patients' Monitoring ... 21
16.2. Laboratory Safety Studies ... 21
16.3. Adverse Events ... 21
16.4. Serious Adverse Events ... 22
16.5. Pregnancy ... 23

17. Concomitant Medication ... 23

18. Discontinuation from Study ... 23

19. Study Records ... 23

20. Reporting of Results ... 24

21. Study Conduct ... 25

22. REFERENCES ... 26

23. Appendices ... 28

23.1. Appendix 1. Study Flow Chart ... 29
23.2. Appendix 2. MRI ... 30
23.3. Appendix 3. Vial Label ... 32

Abbreviations:

AE	Adverse event
CBC	Complete blood count
CRF	Case Report Form
CTM	Clinical test material
DEXA	Dual-energy x-ray absorptiometry
DNA	Deoxyribonucleic acid
ECHO	Echocardiogram
ERT	Enzyme replacement therapy
ESR	Erythrocyte sedimentation rate
FDA	Food and Drug Administration
GCD	Glucocerebrosidase
GCP	Good Clinical Practice
ICF	Informed consent form
ICH	International Conference on Harmonization
IRB	Institutional Review Board
MedDRA	Medical Dictionary for Regulatory Authorities
MRI	Magnetic Resonance Imaging
PARC/CCL 18	Chemokine (C-C motif) ligand 18 (pulmonary and activation-regulated)
PI	Principal Investigator
PK	Pharmacokinetic
prGCD	Plant cell expressed recombinant human glucocerebrosidase
QCSI	Quantitative Chemical Shift Imaging
SRT	Substrate reduction therapy

2. Introduction

Gaucher disease, the most prevalent lysosomal storage disorder (1,2), is caused by mutations in the human glucocerebrosidase gene (GCD), which had been mapped to chromosome 1 q21-q31, leading to reduced activity of the lysosomal enzyme glucocerebrosidase and thereby to the accumulation of substrate glucocerebroside (GlcCer) in the cells of the monocyte-macrophage system. This accumulation leads to the visceral manifestations of hepatosplenomegaly, anemia and thrombocytopenia, as well as to the skeletal features and less frequently also to lung involvement (3). There are three clinical types of Gaucher disease, according to the existence and severity of neuronopathic involvement. Type 1 is non-neuronopathic whereas Types 2 and 3 are acute and subacute neuronopathic, respectively.

The gene encoding human GCD was first sequenced in 1985 (4) The protein consists of 497 amino acids derived from a 536-mer pro-peptide. The mature hGCD contains five N-glycosylation amino acid consensus sequences (Asn-X-Ser/Thr). Four of these sites are normally glycosylated. Glycosylation of the first site is essential for the production of active protein. Both high-mannose and complex oligosaccharide chains have been identified (5). hGCD from placenta contains 7% carbohydrate, 20% of which is of the high-mannose type (6). Biochemical and site-directed mutagenesis studies have provided an initial map of regions and residues important to folding, activator interaction, and active site location (7).

The identification of GCD deficiency as the etiology of Gaucher disease stimulated the development of enzyme replacement therapy (ERT) as a therapeutic strategy for this disorder, which has been proven safe and effective over the past 14 years, in over 4000 patients worldwide using either natural (placental-derived) or recombinant human glucocerebrosidase derived from mammalian tissue culture production systems. One of the major disadvantages with this treatment is its high cost, which approximately cost 250,000 dollars per patient per year (8-11). Because the enzyme cannot cross the blood-brain-barrier, most of the patients who have been treated with ERT were those with type I Gaucher disease, although patients with type III also benefit from the effects of ERT on the visceral and skeletal manifestations. Similar results have been reported with both the placental derived enzyme (alglucerase; Ceredase™) which was approved by the FDA in 1991, and with the human recombinant enzyme (imiglucerase; Cerezyme™; both produced by Genzyme Therapeutics USA) which has been commercially available since 1994 (11).

Studies have shown that glycosylation plays a crucial role in glucocerebrosidase activity and uptake to target cells, and indeed, in the formulations currently used special steps of deglycosylation are required in order to generate exposed mannose residues as the terminal glycoside side chains (Bijsterbosch MK. et al. 1996; Friedman B. et al. 1999; Furbish FS. et al. 1981; Doebber T. et al. 1982).

Unmodified hGCD, derived from natural sources, cannot be targeted to the phagocytic cells in the body and is, therefore, of limited therapeutic value. In developing the current therapeutic products for Gaucher disease, the terminal sugars on the carbohydrate chains of hGCD are sequentially removed by treatment with three different glycosidases. This glycosidase treatment results in a glycoprotein whose terminal sugars consist of mannose residues. This facilitates uptake by mannose receptors on phagocyte cells that recognize glycoproteins and glycopeptides with

oligosaccharide chains that terminate in mannose residues. The carbohydrate remodeling of hGCD improves the targeting of the enzyme to these cells (14-17).

Since the glycosylation pattern of hGCD must be remodeled to generate high mannose structures to increase uptake in target cells, the expression of hGCD in plant cells could be of great value. Post-translational modifications do not exist in bacterial expression systems, but plant derived expression systems do facilitate these modifications known to be crucial for protein expression and activity. One of the major differences between mammalian and plant protein expression systems is the variation of protein sugar side chains, caused by the differences in biosynthetic pathways. Glycosylation has been shown to have a profound effect on activity, folding, stability, solubility, and susceptibility to proteases, blood clearance rate and antigenic potential of proteins. Hence, any protein production in plants should take into consideration the potential ramifications of plant glycosylation.

The production of prGCD utilizes Agrobacterium tumefacience, a bacterium capable of inserting single stranded DNA molecules (T-DNA) into the plant genome. Due to the relative simplicity of introducing genes for mass production of proteins and peptides, this methodology is becoming increasingly popular as an alternative protein expression system (1).

3. Objectives

The objective of this study is to assess the safety and efficacy of prGCD in patients with significant signs and symptoms of Gaucher disease not treated with enzyme replacement therapy.

3.1. Primary Efficacy Endpoint

The primary efficacy endpoint will be change from baseline in spleen volume at 9 months.

3.2. Secondary Endpoints

Secondary efficacy endpoints are change from baseline in:

- Liver volume
- Platelet count
- Hemoglobin level
- Biomarkers (chitotriosidase and pulmonary and activation-regulated chemokine (PARC/CCL18)
- Proportion of patients with greater than 10% reduction in spleen volume at 9 months

3.3. Safety Endpoints

Safety will be assessed by adverse events, physical examination (changes in vital signs and body weight), concomitant medications and laboratory test results:

- Hematology: erythrocyte sedimentation rate (ESR), complete blood count; [total white blood cell count, differential count (neutrophils, lymphocytes, monocytes, eosinophils and basophils), red blood cells, hemoglobin, hematocrit, mean corpuscular volume, mean

corpuscular hemoglobin and mean corpuscular hemoglobin concentration], platelet count, and coagulation profile (prothrombin time, partial thromboplastin time)
- Biochemistry: sodium, potassium, glucose, hemoglobin A1c, blood urea nitrogen, creatinine, calcium, phosphate (inorganic), uric acid, total protein, albumin, bilirubin (total), alkaline phosphatase, aspartate transaminase, alanine transaminase, gamma-glutamyl transferase, lactate dehydrogenase, serum iron transferin, ferritin, vitamin B_{12}, folic acid, protein electrophoresis and immunoglobulin profile.
- Urinalysis: Dipstick for presence of glucose, ketones and protein
- Electrocardiogram
- Echocardiogram
- Pulmonary function tests

3.4. Additional Exploratory outcomes measurements

- Quantitative Chemical Shift Imaging (QCSI) for a subgroup of patients
- Change in bone mineral density by DEXA (dual-energy x-ray absorptiometry)

3.5. Pharmacokinetics

Pharmacokinetics of prGCD will be assessed in all patients after the first and last doses of prGCD.

4. Principal Investigator

The Principal Investigator (PI) at each center has the responsibility for the conduct and compliance of this clinical trial according to this protocol and Good Clinical Practices (GCP).

5. Institutional Review Board

This protocol and the Informed Consent must be reviewed and approved by the appropriate Institutional Review Board (IRB) associated with the study site. Any additional protocol amendments must be approved by the IRB prior to their implementation. A copy of the letter, signed by either the Chairman of the IRB or the Director General of the hospital (country dependent), to the Principal Investigator indicating IRB approval of the protocol must be received by the sponsor and maintained in the study file prior to study initiation. Drug supply will not be shipped to the study site until the sponsor receives this documentation.

6. Informed Consent

The risks and benefits of participating in this study will be explained to each candidate patient prior to entering into the study. The informed consent will be written in language readily understood by the patient. The informed consent must be approved by the IRB prior to study initiation, performance of any study procedure and dispensing of the study drug. The Principal Investigator or his/her designee must obtain a signed and witnessed Informed Consent Form for each patient. Receipt of the signed Informed Consent Form will be documented in the Case Report Form and a copy retained by the Investigator. A copy of the signed Informed Consent Form will be given to each patient.

7. Patient Population, Number of Patients and Study Centers

Thirty (30) untreated patients aged 18 years and older who have signs and symptoms of Gaucher disease will be enrolled from 10-20 centers.

8. Treatment Groups

Thirty (30) eligible patients will be randomized and allocated to one of the two treatment groups, 15 patients each to receive either 30 units/kg or 60 units/kg of study drug (prGCD) bi-weekly.

Treatment Group I: 30 units/kg every 2 weeks.
Treatment Group II: 60 units/kg every 2 weeks.

This will be a double-blinded study; neither the patient nor their physician will know the dosing regimen. Only the pharmacist providing the clinical test material (CTM) will know the dosing regimen.

The final concentration of prGCD is 40 units/mL after reconstitution with water for injection. A unit is defined as the amount of enzyme that catalyzes the hydrolysis of 1 micromole of the synthetic substrate para-nitrophenyl-beta-D-glucopyranoside (pNP-Glc) per minute at 37^0C. The actual dose administered will be rounded to multiples of the number of units in each vial to mimic the use of the enzyme in real life, and to avoid wastage of enzyme.

9. Randomization

A unique screening number (formatted as xx-S-yyy [x: site number, y: patient number]) will be assigned to screened patients. Once a patient is eligible for randomization, after successful completion of screening and prior to visit 1, a patient randomization number will be generated that will include the site and patient number (formatted as xx-yyy [x: site number, y: randomization number]). Patients will be randomized to the two treatment groups (30 units/kg or 60 units/kg) based on a computer-generated randomization code.

10. Patient Selection

Potential patients identified by the principal investigator at each site will be screened to determine their eligibility according to the following inclusion and exclusion criteria. Once a patient has been found to be eligible, the Coordinating Investigator and the Sponsor's Medical Director will review all patient screening data and provide final approval for randomization.

10.1. Inclusion Criteria

Patients must meet all of the following criteria in order to be eligible to enter the study:

1. Males and females, 18 years or older
2. Confirmed enzymatic diagnosis of Gaucher disease
3. Splenomegaly defined as greater than eight times the expected volume [measured volume divided by estimated volume (0.2% of body weight)] as determined by MRI volumetric analysis

4. Female patients of child-bearing potential who agree to use a medically acceptable method of contraception, not including the rhythm method.
5. Thrombocytopenia (defined as platelet counts below the lower limit of normal) and/or anemia (defined by hemoglobin level at least 1 g/dL below normal range according to sex and age).
6. Patients who have not received ERT in the past or Patients who have not received ERT in the past 12 months and have a negative anti-glucocerebrosidase antibody test.
7. Patients who have not received substrate reduction therapy (SRT) in the past 12 months.
8. Ability to provide a written informed consent.

10.2. Exclusion Criteria

Patients must be excluded if they meet any of the following criteria:

1. Currently taking another experimental drug for any condition
2. Pregnant or nursing
3. Presence of HIV and/or, HBsAg, and/or hepatitis C infections
4. Presence of any medical, emotional, behavioral or psychological condition that in the judgment of the Investigator would interfere with the patient's compliance with the requirements of the study.

11. Study Design

See Appendix 1 for the Study Flow Chart.

This will be a multi-center, randomized, double-blind, parallel group, dose-ranging trial to assess the safety and efficacy of prGCD in 30 untreated patients with Gaucher disease. Patients will receive IV infusion of prGCD every two weeks at the selected medical center. The duration of the study will be nine months. At the end of the 9-month treatment period (20 visits, 38 weeks) eligible patients will be offered enrollment in an open-label extension study.

There will be two treatment groups, 15 patients in each treatment group.

Treatment Group I: 30 units/kg every 2 weeks.
Treatment Group II: 60 units/kg every 2 weeks.

All patients will have pharmacokinetic data collected over a 210 minute period with frequent blood samples following the first and final doses of prGCD.

All MRI volumetric analyses will be analyzed by a central expert reader blinded to the randomized treatment group. The MRI systems at each center will be calibrated and standardized following a standard protocol (Appendix 23.2).

Adverse events, concomitant medications and body weight will be recorded at each visit. Efficacy parameters (spleen and liver volumes, hemoglobin, platelets, biomarkers and antibody analysis) will be collected at the following visits: screening, baseline (Visit 1), Month 6 (Visit 14) and Month 9 (Visit 20). At the Month 3 (visit 7), only hematology, biochemistry, biomarker and safety data will be collected. Complete blood count (CBC), blood chemistry and urinalysis will be performed at Weeks

2, 4, 6, and 8 and then at every other visit (once a month) for 9 months. Blood pregnancy test will be performed at screening, baseline (Visit 1), Month 6 (Visit 14) and Month 9 (Visit 20)

12. Study Visits

12.1. Screening (Visit 0, Day -21 ± 7)

1. Obtain written informed consent from the patient
2. Assign screening number
3. Demographics
4. Medical history
5. Physical examination, including body weight
6. MRI for spleen and liver volume
7. Blood platelet count including blood smear and hemoglobin
8. Current medications
9. Clinical laboratory tests (chemistry, hematology, urinalysis)
10. Glucocerebrosidase activity (may be omitted at the Medical Director's discretion)
11. GCD antibody test, if the patient was exposed to ERT prior to enrollment
12. Blood pregnancy test- beta-HCG
13. Serology for HIV, HBsAg, HCV
14. Echocardiography
15. Chest X ray
16. Pulmonary function tests
17. Bone mineral densitometry (DEXA)
18. Review all inclusion and exclusion criteria and determine subject's eligibility

19. If a patient qualifies for the study, perform the optional Quantitative Chemical Shift Imaging (QCSI) assessment of bone marrow fat content in lumbar spine prior to Visit 1. This test will be performed in a subpopulation of patients at selected centers.

12.2. Visit 1 (Baseline, Day 1)

1. Assign to randomized treatment group
2. Concomitant medications
3. Physical examination (including vital signs and body weight)
4. Clinical laboratory tests
   - Hematology
   - Biochemistry
   - Urinalysis
5. Pregnancy test, Beta-HCG.
6. Antibody analysis: Serum samples for anti prGCD antibody
7. Biomarkers: chitotriosidase and PARC/CCL18
8. Electrocardiogram
9. Skeletal evaluation: full x-ray series if not available within 6 months prior to enrollment in the study; lumbar spine (lateral only), pelvis, femora and humeri
10. prGCD infusion

11. Blood samples for pharmacokinetic analysis at 0, 5, 45, 70, 80, 95, 110, 125, 150 and 175 after the start of the infusion. ,

The following procedures will be performed after prGCD dosing:

- The patients will be observed clinically for a minimum of 2 hours
- Vital signs will be evaluated every 30 minutes
- The injection site will be evaluated

The patient should be reminded of the date of their next visit.

12.3. Visits 2 – 6 (Weeks 2, 4, 6, 8, 10 ± 3 Days)

Patients will receive their prGCD infusion at the selected medical center. The following procedures will be performed after dosing:

- The patients will be observed clinically for a minimum of 2 hours after dosing.
- Vital signs will be evaluated every 30 minutes
- The injection site will be evaluated.

Adverse events and concomitant medications will be recorded at each visit.

Laboratory tests (hematology, biochemistry and urinalysis) will be performed at visits 2, 3, 4 and 5

The patient should be reminded of the date of their next visit.

12.4. Visit 7 (Month 3, Week 12 ± 7 Days)

The following assessments will be performed:

1. Adverse events and concomitant medications
2. Physical examination including vital signs and body weight
3. Blood samples for:
   - Hematology
   - Biochemistry
   - Biomarkers: chitotriosidase and PARC/CCL18
4. Urine analysis
5. Blood pregnancy test, Beta-HCG.
6. Electrocardiogram
7. prGCD infusion

The following procedures will be performed after dosing:

- The patients will be observed clinically for 2 hour after dosing.
- Vital signs will be evaluated every 30 minutes
- The injection site will be evaluated.

The patient should be reminded of the date of their next visit.

12.5. Visits 8 – 13 (Weeks 14, 16, 18, 20, 22, 24 ± 3 days)

Patients will receive their prGCD infusion at the selected medical center. The following procedures will be performed after dosing:

- The patients will be observed clinically for 2 hour after dosing.
- Vital signs will be evaluated every 30 minutes
- The injection site will be evaluated.

Safety data (adverse events and concomitant medications) will be recorded at each visit.

Laboratory tests (hematology, biochemistry and urinalysis) will be performed at Visits 9, 11, and 13.

The patient should be reminded of the date of their next visit.

12.6. Visit 14 (Month 6, Week 26 ± 7 days)

1. Organ volumes: MRI (volumetric assessment of liver and spleen) according to protocol for organs' volume evaluation, appendix 22.2
2. Adverse events and concomitant medications
3. Physical examination including vital signs and body weight
4. Blood samples for:
   - Hematology
   - Biochemistry
   - Biomarkers: chitotriosidase and PARC/CCL18 (PARC).
5. Blood pregnancy test, Beta-HCG
6. Urine analysis
7. Electrocardiogram
8. prGCD infusion

The following procedures will be performed after dosing:

- The patients will be observed clinically for 2 hour after dosing.
- Vital signs will be evaluated every 30 minutes
- The injection site will be evaluated.

The patient should be reminded of the date of their next visit.

12.7. Visit 15 – 19 (Weeks 28, 30, 32, 34, 36 ± 3 days)

Patients will receive their prGCD infusion at the selected medical center. The following procedures will be performed after dosing:

- The patients will be observed clinically for 2 hour after dosing.
- Vital signs will be evaluated every 30 minutes

- The injection site will be evaluated.

Safety data (adverse events and concomitant medications) will be recorded at each visit.

Laboratory tests (hematology, blood chemistry and urinalysis) will be performed at visits16 and 18.

12.8. Visit 20 (Month 9, Week 38 ± 7 days)

The following assessments will be performed:

1. Adverse events and concomitant medications
2. Physical examination and weight.
3. Electrocardiogram
4. Chest X ray
5. Echocardiography
6. Pulmonary Function tests
7. Organ volumes: MRI (volumetric assessment of liver and spleen) according to protocol for organs' volume evaluation, appendix 22.2
8. Bone Mineral Densitometry (DEXA)
9. QCSI for those patients who were assessed at baseline.
10. Anti human prGCD antibodies
11. Clinical laboratory tests
    - Hematology
    - Biochemistry
    - Urinalysis
12. Blood pregnancy test, Beta-HCG
13. Biomarkers (Chitotriosidase and PARC/CCL18)

prGCD infusion

14. Blood samples for pharmacokinetic analysis at 0, 5, 45, 70, 80, 95, 110, 125, 150 and 175 after the start of the infusion. ,

The following procedures will be performed after prGCD dosing:

- The patients will be observed clinically for a minimum of 2 hours after dosing
- Vital signs will be evaluated every 30 minutes
- The injection site will be evaluated

13. Study Medication

13.1. Dosage

Two treatment groups will be evaluated in this study:

- prGCD 30 units/kg body weight

- prGCD 60 units/kg body weight

A unit is defined as the amount of enzyme that catalyzes the hydrolysis of 1 micromole of the synthetic substrate para-nitrophenyl-beta-D-glucopyranoside (pNP-Glc) per minute at 37°C. Human prGCD vials are stored lyophilized at 4°C. The human prGCD final concentration is 40 units/ml after reconstitution with WFI.

The individual dose for each subject will be prepared according to subject weight and randomized dose and for practical reasons the dose in terms of total units will be rounded up to avoid use of partial vials, e.g., a 65 kg subject assigned to 30 units/kg dose will receive 2000 units (10 vials) whereas the calculated dose is 1950 units. Each dose will be prepared by an unblinded pharmacist at each site.

13.2. Formulation

Human prGCD is a purified recombinant, plant cells-expressed glucocerebrosidase, which is described in detail in the Investigator Brochure.

Each vial contains the following lyophilized contents:
200U of prGCD
195 mg mannitol
35 mg sodium citrate
0.53 mg polysorbate 80, NF

13.3. Study Drug Administration

Human prGCD will be administered as an intravenous infusion (IV) delivered over 1 hour. The infusion rate will be constant over 60 minutes for all doses. The infusion rate may be adjusted according to subject symptoms and signs

13.4. Packaging

Lyophilized drug powder is stored in 10 ml low extractable borosilicate glass bottles (Bunder Glass, Germany). Lyophilization stoppers (Stelmi, France) composed of two-leg grey butyl rubber are sealed with Tear-Cap® Aluminum seals (Wheaton Science Product, USA).

13.5. Preparation and Labeling

Reconstitution with 5.1mL of Sterile Water for Injection (WFI) yields a final volume of 5.3 mL human prGCD (40 units/mL), which provides a withdrawal volume of 5.0 mL (200 enzyme units). The solution must be mixed gently until clear. The required amount of enzyme units will be adjusted with normal saline (0.9%) up to 135ml/infusion.

13.6. Storage

The product is stored at 2-8°C (36-46°F).

13.7. Drug Accountability

Protalix will provide drug accountability forms to assist the investigator in maintaining current and accurate inventory records covering receipt, dispensing, and the return of investigational drug supplies. When a shipment is received, the investigator or pharmacist will verify the quantities received and return the acknowledgment to the study monitor or designee. The investigational drug accountability record includes the study identification of the person to whom the drug is dispensed, the quantity and the date of dispensing and any returned or unused drug. This record is in addition to any drug accountability information recorded on the Case Report Form (CRF). These records will be readily available for inspection by a monitor or Protalix audits and are open to regulatory authority inspection at any time.

14. Data Collection

The Case Report Form (eCRF) is an integral part of the study and subsequent reports. The CRF must be used to capture all study data recorded in the patient's medical record. The CRF must be kept current to reflect patient status during the course of the study. Only a patient screening and randomization number and patient initials will be used to identify the patient.

The monitor is responsible for performing on-site monitoring at regular intervals throughout the study to verify adherence to the protocol; verify adherence to local regulations on the conduct of clinical research; and ensure completeness, accuracy, and consistency of the data entered in the CRF.

14.1. Case Report Forms (CRFs)

Target Health Inc. will monitor completed Case Report Forms (CRFs). A case report form will be provided for each screened patient.

All protocol-required information collected during the study must be entered by the Investigator, or designated representative, in the Target e*CRF™, an Internet-based data collection system (eCRF). All details of the CRF completion and correction will be explained to the investigator. The management module of Target e*CRF™, includes edit check and query systems that seamlessly integrate with the data entry system. All modifications to the data in the eCRF are tracked by an electronic audit trail (date and identity of the person making the change are instantaneously recorded). Target e*CRF™ is 21CFR Part 11 compliant.

If the Investigator authorizes other persons to make entries in the CRF, the names, positions, and signatures of these persons must be supplied to the sponsor.

The Investigator, or designated representative, should complete the eCRF as soon as possible after information is collected, preferably on the same day that a study patient is seen for an examination, treatment, or any other study procedure. Any outstanding entries must be completed immediately after the final examination. By design, an explanation must be provided for all missing data and/or out of range data.

The completed case report form must be reviewed and signed by the Investigator named in the study protocol or by a designated sub investigator.

Final monitored and audited eCRFs will be provided by the Sponsor to the sites at the end of the study in the format of a PDF file.

15. Statistical Section

16. Safety Measurements

16.1. Patients' Monitoring

Patients will be monitored at all visits, to determine whether or not any adverse events have occurred.

16.2. Laboratory Safety Studies

All laboratory measurements will be displayed for each visit, and compared to the baseline measurements.

16.3. Adverse Events

An adverse event is any undesirable, unintentional or unexpected (unanticipated) event that occurs throughout the study, whether or not considered related to the drug. Adverse events will be monitored throughout the study, and recorded in the eCRF. The need to capture this information is not dependent upon whether the adverse event is associated with the use of the study drug. Adverse events resulting from concurrent illnesses or reactions to concurrent medications are also to be recorded. In order to avoid vague expressions, the adverse event will be recorded in standard medical terminology.

DEGREE OF INTENSITY	DESCRIPTION
Mild	Awareness of signs and symptoms; easily tolerated
Moderate	Discomfort sufficient to interfere, but not prevent, daily activity
Severe	Unable to carry out usual activity

Action taken - whether or not the adverse event caused the patient to be withdrawn from the study

Relationship - whether or not the test product caused the adverse event

DEGREE	DESCRIPTION
Definitely	There is evidence of exposure to the test product, for example, reliable history or acceptable compliance assessment; the temporal sequence of the AE onset relative to the drug is reasonable; the AE is most likely to be explained by the drug treatment than by another cause; the challenge is positive; re-challenge (if feasible) is positive; the AE shows a pattern consistent with previous knowledge of the drug treatment.
Probably	There is evidence of exposure to the test product; the temporal sequence of the AE onset relative to the drug administration is reasonable; the AE is more likely explained by the drug treatment than by another cause; the challenge (if performed) is positive.
Possibly	There is evidence of exposure to the test product; the temporal sequence of the AE relative to the drug administration is reasonable; the AE could have been due to another equally likely cause; the challenge (if performed) is positive.
Probably not	There is evidence of exposure to the drug; there is another more likely cause of the AE; the challenge (if performed) is negative or ambiguous; rechallenge (if performed) is negative or ambiguous.
Definitely not	The patient/patient did not receive the drug treatment; or temporal sequence of the AE onset relative to administration of the drug is not reasonable; or there is another obvious cause of the AE.

Severity of the AE

DEGREE	DESCRIPTION
Mild	Symptom(s) barely noticeable to patient or does not make patient uncomfortable; does not influence performance or functioning; prescription drug not ordinarily needed for relief of symptom(s) but may be given because of personality of patient.
Moderate	Symptom(s) of a sufficient severity to make patient uncomfortable; performance of daily activity is influenced; patient is able to continue in study; treatment for symptom(s) may be needed.
Severe	Cause severe discomfort; symptoms cause incapacitation or significant impact on patient’s daily life; severity may cause cessation of treatment with study device; treatment for symptom(s) may be given and/or patient hospitalized.

16.4. Serious Adverse Events

A serious adverse event means any event that suggests a significant hazard, contraindication, side effect, or precaution. With respect to human clinical events, a serious adverse event includes any event that is fatal or life threatening requires hospitalization or extends the hospitalization of hospitalized patients, results in a persistent or significant disability/incapacity, results in a congenital anomaly, cancer, or overdose.

The Investigator shall, within 24 hours of occurrence or notification of an SAE, report the SAE to the Medical Monitor and/or the Clinical Research Associate, who will then inform the Sponsor of the serious adverse event. SAEs may need to be reported to all IECs/IRBs according to local requirements and applicable health authorities in accordance with applicable regulatory requirements.

The sponsor shall notify the appropriate regulatory authorities as required and all participating Investigators of any adverse event associated with use of the drug that is both serious and unexpected. The Investigator must also notify the Institutional Review Board.

16.5. Pregnancy

Although pregnancy as such is not considered an AE or SAE, it is the responsibility of the Investigator to report to Protalix, by telephone immediately, any pregnancy occurring in a female study subject either during the study or within 42 days following the last dose of study drug. Protalix will provide the Investigator with a Pregnancy Tracking Form that is to be completed by the study site on a periodic basis and faxed to Protalix. The Investigator will follow the pregnancy until the end of the pregnancy. If the pregnancy continues to term (delivery), the health of the infant must also be reported to Protalix.

17. Concomitant Medication

Medications will be recorded in the CRF.

18. Discontinuation from Study

Patients will be discontinued from treatment with study drug if:

- The patient requests to discontinue treatment
- Investigator feels that it is not in the best interest of the patient to continue treatment and/or if the investigator believes that the patient can no longer be compliant with the requirements of the study.
- Pregnancy

For any discontinuation, the Investigator will obtain all the required details and document the date and the main reason for the premature termination. If the reason for discontinuation is an adverse event, the specific event or the main laboratory abnormality will be recorded in the CRF. The Investigator will make thorough efforts to document the outcome. The Investigator will attempt to continue to follow the patient for the full duration of the study or at least for 30 days following discontinuation. If circumstances prevent the patient from completing all visits, every attempt will be made to complete all procedures listed in Section 11.8 for Visit 20.

19. Study Records

Study records including case report forms (CRFs), patient progress notes, original copies of test results, signed informed consent forms, a patient enrollment log, a drug dispensation log, Institutional Review Committee approval letters, and other documents pertaining to the conduct of the study must be kept on file by the Investigator. Study records are to be available for sponsor inspection at any time.

All study records will be retained for at least the shortest of a period of 2 years following the date on which the drug is approved by the regulatory authority for marketing for the purposes that were the patient of the investigation. In other situations (e.g., where the investigation is not in support of or

as part of an application for a research or marketing permit), a period of 2 years following the date on which the entire clinical program is completed, terminated or discontinued or the investigational application under which the investigation is being conducted is terminated or withdrawn by the regulatory authorities.

In the event the Investigator retires, relocates or for any other reason withdraws from the responsibility for maintaining records for the period of time required, custody of the records may be transferred to any other person who will accept responsibility for the records. Notice of such a transfer must be given in writing to the Sponsor. The Investigator must contact the Sponsor prior to disposal of any records related to this study.

20. Reporting of Results

The Investigator will record all drug administration data, results of laboratory tests, side effects and efficacy data on the Case Report Form (CRF). Photocopies of original laboratory slips or computer printout of the relevant data must be available for inspection by the sponsor upon request.

21. Study Conduct

THIS STUDY WILL BE CONDUCTED IN ACCORDANCE WITH GOOD CLINICAL PRACTICE REQUIREMENTS.

APPROVED ____
(Investigator’s Signature)

DATE APPROVED: ___________________________________

APPROVED ____
(Sponsor’s Signature)

Title __VP Product Development_____________

DATE APPROVED: ___________________________________

22. REFERENCES

1. Lee, RE. The pathology of Gaucher disease. Prog Clin Biol Res 1982;95:177-217.
2. Grabowski G. Gaucher disease: enzymology, genetics, and treatment. Adv Hum Genet. 1993;21:377-441.
3. Grabowski GA, Hopkin RJ. Enzyme Therapy for Lysosomal Storage Disease: Principles, Practice, and Prospects. Annual Review of Genomics and Human Genetics 2003;4:403-436.
4. Sorge JWC, Westwood B, Beutler E. Molecular Cloning and Nucleotide Sequence of Human Glucocerebrosidase cDNA. Proc Natl Acad Sci USA. 1985;82:7289-7293.
5. Berg-Fussman A, Grace M, Ioannou Y, and Grabowski G. Human acid beta-glucosidase. N-glycosylation site occupancy and the effect of glycosylation on enzymatic activity. J. Biol. Chem. 1993;268:14861-14866.
6. Grace M, Grabowski GA. Human acid ß-glucosidase: glycosylation is required for catalytic activity. Biochem Biophys Res Commun 1990;168:771-777.
7. Grace M, Newman K, Scheinker V, Berg-Fussman A, and Grabowski G. Analysis of human acid beta-glucosidase by site-directed mutagenesis and heterologous expression. J. Biol. Chem. 1994;269:2283-2291.
8. Barton NW, Brady RO, Dambrosia JM, Di Bisceglie AM, Doppelt SH, Hill SC, Mankin HJ, Murray GJ, Parker RI, Argoff CE, et al. Replacement therapy for inherited enzyme deficiency--macrophage-targeted glucocerebrosidase for Gaucher's disease. N Engl J Med. 1991;324:1464-1470.
9. Grabowski GA, Barton NW, Pastores G, Dambrosia JM, Banerjee TK, McKee MA, Parker C, Schiffmann R, Hill SC, and Brady RO. Enzyme Therapy in Type I Gaucher disease: Comparative Efficacy of Mannose-terminated Glucocerebroidase from Natural and Recombinant Sources. Ann Intern Med 1995;122:33-39.
10. Pastores GM, Sibille AR, Grabowski GA. Enzyme therapy in Gaucher disease type 1: dosage efficacy and adverse effects in 33 patients treated for 6 to 24 months. Blood. 1993;82:408-416.
11. Weinreb NJ, Charrow J, Andersson HC, Kaplan P, Kolodny EH, Mistry P, Pastores G, Rosenbloom BE, Scot, CR, Wappner RS, Zimran A. Effectiveness of enzyme replacement therapy in 1028 patients with type 1 Gaucher disease after 2 to 5 years of treatment: a report from the Gaucher Registry. Am J Med. 2002;113:112-119.
12. Ma JKC, Drake PMW, and Christou P. The production of recombinant pharmaceutical proteins in plants. Nature reviews Genetics. 2003;4:794-805.
13. Lerouge P, Cabanes-Macheteau M, Rayon C, Fischette-Laine AC, Gomord V, Faye L. N-glycoprotein biosynthesis in plants: recent developments and future trends. Plant Mol Biol. 1998;38:31-48.
14. Anand G. Uncertain miracle: A biotech drug extends a life, but at what price? The Wall Street Journal November 16, 2005; Page A1.
15. Bijsterbosch MK, Donker W, van de Bilt H, van Weely S, van Berkel TJ, Aerts JM. Quantitative analysis of the targeting of mannose-terminal glucocerebrosidase. Predominant uptake by liver endothelial cells. Eur J Biochem 1996;237:344-349.
16. Friedman B, Vaddi K, Preston C, Mahon E, Cataldo JR and McPherson JM. A Comparison of the Pharmacological Properties of Carbohydrate Remodeled Recombinant and Placental-Derived beta -Glucocerebrosidase: Implications for Clinical Efficacy in Treatment of Gaucher Disease. Blood 1999;93:2807-2816.

17. Furbish FS, Steer CJ, Krett NL, Barranger JA. Uptake and distribution of placental glucocerebrosidase in rat hepatic cells and effects of sequential deglycosylation. Biochim Biophys Acta. 1981;673:425-434.
18. Doebber T, Wu M, Bugianesi R, Ponpipom M, Furbish F, Barranger J, Brady R, and Shen T. Enhanced macrophage uptake of synthetically glycosylated human placental beta-glucocerebrosidase. J. Biol. Chem. 1982;257:2193-2199.
19. Dwek RA, Butters, TD, Platt FM, Zitzmann N. Targeting Glycosylation as a Therapeutic Approach. Nature reviews 2002;1:65-75.
20. Neuhaus JM, Rogers JC, Sorting of proteins to vacuoles in plant cells. Plant Mol Biol. 1998;38:127-144.
21. Vitale A, and Galili G. The Endomembrane System and the Problem of Protein Sorting. Plant Physiol. 2001;125:115-118.
22. Clarke JT, Amato D, Deber RB. Managing public payment for high-cost, high-benefit treatment: enzyme replacement therapy for Gaucher's disease in Ontario. CMAJ. 2001 Sep 4;165(5):595-6.
23. Kannus P, Khan KM. Prevention of falls and subsequent injuries in elderly people: A long way to go in both research and practice. CMAJ September 4 2001;165(5):587-8.
24. Zimran A, Elstein D, Kannai R, Zevin S, Hadas-Halpern I, Levy-Lahad E, Cohen Y, Horowitz M, Abrahamov A. Low-dose enzyme replacement therapy for Gaucher's disease: effects of age, sex, genotype, and clinical features on response to treatment. Am J Med. 1994;97(1):3-13.
25. Zimran A, Elstein D, Levy-Lahad E, Zevin S, Hadas-Halpern I, Bar-Ziv Y,Foldes J, Schwartz AJ, Abrahamov A.Replacement therapy with imiglucerase for type 1 Gaucher's disease.Lancet. 1995;345(8963):1479-80.
26. Gillis S, Hyam E, Abrahamov A, Elstein D, Zimran A. Platelet function abnormalities in Gaucher disease patients. Am J Hematol. 1999;61(2):103-6.
27. Elstein D, Abrahamov A, Hadas-Halpern I, Zimran A. Gaucher's disease. Lancet. 2001;358(9278):324-7.

23. Appendices

23.1. Appendix 1. Study Flow Chart

Activity	Visit 0 Screening	Visit 1 Baseline	Visits 2-6	Visit 7 Month 3	Visits 8-13	Visit 14 Month 6	Visits 15-19	Visit 20 Month 9
	Day -21±7	Day 1[1]	Weeks 2[1], 4[1], 6[1], 8[1], 10	Week 12[1]	Weeks 14, 16[1], 18, 20[1], 22, 24[1]	Week 26[1]	Weeks 28, 30[1], 32, 34[1], 36	Week 38[1]
Sign IC	X							
Review Inclusion/Exclusion Criteria	X							
Medical History	X							
Adverse Events		X	X	X	X	X	X	X
Current/Concomitant Medications	X	X	X	X	X	X	X	X
Weight	X	X		X		X		X
Physical Examination	X	X		X		X		X
Electrocardiograph (ECG)		X		X		X		X
Chest X-Ray	X							X
X-RAY Skeketal Evaluation		X						
Echocardiography	X							X
Pulmonary Function Tests (PFT)	X							X
Organ Volumes (MRI)	X					X		X
Bone Mineral Densitometry (DEXA)	X							X
QCSI[2]	X							X
Anti Human prGCD Antibodies	X[3]	X		X		X		X
Hematology	X	X[1]	X[1]	X[1]	X[1]	X[1]	X[1]	X[1]
Biochemistry	X	X[1]	X[1]	X[1]	X[1]	X[1]	X[1]	X[1]
Serology	X							
Beta HCG	X			X		X		X
Urinalysis	X	X[1]	X[1]	X[1]	X[1]	X[1]	X[1]	X[1]
Glucocerebrosidase activity[4]	X							
Biomarker		X		X		X		X
IV Infusion (ERT)		X	X	X	X	X	X	X
PK Serum prGCD Protein Level		X						X

[1]Hematology, biochemistry and urinalysis will be performed at every visit in the first 5 visits, and following that every other visit. In addition, visit 7, 14, and 20 will include these tests.
[2]QCSI will be performed in a subpopulation of patients at selected centers after Screening but before Visit 1 and Visit 20
[3]Only for patients who have been exposed to glucocerebrosidase before the trial
[4]May be omitted at the Medical Director's discretion

23.2. Appendix 2. MRI

Evaluation of Spleen and Liver Volume using MRI

1. Introduction:
Reduction in spleen volume is the primary outcome of the Protocol Number: PB-06-001 that will establish the efficacy of the treatment. Liver volume is an additional clinical parameter in the study and reduction in liver volume is a secondary outcome of the study along with increase in platelet count and hemoglobin levels.

The most important inclusion criteria for the above study is Spleen volume of 8 times (or more) the calculated volume for each patient (corresponding to body weight) according to the below formula:

$$\frac{\text{Measured volume.}}{\text{Calculated volume (0.2\% of body weight)}}$$

This is a multi-national multi-center study involving clinics around the world therefore all measurements should be performed according to the same protocol described herein below and all the data will be sent to a central clinical reader for volume evaluation. Central reading will be performed by: Dr. Blinder at MOR-MAR medical center using the imaging program: Kodak Algotec Medical (section 3.1).

Central Reading
Dr Geroge Blinder.MD
Head Radiologist & Medical Director
MAR Institute – Bikur Cholim Hospital, Jerusalem
And MOR MAR Imaging. ISRAEL

Imaging Program
Kodak Algotec Medical :
Certificated by FDA:
Medical Standarts:

FDA
ProVision - January 5, 1996 (K954678)
July 20, 1998 (K980648)
MediSurf - July 3, 1997 (K971347)
MediPrime - September 13, 2000 (K002894)
MediPrime - December 20, 2002 (K023936)

MediStore, CD-Surf, Auto-Router, Med-e-Mail and MediLink are all listed in the FDA Device Listing

2. Organ Volume measurements:
Spleen and liver volume will be determined at different time-points of the study: Screening, at 6 months and after 9 months of treatment (end of study).

Determination of organ volume will be done by MRI.

3. Equipment requirements:
   3.1 MRI scanner – at least 1.5 Tesla.
   3.2 Data storage – electronic media (CD), diacom format

4. Protocol:
MRI measurements will be performed at each site according to the MRI protocol described in table 1 below:

Table 1

No.	Name of sequence	Orient	Techn	TR	TE	Flip	Notes
1	Survey/sbc	MST (serview)	T1TFE	7.7	4.6	25	
2	SynBody REF	MST (serview)	FFE	8.0	0.83	7	
3	3D/TFE/RT	Coronary	T1TFE	9.3	4.6	25	2.5/5.0 slice thickness
4	T2TSE HR RT@	Coronary	TSE	1600	100	90	
5	T2TSE HR RT	Transverse	TSE	1600	100	90	

5. Initial evaluation:
Initial evaluation and diagnosis of each MRI will be performed by the site imaging expert under the responsibility of the site Principal Investigator for pathological findings.

6. Data Transfer:
MRI data will be transferred on CD in diacom format to MOR-MAR medical center in Jerusalem for organ volume determination.

Abbreviations:

3D/TFE	Three dimension turbo field echo
TSE	Turbo spin echo
FFE	Fast field echo
TR	Time of repetition
TE	Time to echo

23.3. Appendix 3. Vial Label

**Protocol # PB-06-001**<br>prGCD 200 units/vial<br>Protalix Biotherapeutics<br><br>For Intravenous infusion only as directed.<br><br>Batch number: XXXXXXDP<br>Exp.: MMM-YYYY<br><br>Limited by Law to Investigational Use<br>Subject number:____________<br>Subject initials:____________<br>Visit No. __________<br>Sponsor:<br>Protalix Biotherapeutics, 2 Snunit St.<br>Carmiel, Israel,	**Study Number: PB-06-001**<br>prGCD 200 units/vial<br>For Intravenous infusion only as directed<br>Batch number: XXXXXXDP<br>Exp.: MMM-YYYY<br><br>Limited by Law to Investigational Use<br>Subject number:____________<br>Subject initials:____________<br>Visit No. __________
