# Supplementary material for: Immunogenicity of glycans on biotherapeutic drugs produced in plant expression systems—The taliglucerase alfa story
Source: PLoS One. 2017 Oct 31;12(10):e0186211. doi: 10.1371/journal.pone.0186211 (PMC5663370; doi:10.1371/journal.pone.0186211)
Supplement: S2 File — (PDF) [file pone.0186211.s002.pdf]

# Clinical Protocol

## Protocol # PB-06-002

## A Phase 3 Multicenter, Open-label, Switchover Trial to Assess the Safety and Efficacy of Plant Cell Expressed Recombinant Human Glucocerebrosidase (prGCD) in Patients with Gaucher Disease Treated with Imiglucerase (Cerezyme®) Enzyme Replacement Therapy

Protalix
Biotherapeutics

Date of Original Protocol: 03 July 2008

**CONFIDENTIAL** **DO NOT COPY**

**This investigational protocol is the property of Protalix, Ltd and contains confidential information for use by the principal investigators and their designated representatives participating in this clinical investigation. It should be held confidential and maintained in a secure location. It should not be copied or made available for review by any unauthorized person or firm.**

**Protocol Title: A Phase 3 Multicenter, Open-label, Switchover Trial to Assess the Safety and Efficacy of Plant Cell Expressed Recombinant Human Glucocerebrosidase (prGCD) in Patients with Gaucher Disease Treated with Imiglucerase (Cerezyme®) Enzyme Replacement Therapy**

Protocol Number: PB-06-002

**Sponsor:**
Protalix Biotherapeutics
2 Snunit Street
Science Park
POB 455
Carmiel 20100, Israel
Ph: 972-4-988-9488
Fax: 972-4-988-9489

**CRO:**
Target Health Inc.
261 Madison Avenue, 24th Floor
New York, NY 10016
Ph: (212) 681-2100
Fax: (212) 681-2105

Cato Research Ltd.
2, Habarzel Street, 4th Floor
Ramat Hahayal
Tel Aviv, Israel 69710

Coordinating Investigator
Ari Zimran, M.D.
Associate Professor of Medicine
Director, Gaucher Clinic
Shaare Zedek Medical Center
Jerusalem, 91031, Israel
Ph: 972-2-6555673; Mobile: 972-545728284
Fax: 972-2-6517979

**Study Sites:**
Multicenter

| Confidentiality Statement |
| --- |
| The confidential information in this document is provided to you as a Principal Investigator or consultant for review by you, your staff, and the applicable Institutional Review Board/Independent Ethics Committee. Your acceptance of this document constitutes agreement that you will not disclose the information contained herein to others without written authorization from the Sponsor, Protalix Biotherapeutics. |

## 1. Protocol Synopsis

| | |
|---|---|
| Title of Study | A Phase 3 Multicenter, Open-label, Switchover Trial to Assess the Safety and Efficacy of Plant Cell Expressed Recombinant Human Glucocerebrosidase (prGCD) in Patients with Gaucher Disease Treated with Imiglucerase (Cerezyme®) Enzyme Replacement Therapy |
| | |
| Study Number | PB-06-002 |
| | |
| Investigational Product | Plant cell expressed recombinant human glucocerebrosidase (prGCD) |
| | |
| Indication | Treatment of Gaucher disease |
| | |
| Study Sites/Location | Multicenter |
| | |
| Objectives | The objective of this study is to assess the safety and efficacy of prGCD in patients with Gaucher disease currently being treated with Imiglucerase (Cerezyme®) enzyme replacement therapy (ERT). |
| | |
| Study Design | This is a multi-center, open-label, switchover trial to assess the safety and efficacy of prGCD in 15 patients with Gaucher disease who are currently being treated with imiglucerase (Cerezyme®) ERT. Eligible patients will enter a 12-week Stability Evaluation Period to establish the stability of their disease. Patients with stable disease will then be switched from their imiglucerase treatment to receive intravenous (IV) infusions of prGCD every two weeks for a total of 20 IV infusions. The dose of prGCD will be equal to each patient's previous imiglucerase dose. The infusions will be administered at the selected investigational site (clinic/hospital) At the end of the 9-month treatment period (20 visits, 38 weeks) eligible patients will be offered enrollment in an open-label extension study. |
| | |
| Number of Patients | 15 patients |
| | |
| Diagnosis and Main Criteria for Inclusion | Key Inclusion Criteria*:*<br>▪ Males and females, 18 years or older<br>▪ Female patients of child-bearing potential and male patients with female partners of child-bearing potential must agree to use a medically acceptable method of contraception, not including the rhythm method<br>▪ Diagnosis of Gaucher disease with leukocyte GCD activity level ≤3 nmol/mg*hr (≤30 % of the mean activity of the reference range)<br>▪ Stable Gaucher disease, defined as: |

| | |
|---|---|
| | a. Hemoglobin during Stability Evaluation Period is stable with no value more than 15% below or above the mean value<br>b. Platelets count during Stability Evaluation Period is stable with no values more than 40% below or above the mean value if the mean value is >120,000, or more than 20% below or above the mean value if the mean value is ≤ 120,000<br>c. No major surgery in the last year<br>d. No blood transfusion or major bleeding episode in the last year<br>e. No acute avascular necrosis event in the last year<br>f. No evidence of spleen or liver increasing enlargement while being treated with enzyme replacement therapy by palpation, ultrasound, or MRI over the last year<br>▪ Receiving imiglucerase therapy for at least 2 years and on a stable maintenance regimen (dose unchanged) for at least last six months<br>▪ Able to provide written informed consent<br><br>Key Exclusion Criteria<br>▪ Currently taking another experimental drug for any condition<br>▪ Pregnant or nursing or planning to become pregnant<br>▪ History of allergy to carrots<br>▪ Presence of anti-glucocerebrosidase (GCD) antibodies<br>▪ Previous infusion reaction suspected to be allergic in nature to Cerezyme® or Ceredase® or receiving premedication to prevent infusion reactions<br>▪ Presence of HIV and/or HBsAg and/or hepatitis C infection<br>▪ Presence of unresolved anemia due to iron, folic acid or vitamin B12 deficiency<br>▪ Presence of any significant comorbidity that could confound the interpretation of the clinical response to prGCD<br>▪ Presence of any medical, emotional, behavioral or psychological condition that in the judgment of the Investigator would interfere with the patient's compliance with the requirements of the study. |
| | |
| Discontinuation from Study | Patients will be discontinued from treatment with study drug if:<br><br>▪ Clinically relevant deterioration of the following parameters that is not stabilized by an optional increase in prGCD dose to a maximum of 60 U/kg<br>o Sustained reduction of platelet count from Baseline for three consecutive measurements two weeks apart: > 20% for baseline value of ≤ 120,000 and > 40% for baseline value of > 120,000, and<br>o Sustained reduction of hemoglobin from Baseline (>20%) for three consecutive measurements two |

| | |
|---|---|
| | weeks apart<br>▪ Two or more Grade 3 toxicities or one or more Grade 4 toxicity considered associated with prGCD treatment by the investigator<br>▪ Patient requests to discontinue treatment<br>▪ Investigator feels that it is not in the best interest of the patient to continue treatment and/or if the investigator believes that the patient can no longer be compliant with the requirements of the study<br>▪ Pregnancy in a female patient<br><br>For all patients who discontinue prematurely, the Investigator will obtain all required details and document the date and the primary reason of the premature termination. If the discontinuation is the result of an AE, the specific event or the main laboratory abnormality will be recorded in the CRF. The Investigator will make thorough efforts to document the outcome. |
| | |
| Treatment Groups | Open label treatment with prGCD |
| | |
| Drug Dosage and Route of Administration | prGCD dosage will be equal to each patient's previous imiglucerase dosing regimen. The route of administration will be IV infusion over 2 hours every 2 weeks |
| | |
| Duration of Treatment | 38 weeks |
| | |
| Study Endpoints | ▪ Adverse events<br>▪ Clinical laboratory (hematology, biochemistry, urinalysis)<br>▪ Anti human prGCD antibodies<br>▪ Electrocardiogram<br>▪ Echocardiogram<br>▪ Pulmonary function test<br>▪ Spleen volume<br>▪ Liver volume<br>▪ Platelet count<br>▪ Hemoglobin<br>▪ Biomarkers [chitotriosidase and pulmonary and activation-regulated chemokine (PARC/CCL18)] |
| | |
| Statistical Analysis | [illegible] it |

| | |
|---|---|
| | [illegible] |

**Table of Contents**

**1. Protocol Synopsis ........ 3**

**2. Introduction ........ 10**

**3. Objectives ........ 11**

3.1. Study Endpoints ........ 11

**4. Principal Investigator ........ 12**

**5. Institutional Review Board ........ 12**

**6. Informed Consent ........ 12**

**7. Patient Population, Number of Patients and Study Centers ........ 13**

**8. Treatment Group ........ 13**

**9. Randomization ........ 13**

**10. Patient Selection ........ 13**

10.1. Inclusion Criteria ........ 13
10.2. Exclusion Criteria ........ 14

**11. Study Design ........ 14**

**12. Study Visits ........ 14**

12.1. Stability Evaluation Period ........ 14
12.2. Treatment ........ 15

**13. Study Medication ........ 19**

13.1. Dosage ........ 19
13.2. Formulation ........ 20
13.3. Study Drug Administration ........ 20
13.4. Packaging ........ 20
13.5. Preparation and Labeling ........ 20
13.6. Storage ........ 21
13.7. Drug Accountability ........ 21

**14. Data Collection ........ 21**

**15. Statistical Section ........ 22**

15.1. Introduction ........ 22
15.2. Study Design ........ 22
15.3. Study Endpoints ........ 22
15.4. Study Populations ........ 23
15.5. Sample Size Justification ........ 23
15.6. Analyses ........ 23
15.7. Interim Safety Analysis ........ 24

**16. Safety Measurements ........ 24**

16.1. Patient Monitoring ........ 24
16.2. Adverse Events ........ 25

16.3. Serious Adverse Events ........ 25
16.4. Pregnancy ........ 26
16.5. Data Monitoring Committee ........ 26

**17. Concomitant Medication ........ 26**

17.1. Prohibited Concomitant Medication ........ 26
17.2. Allowed Concomitant Medication ........ 27

**18. Discontinuation from Study ........ 27**

**19. Study Records ........ 28**

**20. Reporting of Results ........ 28**

**21. Study Conduct ........ 29**

**22. REFERENCES ........ 30**

**23. Appendices ........ 32**

23.1. Appendix 1. Study Flow Chart ........ 33
23.2. Appendix 2. Infusion Rate Algorithm ........ 34
23.3. Appendix 3. MRI ........ 36
23.4. Appendix 4. Vial Label ........ 39
23.5. Appendix 5. Human prGCD Hypersensitivity Evaluation and Treatment Algorithm ...... 40
23.6. Appendix 6: WHO Common Toxicity Criteria ........ 43

Abbreviations:

| | |
|---|---|
| AE | Adverse Event |
| CRF | Case Report Form |
| CTM | Clinical Test Material |
| DEXA | Dual-energy X-ray Absorptiometry |
| DNA | Deoxyribonucleic Acid |
| ECHO | Echocardiogram |
| ERT | Enzyme Replacement Therapy |
| FDA | Food and Drug Administration |
| GCD | Glucocerebrosidase |
| GCP | Good Clinical Practice |
| ICF | Informed Consent Form |
| ICH | International Conference on Harmonization |
| IRB | Institutional Review Board |
| MedDRA | Medical Dictionary for Regulatory Authorities |
| MRI | Magnetic Resonance Imaging |
| PARC/CCL 18 | Chemokine (C-C motif) Ligand 18 (Pulmonary and Activation-regulated) |
| PI | Principal Investigator |
| prGCD | Plant Cell Expressed Recombinant Human Glucocerebrosidase |

## 2. Introduction

Gaucher disease, the most prevalent lysosomal storage disorder [1,2], is caused by mutations in the human glucocerebrosidase gene (GCD), which have been mapped to chromosome 1 q21-q31, leading to reduced activity of the lysosomal enzyme glucocerebrosidase and thereby to the accumulation of substrate glucocerebroside (GlcCer) in the cells of the monocyte-macrophage system. This accumulation leads to the visceral manifestations of hepatosplenomegaly, anemia and thrombocytopenia, as well as to the skeletal features and less frequently also to lung involvement [3].

The gene encoding human GCD was first sequenced in 1985 [4] The protein consists of 497 amino acids derived from a 536-mer pro-peptide. The mature hGCD contains five N-glycosylation amino acid consensus sequences (Asn-X-Ser/Thr). Four of these sites are normally glycosylated. Glycosylation of the first site is essential for the production of active protein. Both high-mannose and complex oligosaccharide chains have been identified [5]. hGCD from placenta contains 7% carbohydrate, 20% of which is of the high-mannose type [6]. Biochemical and site-directed mutagenesis studies have provided an initial map of regions and residues important to folding, activator interaction, and active site location [7].

The identification of GCD deficiency as the etiology of Gaucher disease stimulated the development of enzyme replacement therapy (ERT) as a therapeutic strategy for this disorder. ERT has been proven safe and effective over the past 14 years in over 4000 patients worldwide using either natural (placental-derived) or recombinant human glucocerebrosidase derived from mammalian tissue culture production systems. One of the major disadvantages with this treatment is its high cost, which approximately cost 250,000 dollars per patient per year [8-11]. Because the enzyme cannot cross the blood-brain-barrier, most of the patients who have been treated with ERT were those with Type I Gaucher disease, although patients with Type III also benefit from the effects of ERT on the visceral and skeletal manifestations. Similar results have been reported with both the placental derived enzyme (alglucerase; Ceredase™) which was approved by the FDA in 1991, and with the human recombinant enzyme (imiglucerase; Cerezyme™; both produced by Genzyme Therapeutics USA) which has been commercially available since 1994 [11].

Studies have shown that glycosylation plays a crucial role in glucocerebrosidase activity and uptake to target cells, and indeed, in the formulations currently used special steps of deglycosylation are required in order to generate exposed mannose residues as the terminal glycoside side chains [15-18].

Unmodified hGCD, derived from natural sources, cannot be targeted to the phagocytic cells in the body and is, therefore, of limited therapeutic value. In developing the current therapeutic products for Gaucher disease, the terminal sugars on the carbohydrate chains of hGCD are sequentially removed by treatment with three different glycosidases. This glycosidase treatment results in a glycoprotein whose terminal sugars consist of mannose residues. This facilitates uptake by mannose receptors on phagocyte cells that recognize glycoproteins and glycopeptides with oligosaccharide chains that terminate in mannose residues. The carbohydrate remodeling of hGCD improves the targeting of the enzyme to these cells [14-17].

Since the glycosylation pattern of hGCD must be remodeled to generate high mannose structures to increase uptake in target cells, the expression of hGCD in plant cells could be of great value. Post-translational modifications do not exist in bacterial expression systems, but plant derived expression systems do facilitate these modifications known to be crucial for protein expression and activity. One of the major differences between mammalian and plant protein expression systems is the variation of protein sugar side chains, caused by the differences in biosynthetic pathways. Glycosylation has been shown to have a profound effect on activity, folding, stability, solubility, and susceptibility to proteases, blood clearance rate and antigenic potential of proteins. Hence, any protein production in plants should take into consideration the potential ramifications of plant glycosylation. [12-13]

The production of prGCD utilizes *Agrobacterium tumefacience*, a bacterium capable of inserting single stranded DNA molecules into the plant genome. Due to the relative simplicity of introducing genes for mass production of proteins and peptides, this methodology is becoming increasingly popular as an alternative protein expression system. [1]

A Phase 1, single-center, non-randomized, open label, safety study with human prGCD administered to healthy volunteers was conducted. Three escalating single doses of human prGCD were administered as an intravenous (IV) infusion to six healthy volunteers. The vehicle was administered on Day 1, followed by human prGCD on Day 8 (15 units/kg), Day 15 (30 units/kg) and Day 22 (60 units/kg). Safety was the primary outcome and determination of pharmacokinetic parameters was the secondary outcome in this study.

Safety results indicate that prGCD was well tolerated with only minor side effects that were self limited and resolved with no treatment. prGCD administered intravenously once a week up to 60 mg/kg was shown to be safe and non immunogenic. For subjects receiving 30 and 60 units/kg, a mean half-life of approximately 13 minutes (range: 8-21 minutes) was determined. Exposure to prGCD was clearly dose-dependent and appeared to be nearly linear with dose. No gender differences in exposure were observed. Antibodies to test article were not found in any of the subjects.

Study PB-06-001, A Phase 3 Multicenter, Randomized, Double-Blind Trial to Assess the Safety and Efficacy of Two Parallel Dose Groups of Plant Cell Expressed Recombinant Human Glucocerebrosidase (prGCD) in Patients with Gaucher Disease, has been initiated under regulatory approvals obtained internationally. The objective of this study is to assess the safety and efficacy of prGCD in untreated patients with significant signs and symptoms of Gaucher disease. Patients receive IV infusion of prGCD every two weeks at the selected medical center. The duration of treatment is nine months.

## 3. Objectives

The objective of this study is to assess the safety and efficacy of prGCD in patients with Gaucher disease who are currently being treated with imiglucerase (Cerezyme®) ERT.

### 3.1. Study Endpoints

The safety of prGCD will be assessed by the following:

- Adverse events

- Clinical laboratory
    - Hematology: erythrocyte sedimentation rate, complete blood count; total white blood cell count, differential count (neutrophils, lymphocytes, monocytes, eosinophils and basophils), red blood cells, hematocrit, mean corpuscular volume, mean corpuscular hemoglobin and mean corpuscular hemoglobin concentration], and coagulation profile (prothrombin time, partial thromboplastin time)
    - Biochemistry: sodium, potassium, glucose, hemoglobin A1c, blood urea nitrogen, creatinine, calcium, phosphate (inorganic), uric acid, total protein, albumin, bilirubin (total), alkaline phosphatase, aspartate transaminase, alanine transaminase, gamma-glutamyl transferase, lactate dehydrogenase
    - Urinalysis: dipstick for presence of glucose, ketones and protein
- Anti human prGCD antibodies
- Electrocardiogram
- Echocardiogram
- Pulmonary function test

The efficacy of prGCD will be assessed by the following:

- Platelet count
- Hemoglobin
- Spleen volume
- Liver volume
- Biomarkers: chitotriosidase and PARC/CCL18

## 4. Principal Investigator

The Principal Investigator (PI) at each center has the responsibility for the conduct and compliance of this clinical trial according to this protocol and Good Clinical Practices (GCP) (ICH Guidance E6).

## 5. Institutional Review Board

This protocol and the Informed Consent Form must be reviewed and approved by the appropriate Ethics Committee/Institutional Review Board (IRB) associated with the study site. Any additional protocol amendments must be approved by the IRB prior to their implementation. A copy of the letter, signed by either the Chairman of the IRB or the Director General of the hospital (country dependent), to the Principal Investigator indicating IRB approval of the protocol must be received by the sponsor or designee and maintained in the study file prior to study initiation. Drug supply will not be shipped to the study site until the sponsor or designee receives this documentation.

## 6. Informed Consent

The risks and benefits of participating in this study will be explained to each potential patient prior to entering the study. The informed consent will be written in language readily understood by the patient. The informed consent must be approved by the IRB prior to study initiation, performance of any study procedure and dispensing of the study drug. The Principal

Investigator or his/her designee must obtain a signed and witnessed Informed Consent Form for each patient. Receipt of the signed Informed Consent Form will be documented in the Case Report Form and a copy retained by the Investigator. A copy of the signed Informed Consent Form will be given to each patient.

## 7. Patient Population, Number of Patients and Study Centers

Fifteen (15) patients with Gaucher disease, 18 years or older, who have been receiving imiglucerase (Cerezyme®) ERT for at least 2 years at a stable maintenance regimen (dose unchanged) for at least the last six months[19] will be enrolled at 5-8 investigational centers.

## 8. Treatment Group

Fifteen (15) eligible patients will be switched from their current imiglucerase ERT to open label treatment with prGCD. The dosage of prGCD will be equal to each patient's imiglucerase dose. IV infusion of prGCD will be administered bi-weekly for nine months.

## 9. Randomization

This is an open-label study. Randomization will not be performed.

## 10. Patient Selection

Potential patients identified by the principal investigator will sign informed consent at each site and will be screened to determine their eligibility according to the following inclusion and exclusion criteria. Once a patient has been found to be eligible, the Sponsor's Medical Director will review all patient screening data and provide final approval for enrollment.

### 10.1. Inclusion Criteria

Patients must meet all of the following criteria in order to be eligible to enter the study:

1. Males and females, 18 years or older
2. Female patients of child-bearing potential and male patients with female partners of child-bearing potential must agree to use a medically acceptable method of contraception, not including the rhythm method.
3. Diagnosis of Gaucher disease with leukocyte GCD activity level ≤3 nmol/mg*hr (≤30 % of the mean activity of the reference range)
4. Stable Gaucher disease, defined as:
    g. Hemoglobin during Stability Evaluation Period is stable with no value more than 15% below or above the mean value
    h. Platelets count during Stability Evaluation Period is stable with no values more than 40% below or above the mean value if the mean value is >120,000, or more than 20% below or above the mean value if the mean value is ≤ 120,000
    i. No major surgery in the last year
    j. No blood transfusion or major bleeding episode in the last year
    k. No acute avascular necrosis event in the last year
    l. No evidence of spleen or liver increasing enlargement while being treated with enzyme replacement therapy by palpation, ultrasound, or MRI over the last year

5. Receiving imiglucerase therapy for at least 2 years [19] and on a stable maintenance regimen (dose unchanged) for at least last six months
6. Able to provide written informed consent

## 10.2. Exclusion Criteria

Patients must be excluded if they meet any of the following criteria:

1. Currently taking another experimental drug for any condition
2. Pregnant or nursing or planning to become pregnant
3. History of allergy to carrots
4. Presence of anti-glucocerebrosidase (GCD) antibodies
5. Previous infusion reaction suspected to be allergic in nature to Cerezyme® or Ceredase® or receiving premedication to prevent infusion reactions
6. Presence of HIV and/or HBsAg and/or hepatitis C infections
7. Presence of unresolved anemia due to iron, folic acid or vitamin B12 deficiency
8. Presence of any significant comorbidity that could confound the interpretation of the clinical response to prGCD
9. Presence of any medical, emotional, behavioral or psychological condition that in the judgment of the Investigator would interfere with the patient's compliance with the requirements of the study.

## 11. Study Design

See Appendix 1 for the Study Flow Chart.

This is a multi-center, open-label, switchover trial to assess the safety and efficacy of prGCD in 15 patients with Gaucher disease who are currently being treated with imiglucerase ERT. Eligible patients will enter a 12-week baseline to establish the stability of their disease. Patients with stable disease will then be switched from their imiglucerase to receive IV infusions of prGCD every two weeks for a total of 20 infusions. The starting dose of prGCD will be equal to each patient's imiglucerase dose in the past 6 months. The infusions will be administered at the selected investigational site (clinic/hospital). The total duration of treatment will be nine months (38 weeks). At the end of the 9-month treatment period (20 visits, 38 weeks) eligible patients will be offered enrollment in an open-label extension study

## 12. Study Visits

## 12.1. Stability Evaluation Period

During the Stability Evaluation Period the patient will continue imiglucerase treatment. However, the screening visit should not be conducted within 5 days after an imiglucerase IV infusion to ensure an accurate baseline evaluation. Patients will be screened (Visit 0) and eligible patients will enter a 12-week Stability Evaluation Period (Visits A to E) to determine the stability of their disease. Hemoglobin and platelet count will be measured by local laboratory every two weeks for a total of 6 measurements. Patients with stable disease will then be eligible to continue to treatment with prGCD. Stable Gaucher disease during this period is defined as:

- Hemoglobin is stable with no value more than 15% below or above the mean value

- Platelet count is stable with no values more than 40% below or above the mean value if the mean value is >120,000, or more than 20% below or above the mean value if the mean value is ≤ 120,000

### 12.1.1. Screening (Visit 0, Week -12)

1. Obtain written informed consent from the patient
2. Assign screening number
3. Demographics
4. Medical history
5. Physical examination, including body weight
6. Current medications
7. Clinical laboratory tests
   - Blood platelet count including blood smear
   - Hemoglobin by local laboratory
   - Hematology
   - Biochemistry
   - Urinalysis
   - Anti-human prGCD antibodies
   - Thyroid stimulating hormone (TSH)
   - Serum iron, transferrin, ferritin
   - Vitamin B12
   - Folic Acid
   - Protein electrophoresis and immunoglobulin profile
   - Glucocerebrosidase activity
   - Blood pregnancy test (beta HCG)
   - Serology for HIV, HBsAg, HCV
8. Echocardiography
9. Chest X ray
10. Pulmonary function tests
11. Review all inclusion and exclusion criteria and determine patient's eligibility to continue for Disease Stability Evaluation

### 12.1.2. Disease Stability Evaluation (Visits A to E, Weeks -10 to -2 ± 3 days)

Blood will be drawn for analysis of platelet count and hemoglobin performed by the local laboratory at each visit to determine stability of these variables. After Visit E, the mean platelet count and hemoglobin values will be calculated and the values from each visit will be evaluated to determine if the variability is within the limits defined above. Patient eligibility will be reviewed by the Sponsor Medical Monitor. If approved for enrollment, the patient will be scheduled for Visit 1 to occur two weeks after the last imiglucerase infusion.

## 12.2. Treatment

### 12.2.1. Baseline (Visit 1, Day 1)

The following procedures will be performed:

1. Review of inclusion/exclusion criteria

2. Concomitant medications
3. Physical examination (including vital signs and body weight)
4. Clinical laboratory tests
   - Blood platelet count
   - Hemoglobin by local laboratory
   - Hematology
   - Biochemistry
   - Urinalysis
   - Anti human prGCD antibody test
   - Pregnancy test (Beta-HCG)
   - Biomarkers: chitotriosidase and PARC/CCL18
   - Mutation analysis
5. MRI for Spleen and Liver Volume (can be performed +/- 7d)
6. Electrocardiogram
7. Skeletal evaluation for general bone disease: full x-ray series if not available within 6 months prior to enrollment in the study; lumbar spine (lateral only), pelvis, femora and humeri
8. prGCD infusion

The following procedures will be performed after prGCD dosing:

- Patients will be observed clinically for a minimum of 1 hour after dosing.
- Vital signs will be evaluated every 15 minutes for the first hour and then every 30 minutes if the patient tolerates the infusion.
- The injection site will be evaluated.
- A follow up telephone call with the patient will be held the day after the infusion.

The patient should be reminded of the date of their next visit.

### 12.2.2. Visits 2 – 6 (Weeks 2, 4, 6, 8, 10 ± 3 Days)

Patients will receive their prGCD infusion at the selected medical center. The following procedures will be performed after dosing:

- The patients will be observed clinically for a minimum of 1 hour after dosing.
- Vital signs will be evaluated every 30 minutes if the patient has tolerated well previous infusions. Otherwise, vital signs should be evaluated every 15 minutes for the first hour.
- The injection site will be evaluated.

Adverse events and concomitant medications will be recorded at each visit.

Laboratory tests (hematology, biochemistry, urinalysis, anti human prGCD antibody test, and platelet count and hemoglobin by local laboratory) will be performed at visit 3 and 5 (Weeks 4 and 8).

The patient should be reminded of the date of their next visit.

### 12.2.3. Visit 7 (Week 12 ± 3 Days)

All patients will receive their prGCD infusion at the selected medical center. The following procedures will be performed:

1. Adverse events
2. Physical examination including vital signs and body weight
3. Concomitant medications
4. Blood samples for:
   - Blood platelet count
   - Hemoglobin by local laboratory
   - Hematology
   - Biochemistry
   - Anti-human prGCD antibody test, , including antibody subtype analysis and neutralizing antibodies in patients having a positive antibody response.
   - Blood pregnancy test (Beta-HCG)
   - Biomarkers: chitotriosidase and PARC/CCL18
5. Urine analysis
6. Electrocardiogram
7. prGCD infusion

The following procedures will be performed after dosing:

- The patients will be observed clinically for 1 hour after dosing.
- Vital signs will be evaluated every 30 minutes.
- The injection site will be evaluated.

The patient should be reminded of the date of their next visit.

### 12.2.4. Visits 8 – 13 (Weeks 14, 16, 18, 20, 22, and 24 ± 3 Days)

Patients will receive their prGCD infusion at the selected medical center. The following procedures will be performed after dosing:

- The patients will be observed clinically for 1 hour after dosing.
- Vital signs will be evaluated every 30 minutes.
- The injection site will be evaluated.

Safety data (adverse events and concomitant medications) will be recorded at each visit.

Samples for hematology, biochemistry, urinalysis, anti human prGCD antibodies, and platelet count and hemoglobin by local laboratory will be collected at Visit 10 (Week 18).

The patient should be reminded of the date of their next visit.

### 12.2.5. Visit 14 (Week 26 ± 3 days)

All patients will receive their prGCD infusion in the hospital/clinic and have the following evaluations.

1. Adverse events
2. Physical examination including vital signs and body weight
3. Concomitant medications
4. Blood samples for:
   - Blood platelet count
   - Hemoglobin by local laboratory
   - Hematology
   - Biochemistry
   - Anti-human prGCD antibody test, , including antibody subtype analysis and neutralizing antibodies in patients having a positive antibody response.
   - Biomarkers: chitotriosidase and PARC/CCL18 (PARC)
   - Blood pregnancy test, Beta-HCG
5. Urine analysis
6. Electrocardiogram
7. prGCD infusion

The following procedures will be performed after dosing:

- The patients will be observed clinically for 1 hour after dosing.
- Vital signs will be evaluated every 30 minutes.
- The injection site will be evaluated.

The patient should be reminded of the date of their next visit.

### 12.2.6. Visits 15 – 19 (Weeks 28, 30, 32, 34, and 36 ± 3 days)

Patients will receive their prGCD infusion at the selected medical center. The following procedures will be performed after dosing:

- The patients will be observed clinically for 1 hour after dosing.
- Vital signs will be evaluated every 30 minutes.
- The injection site will be evaluated.

Safety data (adverse events and concomitant medications) will be recorded at each visit.

Samples for hematology, biochemistry, urinalysis, anti human prGCD antibodies, and platelet count and hemoblogin will be collected at Visit 17 (Week 32).

The patient should be reminded of the date of their next visit.

### 12.2.7. Visit 20 (Week 38 ± 3 days)

All patients will receive their infusion in the hospital/clinic and have the following evaluations.

1. Physical examination including vital signs and body weight
2. Blood samples for:
   - Blood platelet count
   - Hemoglobin by local laboratory

- Hematology
- Biochemistry
- Biomarkers: chitotriosidase and PARC/CCL18 (PARC)
- Anti-human prGCD antibody test, including antibody subtype analysis and neutralizing antibodies in patients having a positive antibody response.
- Blood pregnancy test, Beta-HCG

3. Urine analysis
4. Echocardiography
5. Pulmonary function tests
6. MRI for Spleen and Liver Volume
7. Electrocardiogram
8. prGCD infusion
9. Adverse events
10. Concomitant medications

The following procedures will be performed after dosing:

- The patients will be observed clinically for 1 hour after dosing.
- Vital signs will be evaluated every 30 minutes.
- The injection site will be evaluated.

### 12.2.8. Premature Withdrawal Visit

All efforts will be made to complete the following procedures if a patient is prematurely withdrawn from the study before Week 38.

1. Physical examination including vital signs and body weight
2. Blood samples for:
    - Blood platelet count
    - Hemoglobin by local laboratory
    - Hematology
    - Biochemistry
    - Biomarkers: chitotriosidase and PARC/CCL18 (PARC)
    - Anti-human prGCD antibody test
    - Blood pregnancy test, Beta-HCG
3. Urine analysis
4. Echocardiography
5. Pulmonary function tests
6. MRI for Spleen and Liver Volume
7. Electrocardiogram
8. Adverse events
9. Concomitant medications

## 13. Study Medication

### 13.1. Dosage

The prGCD dosage will be equal to the patient's Imiglucerase dose. During treatment with prGCD, the dosage can be increased to a maximum dose of 60 U/kg if the patient experiences

deterioration in their Gaucher disease according to the criteria for platelet count and hemoglobin in Section 18. Discontinuation of Patients. A dosage increase requires Medical Director review and approval.

A unit of prGCD is defined as the amount of enzyme that catalyzes the hydrolysis of 1 micromole of the synthetic substrate para-nitrophenyl-beta-D-glucopyranoside (pNP-Glc) per minute at 37°C. Human prGCD vials are stored lyophilized at 2-8°C. The human prGCD final concentration is 40 U/mL after reconstitution with Sterile Water for Injection.

### 13.2. Formulation

Human prGCD is a purified recombinant, plant cell-expressed glucocerebrosidase, which is described in detail in the Investigator Brochure.

Each vial contains the following lyophilized contents:

200U of prGCD
195 mg mannitol
35 mg sodium citrate
0.53 mg polysorbate 80, NF

### 13.3. Study Drug Administration

Human prGCD will be administered via IV infusion at the rate of approximately 1.3 mL/min. At the rate of 1.3 mL/min the 135 mL volume plus a line flush of 20 mL of normal saline will be delivered over a 2 hour period. The rate of infusion can be increased to up to 2.25 mL/min if the infusion is tolerated well by the patient. The tolerability of the infusion will be determined by signs and symptoms during the infusion and observation for one hour after the infusion in the medical center, and also by telephone contact 24 hours after the infusion. **The infusion rate may be adjusted according to each individual patient's symptoms and signs (see Appendix 2. Infusion Rate Algorithm).**

### 13.4. Packaging

Lyophilized drug powder is stored in 13.5 mL borosilicate glass (Type 1) bottles (Forma Vitrum AG, Hungary). Lyophilization stoppers (Helvoet Pharma, Belgium) composed of two-leg brombutyl rubber are sealed with aluminum Snap Caps and polypropylen discs (Helvoet Pharma, Belgium).

### 13.5. Preparation and Labeling

The required amount of enzyme units for prGCD will be equal to each patient's previous Imiglucerase dose and will be adjusted with normal saline (0.9%) up to 135 ml/infusion.

Reconstitution of each vial (200 U) with sterile water for injection (5.1 mL) yields a final volume of 5.3 mL human prGCD (40 U/mL), which provides a withdrawal volume of 5.0 mL (200 enzyme units). The solution must be mixed gently until clear.

The label is in Appendix 3.

## 13.6. Storage

The product is stored at 2-8°C (36-46°F).

## 13.7. Drug Accountability

Protalix will provide drug accountability forms to assist the pharmacist in maintaining current and accurate inventory records covering receipt, dispensing, and the return of investigational drug supplies. When a shipment is received, the pharmacist will verify the quantities received and return the acknowledgment to the study monitor or designee. The pharmacist investigational drug accountability record includes the identification of the person to whom the drug is dispensed, the quantity and the date of dispensing and any returned or unused drug. This record is in addition to any drug accountability information recorded on the Case Report Form (CRF). These records will be readily available for inspection by a monitor or Protalix audits and are open to regulatory authority inspection at any time.

# 14. Data Collection

The electronic Case Report Form (eCRF) is an integral part of the study and subsequent reports. The eCRF must be used to capture all study data recorded in the patient's medical record (source document). The patient screening and identification number and initials will be used to identify the patient.

All protocol-required information collected during the study must be entered by the Investigator, or designated representative, in the Target e*CRF™, an Internet-based data collection system. All details for the completion and correction of the eCRF will be explained to the investigator. The management module of Target e*CRF™, includes edit check and query systems that seamlessly integrate with the data entry system. All modifications to the data in the eCRF are tracked by an electronic audit trail (date and identity of the person making the change are instantaneously recorded). Target e*CRF™ is 21CFR Part 11 compliant.

The names, positions, and signatures of all persons authorized by the Investigator to make entries in the eCRF must be supplied to the sponsor.

The Investigator, or designated representative, should complete the eCRF as soon as possible after information is collected, preferably on the same day that a study patient is seen for an examination, treatment, or any other study procedure. Any outstanding entries must be completed immediately after the final examination. By design, an explanation must be provided for all missing data and/or out of range data.

The completed case report form must be reviewed and signed by the Investigator named in the study protocol or by a designated sub investigator.

The monitor is responsible for performing on-site monitoring at regular intervals throughout the study to verify adherence to the protocol; verify adherence to local regulatory requirements on the conduct of clinical research; and ensure completeness, accuracy, and consistency of the data entered in the eCRF by comparison to source documents at the site.

Final monitored and audited eCRFs will be provided by the Sponsor to the sites at the end of the study in the format of a PDF file.

## 15. Statistical Section

6

## 16. Safety Measurements

### 16.1. Patient Monitoring

Patients will be monitored at all visits, to determine the occurrence of adverse events. Patients will be observed for the occurrence of adverse events for one hour following the IV infusion including hypersensitivity reactions (see Appendix 5, Section 23.5). Patients who experience an adverse event will be followed until the event resolves, is stable for 30 days, or until 30 days after the last infusion of prGCD. Patients who experience a Grade 3 or Grade 4 adverse event will be followed for 60 days after the event resolves, except for events that are considered Definitely Not Related to prGCD. Patients who experience progressive hypersensitivity or severe hypersensitivity will be treated appropriately and withdrawn from the study (see Section 23.5. Appendix 5).

A sustained reduction in platelet count and hemoglobin that is not stabilized by an optional increase in prGCD dose to a maximum of 60 U/kg will result in withdrawal of patients from the study (see Section 18. Discontinuation of patients). If a reduction in platelets and hemoglobin meeting the criteria described in Section 18 occurs at a routine visit, three consecutive weekly measurements will be analyzed at the local laboratory to determine whether the reduction is sustained.

## 16.2. Adverse Events

An adverse event is any undesirable, unintentional or unexpected (unanticipated) event that occurs throughout the study, whether or not considered related to the drug. Adverse events will be monitored throughout the study, and recorded in the eCRF. Adverse events resulting from concurrent illnesses or reactions to concurrent medications are also to be recorded. In order to avoid vague expressions, the adverse event will be recorded in standard medical terminology. WHO Common Toxicity grade will be used (See Section 23.6, Appendix 6). For adverse events not specifically included in the WHO Common Toxicity Criteria table, the following categories will be used to record the severity of the event.

| DEGREE OF INTENSITY | DESCRIPTION |
|---|---|
| Mild (Grade 1) | Awareness of signs and symptoms; easily tolerated |
| Moderate (Grade 2) | Discomfort sufficient to interfere, but not prevent, daily activity |
| Severe (Grade 3) | Unable to carry out usual activity |
| Very Severe (Grade 4) | Incapacitating, requires hospitalization, results in death |

Action taken - whether or not the adverse event required treatment, caused the patient to be withdrawn from the study, or required other action

Relationship - whether or not the test product caused the adverse event

| DEGREE | DESCRIPTION |
|---|---|
| Definitely | There is evidence of exposure to the test product, for example, reliable history or acceptable compliance assessment; the temporal sequence of the AE onset relative to the drug is reasonable; the AE is most likely to be explained by the drug treatment than by another cause; the challenge is positive; re-challenge (if feasible) is positive; the AE shows a pattern consistent with previous knowledge of the drug treatment. |
| Probably | There is evidence of exposure to the test product; the temporal sequence of the AE onset relative to the drug administration is reasonable; the AE is more likely explained by the drug treatment than by another cause; the challenge (if performed) is positive. |
| Possibly | There is evidence of exposure to the test product; the temporal sequence of the AE relative to the drug administration is reasonable; the AE could have been due to another equally likely cause; the challenge (if performed) is positive. |
| Probably not | There is evidence of exposure to the drug; there is another more likely cause of the AE; the challenge (if performed) is negative or ambiguous; rechallenge (if performed) is negative or ambiguous. |
| Definitely not | The patient/patient did not receive the drug treatment; or temporal sequence of the AE onset relative to administration of the drug is not reasonable; or there is another obvious cause of the AE. |

## 16.3. Serious Adverse Events

A serious adverse event is any event that suggests a significant hazard, contraindication, side effect, or precaution. With respect to human clinical events, a serious adverse event includes any event that is fatal or life threatening requires hospitalization or extends the hospitalization of hospitalized patients, results in a persistent or significant disability/incapacity, or a congenital anomaly/birth defect.

The Investigator shall, within 24 hours of occurrence or notification of an SAE, report the SAE to the Medical Monitor and/or the Clinical Research Associate, who will then inform the Sponsor of the serious adverse event. SAEs may need to be reported to all IECs/IRBs according to local requirements and applicable health authorities in accordance with applicable regulatory requirements.

The sponsor shall notify the appropriate regulatory authorities as required and all participating Investigators of any adverse event associated with use of the drug that is both serious and unexpected. The Investigator must also notify the IRB.

### 16.4. Pregnancy

Although pregnancy as such is not considered an AE or SAE, it is the responsibility of the Investigator to report to Protalix, by telephone immediately, any pregnancy occurring in a female study patient or female partner of a male study patient either during the study or within 42 days following the last dose of study drug. Protalix will provide the Investigator with a Pregnancy Tracking Form that is to be completed by the study site on a periodic basis and faxed to Protalix. The Investigator will follow the pregnancy until the end of the pregnancy. If the pregnancy continues to term (delivery), the health of the infant must also be reported to Protalix.

### 16.5. Data Monitoring Committee

A Data Monitoring Committee (DMC) will be constituted to monitor safety of human prGCD in this study. The charter for the DMC will be approved before initiation of the study as a separate document. The responsibilities of the DMC will be to conduct *ad hoc* safety analyses and clinical review of patients who meet the protocol criteria for deterioration ((Section 18. Discontinuation of Patients).

The study will be stopped and an *ad hoc* safety analysis will be conducted by the DMC if one of the two following criteria is met:

- WHO Grade 3 toxicity associated with prGCD treatment is experienced by two or more patients
- WHO Grade 4 toxicity associated with prGCD treatment is experienced by one or more patients

Based on the recommendation of the DMC, the study may be restarted or terminated following consultation with the Food and Drug Administration and other regulatory authorities.

## 17. Concomitant Medication

All medications taken by or administered to the patient will be recorded in the CRF.

### 17.1. Prohibited Concomitant Medication

The following medications are strictly prohibited during the study:

- Zavesca® (miglustat)
- Ceredase® (alglucerase)

- Cerezyme® (imiglucerase) (allowed after screening visit until two weeks before Day 1)

## 17.2. Allowed Concomitant Medication

The following medications are allowed and expected during the study:

- Treatments for hypersensitivity, anaphylaxis, or anaphylactoid reactions; e.g., epinephrine, norepinephrine, glucagons, albuterol
- Treatments for anemia; e.g., iron, folic acid, vitamin B12
- Treatments for bone disease; e.g., biphosphonates
- Analgesics; e.g., nonsteroidal anti-inflammatory drugs (note: aspirin may be contraindicated due to its effects on platelets and risk for bleeding)

# 18. Discontinuation from Study

Patients will be discontinued from treatment with study drug if the following stopping rules are met:

- Clinically relevant deterioration of the following medical parameters as defined as:

    - Sustained reduction of platelet count from the mean of 6 Stability Evaluation Period measurements for three consecutive measurements 2 weeks apart: > 20% for baseline of ≤ 120,000 and > 40% for baseline of > 120,000, and/or
    - Sustained reduction (>20%) of hemoglobin from baseline (mean of 6 baseline measurements) for three consecutive measurements 2 weeks apart

    Patients who meet these criteria and are being treated with the highest permissible dose of prGCD of 60 U/kg will be reviewed by the DMC. The DMC will provide their recommendation to the Investigator and Medical Director before final discontinuation.

    Patients who meet these criteria and are not being treated with the highest permissible dose of prGCD of 60 U/kg are eligible to have their dose increased at the discretion of the Investigator and approval of the Medical Director. Subsequently, the mean of the three consecutive measurements taken 2 weeks apart described above will serve as the baseline for detecting further clinically relevant deterioration after the dose increase is implemented. The following stopping rule will be applied.

    - Sustained reduction of platelet count from the mean of 3 measurements (new baseline) for three additional consecutive measurements 2 weeks apart: > 20% for baseline of ≤ 120,000 and > 40% for baseline of > 120,000, and/or
    - Sustained reduction (>20%) of hemoglobin from baseline (mean of 3 measurements – new baseline) for three additional consecutive measurements 2 weeks apart

    Patients who meet these criteria will be reviewed by the DMC. The DMC will provide their recommendation to the Investigator and Medical Director before final discontinuation.

- Two or more Grade 3 toxicities or one or more Grade 4 toxicity considered associated

with prGCD treatment by the investigator

- Patient requests to discontinue treatment
- Investigator feels that it is not in the best interest of the patient to continue treatment and/or if the investigator believes that the patient can no longer be compliant with the requirements of the study
- Pregnancy in a female patient

For all patients who discontinue prematurely, the Investigator will obtain all required details and document the date and the primary reason of the premature termination. If the discontinuation is the result of an AE, the specific event or the main laboratory abnormality will be recorded in the CRF. The Investigator will make thorough efforts to document the outcome.

## 19. Study Records

Study records including case report forms (CRFs), patient progress notes, original copies of test results, signed informed consent forms, patient enrollment log, drug dispensation log, Institutional Review Committee approval letters, and other documents pertaining to the conduct of the study must be kept on file by the Investigator. Study records are to be available for sponsor inspection at any time.

All study records will be retained for a period of time as defined by the regulatory authority for the country in which the investigation is conducted. Generally, this means at least 2 years following the date on which the drug is approved by the regulatory authority for marketing for the purposes that were the subject of the investigation. In other situations (e.g., where the investigation is not in support of or as part of an application for a research or marketing permit), for a period of 2 years following the date on which the entire clinical program is completed, terminated or discontinued or the investigational application under which the investigation is being conducted is terminated or withdrawn by the regulatory authorities.

In the event the Investigator retires, relocates or for any other reason withdraws from the responsibility for maintaining records for the period of time required, custody of the records may be transferred to any other person who will accept responsibility for the records. Notice of such a transfer must be given in writing to the Sponsor. The Investigator must contact the Sponsor prior to disposal of any records related to this study.

## 20. Reporting of Results

The Investigator will record all drug administration data, results of laboratory tests, side effects and efficacy data on the Case Report Form (CRF). Photocopies of original laboratory slips or computer printout of the relevant data must be available for inspection by the sponsor upon request.

## 21. Study Conduct

THIS STUDY WILL BE CONDUCTED IN ACCORDANCE WITH GOOD CLINICAL PRACTICE REQUIREMENTS.

APPROVED: ___________________________________
(Investigator's Signature)

DATE APPROVED: ______________________________

APPROVED: ___________________________________
(Sponsor's Signature)

Title: __VP Product Development_____________

DATE APPROVED: ______________________________

## 22. REFERENCES

1. Lee, RE. The pathology of Gaucher disease. Prog Clin Biol Res 1982;95:177-217.
2. Grabowski G. Gaucher disease: enzymology, genetics, and treatment. Adv Hum Genet. 1993;21:377-441.
3. Grabowski GA, Hopkin RJ. Enzyme Therapy for Lysosomal Storage Disease: Principles, Practice, and Prospects. Annual Review of Genomics and Human Genetics 2003;4:403-436.
4. Sorge JWC, Westwood B, Beutler E. Molecular Cloning and Nucleotide Sequence of Human Glucocerebrosidase cDNA. Proc Natl Acad Sci USA. 1985;82:7289-7293.
5. Berg-Fussman A, Grace M, Ioannou Y, and Grabowski G. Human acid beta-glucosidase. N-glycosylation site occupancy and the effect of glycosylation on enzymatic activity. J. Biol. Chem. 1993;268:14861-14866.
6. Grace M, Grabowski GA. Human acid ß-glucosidase: glycosylation is required for catalytic activity. Biochem Biophys Res Commun 1990;168:771-777.
7. Grace M, Newman K, Scheinker V, Berg-Fussman A, and Grabowski G. Analysis of human acid beta-glucosidase by site-directed mutagenesis and heterologous expression. J. Biol. Chem. 1994;269:2283-2291.
8. Barton NW, Brady RO, Dambrosia JM, Di Bisceglie AM, Doppelt SH, Hill SC, Mankin HJ, Murray GJ, Parker RI, Argoff CE, et al. Replacement therapy for inherited enzyme deficiency macrophage-targeted glucocerebrosidase for Gaucher's disease. N Engl J Med. 1991;324:1464-1470.
9. Grabowski GA, Barton NW, Pastores G, Dambrosia JM, Banerjee TK, McKee MA, Parker C, Schiffmann R, Hill SC, and Brady RO. Enzyme Therapy in Type I Gaucher disease: Comparative Efficacy of Mannose-terminated Glucocerebroidase from Natural and Recombinant Sources. Ann Intern Med 1995;122:33-39.
10. Pastores GM, Sibille AR, Grabowski GA. Enzyme therapy in Gaucher disease type 1: dosage efficacy and adverse effects in 33 patients treated for 6 to 24 months. Blood. 1993;82:408-416.
11. Weinreb NJ, Charrow J, Andersson HC, Kaplan P, Kolodny EH, Mistry P, Pastores G, Rosenbloom BE, Scot, CR, Wappner RS, Zimran A. Effectiveness of enzyme replacement therapy in 1028 patients with type 1 Gaucher disease after 2 to 5 years of treatment: a report from the Gaucher Registry. Am J Med. 2002;113:112-119.
12. Ma JKC, Drake PMW, and Christou P. The production of recombinant pharmaceutical proteins in plants. Nature reviews Genetics. 2003;4:794-805.
13. Lerouge P, Cabanes-Macheteau M, Rayon C, Fischette-Laine AC, Gomord V, Faye L. N-glycoprotein biosynthesis in plants: recent developments and future trends. Plant Mol Biol. 1998;38:31-48.
14. Anand G. Uncertain miracle: A biotech drug extends a life, but at what price? The Wall Street Journal November 16, 2005; Page A1.
15. Bijsterbosch MK, Donker W, van de Bilt H, van Weely S, van Berkel TJ, Aerts JM. Quantitative analysis of the targeting of mannose-terminal glucocerebrosidase. Predominant uptake by liver endothelial cells. Eur J Biochem 1996;237:344-349.
16. Friedman B, Vaddi K, Preston C, Mahon E, Cataldo JR and McPherson JM. A Comparison of the Pharmacological Properties of Carbohydrate Remodeled Recombinant and Placental-Derived beta -Glucocerebrosidase: Implications for Clinical Efficacy in Treatment of Gaucher Disease. Blood 1999;93:2807-2816.
17. Furbish FS, Steer CJ, Krett NL, Barranger JA. Uptake and distribution of placental

glucocerebrosidase in rat hepatic cells and effects of sequential deglycosylation. Biochim Biophys Acta. 1981;673:425-434.

18. Doebber T, Wu M, Bugianesi R, Ponpipom M, Furbish F, Barranger J, Brady R, and Shen T. Enhanced macrophage uptake of synthetically glycosylated human placental beta-glucocerebrosidase. J. Biol. Chem. 1982;257:2193-2199.
19. Pastores G M, Weinreb N J, Aerts H et al., “Therapeutic goals in the treatment of Gaucher disease”, Semin. Hematol. (2004); 41(4 Suppl 5): pp. 4–14.
20. Schwartz LB. Diagnostic Value of Tryptase in Anaphylaxis and Mastocytosis. Immunol Allergy Clin N Am 2006;26:451-463.
21. Dybendal T, Guttormsen AB, Elsayed S, Askeland B, Harboe T and Florvaag E. Screening for mast cell tryptase and serum IgE antibodies in 18 patients with anaphylactic shock during general anaesthesia. Acta Anaesthesiol Scand 2003;47:1211-1218.
22. Lieberman P, Kemp SF, Oppenheimer J *et al*. The Diagnosis and Management of Anaphylaxis: An Updated Practice Parameter. J Allergy Clin Immunol 2005;115:S483-523.
23. Ebo DG, Fisher MM, Hagendorens MM, Bridts CH, Stevens WJ. Anaphylaxis during anaesthesia: diagnostic approach. Allergy 2007;62:471-487.
24. Schwartz LB, Yunginger JW, Miller J, Bokhari R and Dull D. Time Course of Appearance and Disappearance of Human Mast Cell Tryptase in the Circulation after Anaphylaxis. J Clin Invest 1989;83:1551-1555.

## 23. Appendices

## 23.1. Appendix 1. Study Flow Chart

| Activity | Visit 0 Screening | Visits A-E[3] | Visit 1 Baseline | Visits 2-6 | Visit 7 Month 3 | Visits 8-13 | Visit 14 Month 6 | Visits 15-19 | Visit 20 Month 9 | Premature Withdrawal[1] |
|---|---|---|---|---|---|---|---|---|---|---|
| | Week -12[1] | Weeks -10, -8, -6, -4, -2 | Day 1[1] | Weeks 2, 4[1], 6, 8[1], 10 | Week 12[1] | Weeks 14, 16, 18[1], 20, 22, 24 | Week 26[1] | Weeks 28, 30, 32[1], 34, 36 | Week 38[1] | |
| Sign informed consent | X | | | | | | | | | |
| Medical History | X | | | | | | | | | |
| Demographics | X | | | | | | | | | |
| Review Inclusion/Exclusion Criteria | X | | X | | | | | | | |
| Physical Examination | X | | X | | X | | X | | X | X |
| Blood platelet count | X[2] | X | X | X[1] | X | X[1] | X | X[1] | X | X[1] |
| Hemoglobin by local lab | X | X | X | X[1] | X | X[1] | X | X[1] | X | X[1] |
| Weight | X | | X | | X | | X | | X | X |
| Current/Concomitant Medications | X | | X | X | X | X | X | X | X | X |
| Hematology | X | | X | X[1] | X | X[1] | X | X[1] | X | X[1] |
| Biochemistry | X | | X | X[1] | X | X[1] | X | X[1] | X | X[1] |
| Urinalysis | X | | X | X[1] | X | X[1] | X | X[1] | X | X[1] |
| Anti-Human prGCD Antibodies | X | | X | X[1] | X | X[1] | X | X[1] | X | X[1] |
| TSH, iron, ferritin, transferrin, B12 and folic acid | X | | | | | | | | | |
| Protein electrophoresis | X | | | | | | | | | |
| Beta HCG | X | | X | | X | | X | | X | X |
| Glucocerebrosidase | X | | | | | | | | | |
| Serology | X | | | | | | | | | |
| Echocardiography | X | | | | | | | | X | X |
| Chest X-Ray | X | | | | | | | | X | X |
| Pulmonary Function Tests | X | | | | | | | | X | X |
| Vital signs | | | X | | X | | X | | X | X |
| Biomarkers | | | X | | X | | X | | X | X |
| Mutation analysis | | | X | | | | | | | |
| Organ Volumes (MRI) | | | X | | | | | | X | X |
| Electrocardiograph | | | X | | X | | X | | X | X |
| X-RAY Skeketal Evaluation | | | X | | | | | | | |
| prGCD Infusion | | | X | X | X | X | X | X | X | |
| Adverse Events | | | X | X | X | X | X | X | X | X |

[1]Hematology and biochemistry (central lab), urinalysis, platelet count, and hemoglobin (local lab), and anti-human prGCD antibodies will be performed at visits 0, 1, 4, 8, 12, 18, 26, 32 and 38, and also in case of premature withdrawal. [2]Include blood smear for platelet aggregates. [3]Visits A to E include only platelet count and hemoglobin by local lab

## 23.2. Appendix 2. Infusion Rate Algorithm

The infusion rate (IR) may be adjusted according to individual patient symptoms and signs. The assumptions with respect to adverse experiences to the infusion are:

1. Most of the patients will tolerate well the infusion without any special symptom or event and the infusion rate may be increased over time to the maximal rate 2.25 ml/minor (an infusion duration of 1 hour).
2. Patients presenting symptoms and signs of **severe** hypersensitivity will be evaluated according to the WHO Drug Toxicity criteria and there may be a discontinuation of treatment according to the protocol.
3. Patients may present signs and symptoms that will respond to reducing of the infusion rate and may not appear at the next infusion.
4. Tolerability and the patient specific infusion rate will be assessed and decided by the Investigator according to vital signs and clinical status of the patient.

Definitions to be applied regarding tolerability of infusions are as follows:

| Good tolerability | Partial tolerability | Poor tolerability |
|---|---|---|
| Infusion was performed without Signs and symptoms (such as burning, pruritus, flushing, discomfort, or change in vital signs). | Signs and symptoms appeared during the infusion **and resolved after slowing infusion rate** or at the end of the infusion. | Signs and symptoms meeting the definitions of WHO Grade 1 or 2 toxicity responding to reduction of infusion rate or responding to treatment (example, antihistamine for urticaria). |

The specific algorithm to be followed is below.

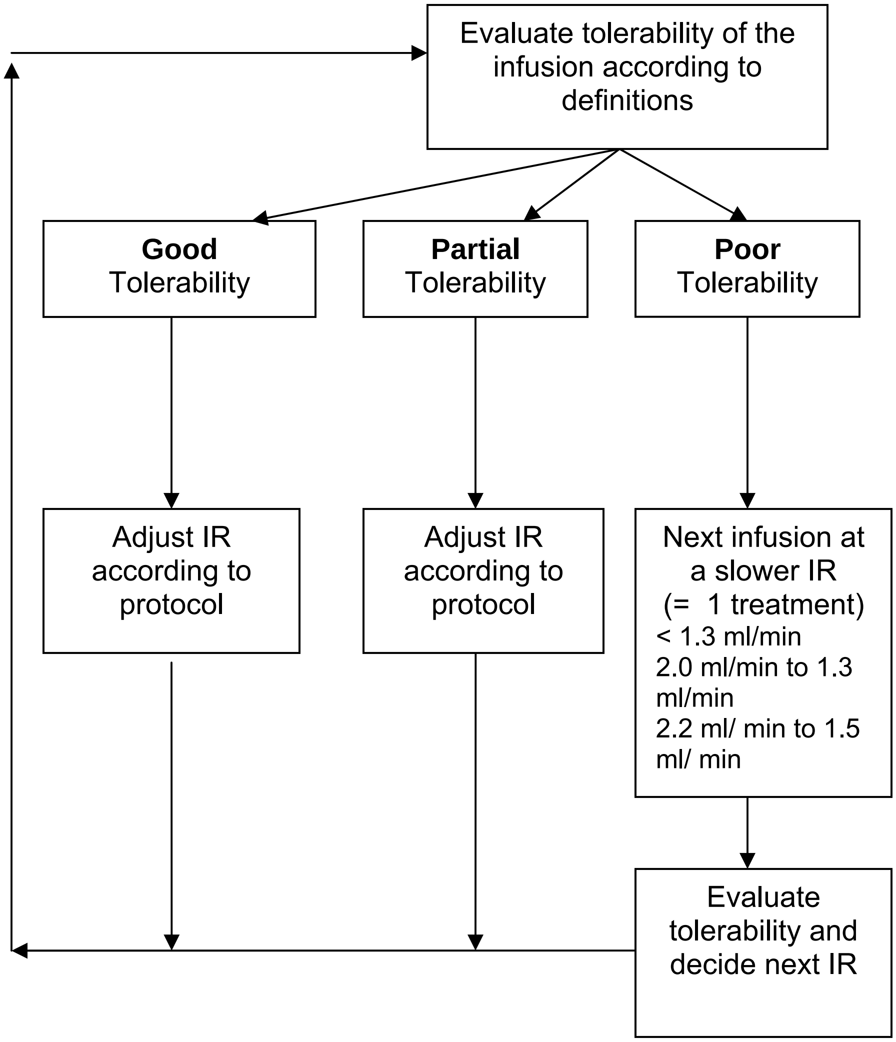
Evaluate tolerability of the infusion according to definitions
**Good** Tolerability
**Partial** Tolerability
**Poor** Tolerability
Adjust IR according to protocol
Adjust IR according to protocol
Next infusion at a slower IR (= 1 treatment)
< 1.3 ml/min
2.0 ml/min to 1.3 ml/min
2.2 ml/ min to 1.5 ml/ min
Evaluate tolerability and decide next IR

## 23.3. Appendix 3. MRI

### 23.3.1. Magnetic Resonance Imaging (MRI) data

Each patient enrolled in this trial will have 2 MRI timepoints during the course of the trial:

- Baseline (visit 1)
- Month 9 (visit 20)

For each timepoint, the same set of T1 and T2-weighted MRI sequences will be acquired. These sequences will be defined based on their equipment and abilities to provide sufficient image quality and contrast for organ (spleen and liver) detection and quantification, and also full spleen and liver coverage in Gaucher patients. No contrast agent will be used.

### 23.3.2. MRI evaluation parameters

The following MRI parameters will be evaluated during this trial:

- Volume of spleen (in $cm^3$)
- Volume of liver (in $cm^3$)

### 23.3.3. Sites and image data management

All image management activities will be centralized and conducted by an independent imaging Contract Research Organization (imaging CRO) with operational capabilities in Europe and the United States in compliance with all regulatory requirements. An overview of the main activities performed by the imaging CRO is provided in the next sections.

#### 23.3.3.1. Standardization of image acquisition, initial site qualification

The image acquisition procedure will be standardized by the imaging CRO among all participating sites. The same image acquisition and management procedure will be used by all sites. This procedure will be defined by the imaging CRO and approved by the Sponsor. The sites will be trained and qualified by the imaging CRO prior to start of patient enrolment. Each site will provide test MRI scan(s) during the initial site qualification phase. The source of the test scan(s) will be (in order of preference) a patient volunteer, a healthy volunteer, or the screening image from the first patient tested at the site. All images will be provided in digital format (DICOM). Only digital images will be centrally processed by the imaging CRO.

#### 23.3.3.2. Quality control of image data and site Quality Assurance during the course of the trial

The image data will be collected and quality controlled by the imaging CRO for checking the technical adequacy, the compliance of data acquisition with the study imaging protocol, and the diagnostic quality of the images (their appropriateness for centralized evaluations). If any quality-related issue is detected by the imaging CRO, specific queries will be sent to the sites to implement appropriate corrective (such as potential repeat scans whenever possible) and preventive actions.

### 23.3.4. Image processing and centralized analysis

#### 23.3.4.1. Spleen and liver segmentation

In order to improve the quality of centralized image evaluations, the anatomical structures of interest (liver and spleen) will be pre-detected using automated three-dimensional (3D) segmentation software. 3D segmentation consists in classifying image voxels in multiple tissue classes based on the MRI signal intensities and also the piecewise contiguity of image voxels representing given organs (liver and spleen in this case). The resulting contours representing the liver and the spleen will be manually edited and quality controlled by an expert technician at the imaging CRO. The resulting liver and spleen contours will be submitted for final approval to independent radiologists.

#### 23.3.4.2. Centralized and Image Review

The MRI data will be centrally evaluated by independent radiologists. The reading sessions will be organized at the imaging CRO site. The same image evaluation procedure will be used by all radiologists and for all patients' MRI scans in this trial.

The radiologists will be Senior Radiologists with a significant experience in liver and spleen imaging and MRI. The radiologists will provide detailed, signed and dated CVs including their scientific references. The imaging CRO will subcontract with the radiologists for the payment of their honoraria.

The radiologists will be trained prior to start of centralized image review sessions. They will be provided with a Reader's Kit including an Image Evaluation Software User Guide.

All evaluation results including spleen and liver contours, and the imaging CRO electronic Case Report Forms (eCRFs) will be saved in the trial database. In compliance with regulatory requirements (including FDA's 21 CFR Part 11), audit trails will be generated for all image manipulation and evaluation steps and the radiologists will use an electronic signature system to authenticate themselves for the evaluation of each MRI timepoint. The trial specific Image Review Software will be developed, validated and documented by the imaging CRO in compliance with regulatory requirements.

### 23.3.5. Data and report transfers to Sponsor

- Image Review sessions will be exported to the Sponsor using a pre-defined, standardized and secure data transfer procedure.
- Patient and MRI data tracking information (study progress reports) will be communicated to the Sponsor using a pre-defined, standardized and secure data transfer procedure.
- The final Study database will be submitted to the Sponsor in digital format.
- Spleen and liver volumes will be directly computed from organ masks validated by the radiologists. The result of this quantification will be stored in the database. The file containing final results will be automatically generated from the values of the database, thus avoiding any result to be modified after the review sessions.

### 23.3.6. Direct access to Study data

- Direct access to Study data will be made possible by the imaging CRO for audit purposes.
- Such Study data include:
  - Information related to interactions between the imaging CRO and the sites (Queries, Data Clarification Forms, test data submitted by the sites, etc.)
  - Native MRI data
  - Data processed and generated by the imaging CRO
  - Data generated by the radiologists (including image masks of liver and spleen and eCRFs)
  - Audit trails

### 23.3.7. Unevaluable MRI

Unevaluable MRI data can result from a poor quality image, due to patient motion, improper organ coverage, technical problems with the image transmission to the imaging CRO, etc. The imaging CRO procedures for ensuring quality images are meant to reduce or eliminate such poor quality images (Section IV.2 above).

If an adequate patient image cannot be obtained for a given time point in the study, the problem with the image will be documented at the imaging CRO and in the e*CRF™. In addition, the imaging CRO will document all attempted corrective actions with the investigative site imaging centre.

## 23.4. Appendix 4. Vial Label

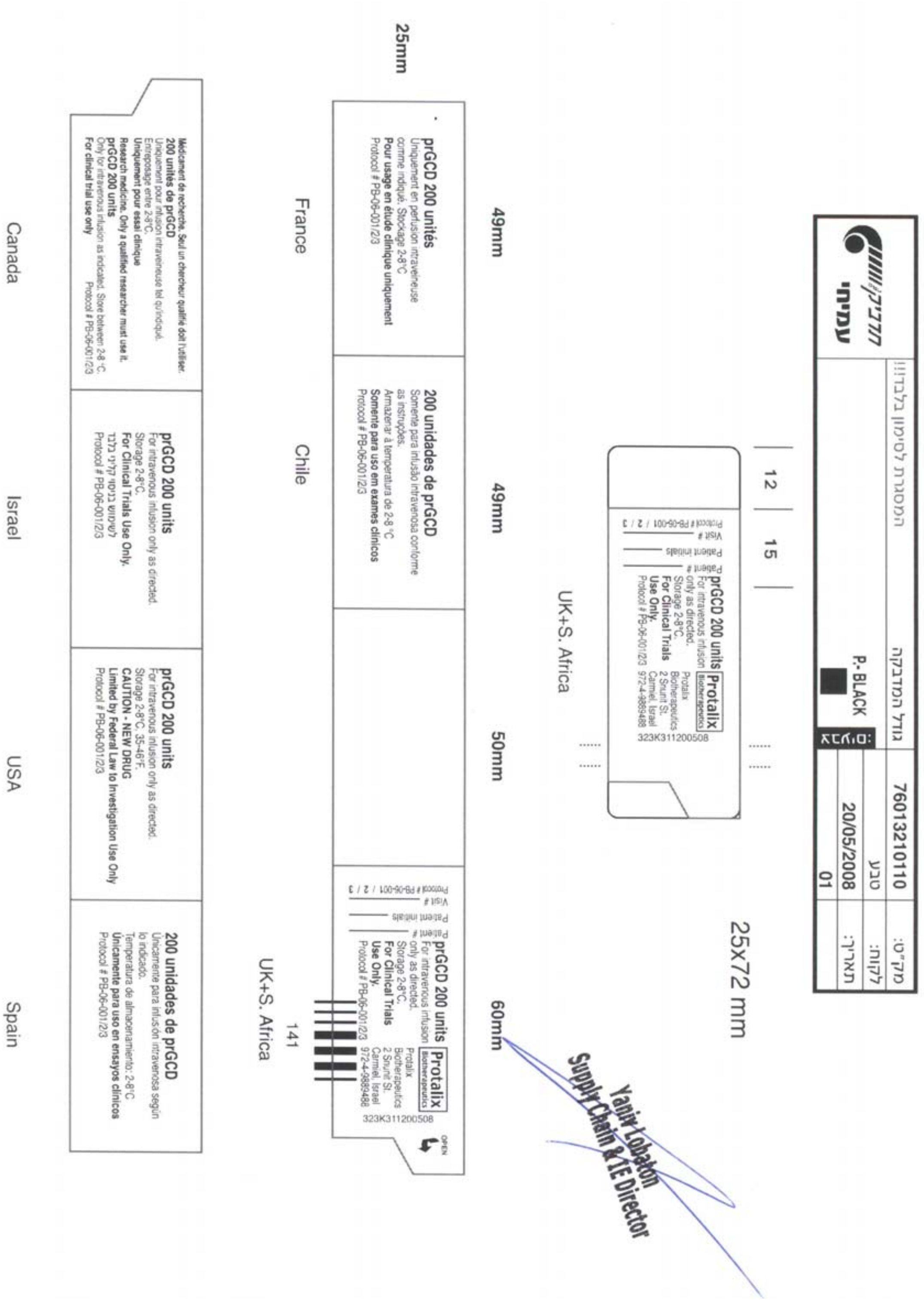

## 23.5. Appendix 5. Human prGCD Hypersensitivity Evaluation and Treatment Algorithm

During and after infusion of prGCD, the following algorithm will be followed to monitor and manage the occurrence of hypersensitivity, anaphylaxis, or anaphylactoid reactions.

**Clinical signs**

Early

- Sensation of warmth and itching
- Feelings of anxiety or panic

Moderate

- Pruritus
- Flushing
- Urticaria
- Chest discomfort
- Mild Hypotension

Progressive

- Erythematous or massive urticarial rash
- Edema of face, neck, soft tissues

Severe

- Hypotension
- Bronchospasm (wheezing)
- Laryngeal edema (dyspnea, stridor, aphonia, drooling)
- Arrhythmias

Treatment algorithm

With the onset of any of the above clinical signs, immediately discontinue study medication administration and initiate the following monitoring.

- Continuous electrocardiographic monitoring
- Continuous pulse oximetry
- Measure blood pressure every 5 minutes
- Perform chest auscultation every 5 minutes
- Blood samples need to be collected for Tryptase (29-33) and antibodies. Tryptase samples need to be withdrawn at:
    - 1$^{st}$ sample taken 0.25-3 hours after onset of symptoms
    - 2$^{nd}$ sample taken between 3-6 hours
    - 3$^{rd}$ sample taken 24-48 hours to verify the return to baseline.

In the case of progressive or severe hypersensitivity, treat appropriately and withdraw the patient from the study.

Treat as follows:

Urticaria or edema of the face, neck, or soft tissues

- Epinephrine 1:1000 solution, 0.5 mL subcutaneously, repeat as needed every 5-10 minutes
- Antihistamines
- Corticosteroids

Hypotension (systolic blood pressure (SBP) ≤ 90 mmHg)

- Isotonic sodium chloride solution, 1 L every 30 minutes as needed to maintain SBP > 90 mmHg
- Epinephrine 1:10,000 solution given IV at 1 µg/minute initially, then 2-10 µg/minute to maintain SBP > 90 mmHg
- Norepinephrine 4 mg in 1 L 5% dextrose in water given IV at 2-12 µg/min to maintain SBP > 90 mmHg
- Glucagon 1 mg in 1 L 5% dextrose in water give IV at 5-15 µg/minute for refractory hypotension

Bronchospasm

- Oxygen by face mask at 6-8 L/minute to maintain oxygen saturation at > 90%
- Epinephrine 1:1000 solution, 0.5 mL subcutaneously
- Albuterol 0.5 mL of 0.5% solution in 2.5 mL of sterile saline every 15 minutes up to three doses
- Inhaled beta-agonists
- Corticosteroids

Laryngeal edema

- Epinephrine 1:1000 solution, 0.5 mL subcutaneously, repeat as needed every 5 to 10 minutes
- Corticosteroids

**Premedication**

Premedication for subsequent prGCD infusions may be considered at the discretion of the investigator and Medical Director for patients experiencing early clinical signs of hypersensitivity or rash/urticaria that responds promptly to oral antihistamine administration (see also Appendix 2 for adjustment of infusion rate). The premedication will be applied according to the following steps as needed to prevent progressive hypersensitivity:

1. Antihistamine (H1 blocker: diphenhydramine, hydroxyzine, cetrizine, loratadine, desloratidine) at a standard dose 12 hours and 2 hours before the start of the infusion.

2. H1 blocker plus H2 blocker (ranitidine, cimetidine, famotidine) at standard doses 12 hours and 2 hours before the start of the infusion.

3. H1 blocker plus H2 blocker plus prednisone up to 50 mg administered 12 hours and 2 hours before the start of the infusion.

## 23.6. Appendix 6: WHO Common Toxicity Criteria

| Category | Toxicity | Grade0 | Grade1 | Grade2 | Grade3 | Grade4 |
|---|---|---|---|---|---|---|
| Haematology | WBC (x103/l) | 4 | 3.0 - 3.9 | 2.0 - 2.9 | 1.0 - 1.9 | < 1.0 |
| Haematology | Platelets (x103/l) | WNL | 75.0 - normal | 50.0 - 74.9 | 25.0 - 49.9 | < 25.0 |
| Haematology | Haemoglobin (g/dl) | WNL | 10.0 - normal | 8.0 - 9.9 | 6.5 - 7.9 | < 6.5 |
| Haematology | Granulocytes/ Bands (x103/l) | 2 | 1.5 - 1.9 | 1.0 - 1.4 | 0.5 - 0.9 | < 0.5 |
| Haematology | Lymphocytes (x103/l) | 2 | 1.5 - 1.9 | 1.0 - 1.4 | 0.5 - 0.9 | < 0.5 |
| Haematology | Haemorrhage | none | mild, no | gross, 1 - 2 units transfusion per episode | gross, 3 - 4 units transfusion per episode | massive, > 4 units transfusion per episode |
| Coagulation | Fibrinogen | WNL | 0.99 - 0.75 x N | 0.74 - 0.50 x N | 0.49 - 0.25 x N | < 0.25 x N |
| Coagulation | Prothrombin time(Quick) | WNL | 1.01 - 1.25 x N | 1.26 - 1.50 x N | 1.51 - 2.00 x N | > 2.00 x N |
| Coagulation | Partial thrombo-plastin time | WNL | 1.01 - 1.66 x N | 1.67 - 2.33 x N | 2.34 - 3.00 x N | > 3.00 x N |
| Metabolic | Hyperglycaemia (mg/dl) | < 116 | 116 - 160 | 161 - 250 | 251 - 500 | > 500 or ketoacidosis |
| Metabolic | Hypoglycaemia (mg/dl) | > 64 | 55 – 64 | 40 - 54 | 30 - 39 | < 30 |
| Metabolic | Amylase | WNL | < 1.5 x N | 1.5 - 2.0 x N | 2.1 - 5.0 N | > 5.0 x N |
| Metabolic | Hypercalcaemia (mg/dl) | < 10.6 | 10.6 - 11.5 | 11.6 - 12.5 | 12.6 - 13.4 | 13.5 |
| Metabolic | Hypocalcaemia (mg/dl) | > 8.4 | 8.4 - 7.8 | 7.7 - 7.0 | 6.9 - 6.1 | 6 |
| Metabolic | Hypomagnesaemia (mg/dl) | > 1.4 | 1.4 - 1.2 | 1.1 - 0.9 | 0.8 - 0.6 | 0.5 |
| Gastrointestinal | Nausea | none | able to eat reasonable intake | intake significantly decreased but can eat | no significant intake | — |

| Category | Toxicity | Grade0 | Grade1 | Grade2 | Grade3 | Grade4 |
|---|---|---|---|---|---|---|
| Gastrointestinal | Vomiting | none | 1 episode in 24 hrs | 2 - 5 episodes in 24 hrs | 6 - 10 episodes in 24 hrs | > 10 episodes in 24 hrs or requiring paren- teral support |
| Gastrointestinal | Diarrhoea | none | increase of 2 - 3 stools / day over pre-Rx | increase of 4 - 6 stools / day, or nocturnal stools, or moderate cramping | increase of 7 - 9 stools / day, or incontinence, or severe cramping | increase of > 10 stools / day or grossly bloody diarrhoea, or need for paren- teral support |
| Gastrointestinal | Stomatitis | none | painless ulcers, erythema, or mild soreness | painful erythema, oedema, or ulcers but can eat solids | painful erythema, oedema, or ulcers and cannot eat solids | requires parenteral or enteral support for alimentation |
| Liver | Bilirubin (N = 17 µmol/L) | WNL | ----- | < 1.5 x N | 1.5 - 3.0 x N | > 3.0 x N |
| Liver | Transaminase (SGOT, SGPT) | WNL | 2.5 x N | 2.6 - 5.0 x N | 5.1 - 20.0 x N | > 20.0 x N |
| Liver | Alk Phos or 5 nucleotidase | WNL | < 2.5 x N | 2.6 - 5.0 x N | 5.1 - 20.0 x N | > 20.0 x N |
| Liver | Liver- clinical | No change from baseline | ----- | ----- | precoma | hepatic coma |
| Kidney, bladder | Creatinine | WNL | < 1.5 x N | 1.5 - 3.0 x N | 3.1 - 6.0 x N | > 6.0 x N |
| Kidney, bladder | Proteinuria | No change | 1 (+) or < 0.3 g% or 3 g/L | 2 - 3 (+) or 0.3 - 1.0 g% or 3 - 10 g/L | 4 (+) or > 1.0 g% or > 10g/L | nephrotic syndrome |
| Kidney, bladder | Haematuria | Negative | microscopic only | gross, no clots no Rx needed | gross and clots bladder irrigation | requires trans- fusion or cystectomy |
| Kidney, bladder | Weight gain/ loss | < 5.0 % | 5.0 - 9.9 % | 10.0 - 19.9 % | 20.00% | ----- |
| Pulmonary | Pulmonary | none or no change | asymptomatic, with abnormal- ity in PFTs | dyspnoea on significant exertion | dyspnoea at normal level of activity | dyspnoea at rest |

| Category | Toxicity | Grade0 | Grade1 | Grade2 | Grade3 | Grade4 |
|---|---|---|---|---|---|---|
| Cardiac | Cardiac arrhythmias | none | asymptomatic, transient, requiring no therapy | recurrent or persistent, no therapy required | requires treatment | requires monitoring; or hypotension, or ventricular tachycardia or fibrillation |
| Cardiac | Cardiac function | none | asymptomatic, decline of resting ejection fraction by less than 20 % of baseline value | asymptomatic, decline of resting ejection fraction by more than 20 % of baseline value | mild CHF, responsive to therapy | severe of refractory CHF |
| Cardiac | Cardiac ischaemia | none | non-specific T- wave flattening | asymptomatic, ST and T wave changes suggesting ischaemia | angina without evidence of infraction | acute myocardial infarction |
| Cardiac | Cardiac- pericardial | none | asymptomatic effusion, no intervention required | pericarditis (rub, chest pain, ECG changes) | symptomatic effusion; drainage required | tamponade; drainage urgently required |
| Cardiac | Hypertension | none or no change | asymptomatic, transient increase by greater than 20 mm Hg (D) or to > 150 / 100 if previously WNL. No treatment required. | recurrent or persistent increase by greater than 20 mm HG (D) or to > 150 / 100 if previously WNL. No treatment required. | requires therapy | hypertensive crisis |
| Cardiac | Hypotension | none or no change | changes requiring no therapy (including transient orthostatic hypotension) | requires fluid replacement or other therapy but not hospitalisation | requires therapy and hospitalisation; resolves within 48 hours of stopping the agent | requires therapy and hospitalisation for > 48 hrs after stopping the agent |
| Neurologic | Neuro: sensory | none or no change | mild paraesthesias; loss of deep tendon reflexes | mild or moderate objective sensory loss moderate paraesthesias | severe objective sensory loss or paraesthesias that interfere with function | ----- |
| Neurologic | Neuro: motor | none or no change | subjective weakness; no objective findings | mild objective weakness without significant impairment of function | objective weakness with impairment of function | paralysis |

| Category | Toxicity | Grade0 | Grade1 | Grade2 | Grade3 | Grade4 |
|---|---|---|---|---|---|---|
| Neurologic | Neuro: cortical | none | mild somnolence or agitation | moderate somnolence or agitation | severe somnolence, (>50 % waking hours), agitation, confusion, disorientation or hallucinations | coma, seizures, toxic psychosis |
| Neurologic | Neuro: cerebellar | none | slight incoordination, dysdiadochokinesia | intention tremor, dysmetria, slurred speech, nystagmus | locomotor ataxia | cerebellar necrosis |
| Neurologic | Neuro: mood | no change | mild anxiety or depression | moderate anxiety or depression | severe anxiety or depression | suicidal ideation |
| Neurologic | Neuro: headache | none | mild | moderate or severe but transient | unrelenting and severe | ----- |
| Neurologic | Neuro: constipation | none or no change | mild | moderate | severe | ileus > 96 hrs |
| Neurologic | Neuro: hearing | none or no change | asymptomatic, hearing loss on audiometry only | tinnitus | hearing loss interfering with function but correctable with hearing aid | deafness not correctable |
| Neurologic | Neuro: vision | none or no change | ----- | ----- | symptomatic subtotal loss of vision | blindness |
| Pain | Pain | none | mild | moderate | severe | requires narcotics |
| Skin | Skin | none or no change | scattered macular ot papular eruption or erythema that is asymptomatic | scattered macular or papular eruption or erythema with pruritus or other associated symptoms | generalised symptomatic macular, papular or vesicular eruption | exfoliative dermatitis or ulcerating dermatitis |
| Alopecia | Alopecia | no loss | mild hair loss | pronounced or total hair loss | ----- | ----- |
| Allergy | Allergy | none | transient rash, drug fever < 38°C (100.4°F) | urticaria, drug fever 38°C (100.4°F), mild bronchospasm | serum sickness, bronchospasm requiring parenteral medication | anaphylaxis |

| Category | Toxicity | Grade0 | Grade1 | Grade2 | Grade3 | Grade4 |
|---|---|---|---|---|---|---|
| Local | Local | none | pain | pain and swelling with inflammation or phlebitis | ulceration | plastic surgery indicated |
| Fever of unknown origin | Fever of unknown origin | none | 37.1 - 38.0° C 98.7° - 100.4° F | 38.1 - 40.0°C 100.5 - 104°F | > 40.0°C > 104.0°F for less than 24hrs | > 40.0°C (>104°F) for more than 24 hrs or accompanied by hypotension |
| Infection | Infection | none | mild | moderate | severe | life-threatening |
| Additional events | Asthenia | Analogous to Karnofsky index (WHO grading) | | | | |
| Additional events | Chills | Analogous to fever | | | | |
| Additional events | Peripheral oedema | analogous to weight gain | | | | |
| Additional events | Anorexia | analogous to weight loss | | | | |
