# Supplementary material for: Immunogenicity of glycans on biotherapeutic drugs produced in plant expression systems—The taliglucerase alfa story
Source: PLoS One. 2017 Oct 31;12(10):e0186211. doi: 10.1371/journal.pone.0186211 (PMC5663370; doi:10.1371/journal.pone.0186211)
Supplement: S3 File — (PDF) [file pone.0186211.s003.pdf]

CLINICAL STUDY PROTOCOL

Protocol Title: A Multicenter, Double-blind, Randomized Safety and Efficacy Study of Two Dose Levels of Taliglucerase alfa in Pediatric Subjects with Gaucher Disease

Protocol Number: PB-06-005

**Study Product:**	Taliglucerase alfa (plant cell expressed recombinant human glucocerebrosidase)
**Indication:**	Gaucher Disease
**Phase:**	3B
**Name and Affiliation of Principal Investigator:**	A list of the Principal Investigators is maintained in the trial master file
**Name and Address of Sponsor:**	Protalix Ltd.<br>2 Snunit Street<br>Science Park<br>POB 455<br>Carmiel 20100, Israel
**GCP Statement:**	This study will be performed in compliance with GCP, including the archiving of essential documents.

The information in this document is confidential and is proprietary to Protalix, Ltd. It is understood that information in this document shall not be disclosed to any third party, in any form, without prior written consent of Protalix.

1 SYNOPSIS

TITLE: A Multicenter, Double-blind, Randomized Safety and Efficacy Study of Two Dose Levels of Taliglucerase alfa in Pediatric Subjects with Gaucher Disease

INVESTIGATIONAL PRODUCT: Taliglucerase alfa (plant cell expressed recombinant human glucocerebrosidase)

INDICATION: Gaucher Disease

PHASE OF DEVELOPMENT: 3B

INVESTIGATIONAL SITES/LOCATIONS: Multicenter, to be determined

OBJECTIVES: To assess the safety and efficacy of taliglucerase alfa in untreated subjects (2 to <18 years old) with symptoms and clinical manifestations of Gaucher disease

STUDY DESIGN: This is a multi-center, double-blind trial to assess the safety and efficacy of taliglucerase alfa in 10 untreated subjects (2 to <18 years old) with Gaucher disease randomly assigned to treatment with one of two doses, 30 or 60 units/kg. Subjects will receive an intravenous (IV) infusion of taliglucerase alfa every two weeks. The total duration of treatment will be 12 months. At the end of the 12-month treatment period eligible subjects will be offered enrollment in an open-label extension study if taliglucerase alfa is not commercially available.

NUMBER OF SUBJECTS (PLANNED): 10 subjects

DIAGNOSIS AND MAIN CRITERIA FOR INCLUSION:

Key inclusion criteria:

Eligible subjects must fulfill the following inclusion criteria:

1. Males and females 2 to <18 years old.
2. Diagnosis of Gaucher disease with leukocyte acid β-glucosidase activity ≤30% of the mean of the reference range for healthy subjects.
3. Subjects who have not received enzyme replacement therapy (ERT) in the past or who have not received ERT in the past 12 months and have a negative anti-glucocerebrosidase antibody assay.
4. Subjects who have not received substrate reduction therapy (SRT) in the past 12 months.
5. Subjects whose clinical condition, in the opinion of the investigator, requires treatment with enzyme replacement therapy (ERT).

Key exclusion criteria:

Eligible subjects may not have any of the following exclusion criteria:

1. Currently taking another investigational drug for any condition.
2. Presence of neurological signs and symptoms characteristic of Gaucher disease with complex neuronopathic features other than longstanding oculomotor gaze palsy.
3. Presence of unresolved anemia due to iron, folic acid, or vitamin B12 deficiency
4. Previous hypersensitivity reaction to Cerezyme® (imiglucerase) or Ceredase® (alglucerase).

5. History of allergy to carrots.
6. Presence of HIV, HBsAg or hepatitis C infections.
7. Subject's parent(s) or legal guardian(s) are unable to understand the nature, scope and possible consequences of the study.
8. Presence of any medical, emotional, behavioral or psychological condition that in the judgment of the Investigator would interfere with the subject's compliance with the requirements of the study.

(See body of protocol for full list of exclusions)

TEST PRODUCT(S), DOSE AND MODE OF ADMINISTRATION: **T**aliglucerase alfa, 30 or 60 units/kg, intravenously, every 2 weeks

DURATION OF TREATMENT: 12 months

DISCONTINUATION FROM TREATMENT:
Reasons for permanent discontinuation include the following:

- The subject experiences two or more Grade 3 toxicities or one or more Grade 4 toxicity considered by the investigator associated with taliglucerase alfa treatment
- The subject who experiences progressive hypersensitivity or severe hypersensitivity are to be treated appropriately and withdrawn from the study
- The subject, or subject's parent or guardian, requests to discontinue treatment
- Investigator feels that it is not in the best interest of the subject to continue treatment and/or if the investigator believes that the subject can no longer be compliant with the requirements of the study

EFFICACY VARIABLES:

- The primary efficacy variable is median percentage and the interquartile range for change from baseline in haemoglobin
- Secondary efficacy variables are:
  - Percent change from baseline in chitotriosidase or CCL18
  - Percent change from baseline in spleen and liver volume
  - Percent change from baseline in platelet count

EXPLORATORY ENDPOINTS:

- Growth and development (height, weight, sexual development by Tanner classification, bone age by X-ray of left hand and wrist)
- Bone disease (Change from baseline to end of study of lumbar spine area and/or volumetric bone mineral density z score (DEXA), occurrence of bone crises)
- Change in Quality of Life

SAFETY ENDPOINTS:

- Clinical laboratory
- Echocardiography
- Adverse events
- Anti-taliglucerase alfa antibodies

STATISTICAL ANALYSIS:

DOCUMENT APPROVAL

This study will be conducted in compliance with the protocol, Good Clinical Practice (GCP) and applicable regulatory requirements.

SPONSOR REPRESENTATIVE

Einat Almon

____ _____28.4.10____

Signature Date

Einat Almon, Ph.D Sr. VP Product Development

PRINCIPAL INVESTIGATOR

____ ________________________

Signature Date

Print Name: _________________________________

TABLE OF CONTENTS

1 SYNOPSIS 2
2 LIST OF ABBREVIATIONS 8
3 ETHICAL CONDUCT OF THE STUDY AND REGULATORY REQUIREMENTS 9
3.1 Institutional Review Board (IRB) 9
3.2 Ethical Conduct of the Study 9
3.3 Subject Information and Consent 9
4 INTRODUCTION 10
5 STUDY OBJECTIVES 13
6 INVESTIGATIONAL PLAN 13
6.1 Overall Study Design and Plan – Description 13
6.2 Discussion of Study Design and Choice of Control Group(s) 13
6.3 Selection of Study Population 14
6.3.1 Inclusion Criteria 14
6.3.2 Exclusion Criteria 14
6.3.3 Removal of Subjects from Therapy or Assessment 14
6.3.4 Replacement Policy 15
7 STUDY PRODUCT 16
7.1 Study Medication Supply 16
7.2 Description of Study Product 16
7.3 Description of Comparator Product 16
7.4 Study Drug Administration 16
7.5 Packaging and Labeling 16
7.6 Conditions for Storage and Use 16
7.7 Method of Assigning Subjects to Treatment Groups 17
7.8 Dispensing, Compliance and Accountability 17
7.9 Prior and Concomitant Therapy 17
8 EFFICACY AND SAFETY ASSESSMENTS 18
8.1 Efficacy Variable(s) 18
8.2 Safety Variables 18
8.2.1 Clinical Laboratory 18
8.2.2 Echocardiography 18
8.2.3 Anti-Taliglucerase alfa Antibodies 19
8.2.4 Adverse Events 19
9 STUDY PROCEDURES AND FLOW CHART 22
9.1 Study Flow Chart 23
9.2 Study Visits 24
9.2.1 Screening (Visit 0, Day -25 ± 10) 24
9.2.2 Visit 1 (Baseline, Day 1) 24
9.2.3 Visits 2 – 6 (Weeks 2, 4, 6, 8, 10 ± 3 Days) 25
9.2.4 Visit 7 (Month 3, Week 12 ± 7 Days) 25

9.2.5 Visits 8 – 13 (Weeks 14, 16, 18, 20, 22, 24 ± 3 days)....26
9.2.6 Visit 14 (Month 6, Week 26 ± 7 days)....26
9.2.7 Visit 15 – 19 (Weeks 28, 30, 32, 34, 36 ± 3 days)....27
9.2.8 Visit 20 (Month 9, Week 38 ± 7 days)....27
9.2.9 Visit 21 – 27 (Weeks 40, 42, 44, 46, 48, 50 ± 3 days)....27
9.2.10 Visit 28 (Month 12, Week 52 ± 7 days)....28
10 STATISTICAL METHODS PLANNED AND SAMPLE SIZE....29
10.1 Determination of Sample Size....29
10.2 Subject Populations....29
10.3 Analysis....29
10.4 Safety Analysis....29
10.4.1 Adverse Events....30
10.4.2 Clinical Laboratory....30
11 QUALITY CONTROL AND QUALITY ASSURANCE....31
11.1 Source Data and Records....31
11.2 Reporting of Results....31
11.3 Confidentiality of Subject Data....32
12 REPORTING AND PUBLICATION....33
12.1 Confidentiality of Study Data....33
12.2 Publication Policy....33
13 Appendices....34
13.1 Appendix 1. Vial Label....34
13.2 Appendix 2. Infusion Rate Algorithm....35
13.3 Appendix 3. Taliglucerase alfa Hypersensitivity Evaluation and Treatment Algorithm.37
13.4 Appendix 4. MRI....40
13.4.1 Subjects and sites....40
13.4.2 Magnetic Resonance Imaging (MRI) data....40
13.4.3 MRI evaluation parameters....40
13.4.4 Sites and image data management....40
13.4.5 Image processing and centralized analysis....41
13.4.6 Data and report transfers to Sponsor....42
13.4.7 Direct access to Study data....42
13.4.8 Unevaluable MRI:....43
13.5 Appendix 5: WHO Common Toxicity Criteria....44

2 LIST OF ABBREVIATIONS

AE	Adverse event
CCL18	Pulmonary and activation-regulated chemokine
CHQ	Child Health Questionnaire
CRF	Case report form
EC	Ethics Committee
ERT	Enzyme replacement therapy
GCP	Good Clinical Practice
hGCD	Human glucocerebrosidase
IRB	Institutional Review Board
MRI	Magnetic resonance imaging
SAE	Serious adverse event
SRT	Subtrate reduction therapy
TSH	Thyroid stimulating hormone

3 ETHICAL CONDUCT OF THE STUDY AND REGULATORY REQUIREMENTS

3.1 Institutional Review Board (IRB)

An Institutional Review Board (IRB) or Ethics Committee (EC) will review the study protocol and any amendments. The IRB or EC will also review the informed consent form, their updates (if any), and any written materials given to the subjects. A list of all IRBs and ECs and contact information will be included in the study report.

3.2 Ethical Conduct of the Study

This study will be conducted in accordance with the ethical principles that have their origins in the Declaration of Helsinki, in compliance with the approved protocol, GCP and applicable regulatory requirements.

3.3 Subject Information and Consent

The investigator will obtain a freely given written consent or assent from each subject or parent or guardian after an appropriate explanation of the aims, methods, anticipated benefits, potential hazards, and any other aspects of the study that are relevant to the subject's decision to participate. The consent form must be signed and dated by the subject or parent or guardian before he/she is exposed to any protocol-specific procedure.

The investigator will explain that the subjects are completely free to refuse to enter the study or to withdraw from it at any time, without any consequences for their further care and without the need to justify.

The subject will receive a copy of the subject information and the signed informed consent.

The subject will be informed if information becomes available that may be relevant to his/her willingness to continue participation in the study.

Each subject will be informed that a monitor or a health authority inspector, in accordance with applicable regulatory requirements, may review the portions of their source records and source data related to the study. Data protection and confidentiality will be handled in compliance with local laws.

4 INTRODUCTION

Gaucher disease is a lipid storage disease characterized by the deposition of glucocerebroside in cells of the macrophage-monocyte system. The disorder results from the deficiency of a specific lysosomal hydrolase, glucocerebrosidase (also termed acid β-glucosidase, glucosylceramidase). The disease is characterized by a continuum of phenotypes. The severity widely varies; some patients present in childhood with virtually all the complications of Gaucher disease, whereas others remain asymptomatic into the eighth decade of life.

Glucosylceramide, the accumulated glycolipid, is primarily derived from the phagocytosis and degradation of senescent leukocytes and, to a lesser extent, from erythrocyte membranes. The glycolipid storage gives rise to the characteristic Gaucher cells, macrophages engorged with lipid with a crumpled–tissue-paper appearance and displaced nuclei.

Glucosylceramide accumulation in the bone marrow, liver, spleen, lungs, and other organs contributes to pancytopenia, massive hepatosplenomegaly, and, at times, diffuse infiltrative pulmonary disease. Progressive infiltration of Gaucher cells in the bone marrow may lead to thinning of the cortex, pathologic fractures, bone pain, bony infarcts, and osteopenia. These bony features may also be related to macrophage-produced cytokines.

Enzyme replacement therapy (ERT) for Gaucher disease is available, with most patients receiving recombinant enzyme (imiglucerase [Cerezyme®]). ERT is highly effective in reversing the visceral and hematologic manifestations of Gaucher disease.

ERT is indicated for patients with Gaucher disease who exhibit clinical signs and symptoms of the disease, including anemia, thrombocytopenia, skeletal disease, or visceromegaly. ERT is not effective in neuronopathic forms of the disease. Severity and rate of disease progression widely varies, especially in adults, which makes treatment decisions extremely difficult in some patients. Generally, subjects who present symptomatically, rather than because of family history, may have severe disease manifestations that require early treatment. Presymptomatic treatment with imiglucerase remains controversial because of the lack of prognostic correlation between genotype and disease severity and the high cost of the therapy.

Unmodified human glucocerebrosidase (hGCD), derived from natural sources, cannot be targeted to the phagocytic cells in the body and is, therefore, of limited therapeutic value. In developing the current therapeutic products for Gaucher disease, the terminal sugars on the carbohydrate chains of hGCD are sequentially removed by treatment with three different glycosidases. This glycosidase treatment results in a glycoprotein whose terminal sugars consist of mannose residues. This facilitates uptake by mannose receptors on phagocyte cells that recognize glycoproteins and glycopeptides with oligosaccharide chains that terminate in mannose residues. The carbohydrate remodeling of hGCD improves the targeting of the enzyme to these cells.

Since the glycosylation pattern of hGCD must be remodeled to generate high mannose structures to increase uptake in target cells, the expression of hGCD in plant cells could be of great value. Post-translational modifications do not exist in bacterial expression systems, but plant derived expression systems do facilitate these modifications known to be crucial for protein expression and activity. One of the major differences between mammalian and plant protein expression systems is the variation of protein sugar side chains, caused by the differences in biosynthetic pathways. Glycosylation has been shown to have a profound effect on activity, folding, stability, solubility, and susceptibility to proteases, blood clearance rate and antigenic potential of proteins. Hence, any protein production in plants should take into consideration the potential ramifications of plant glycosylation.

The production of taliglucerase alfa utilizes Agrobacterium tumefacience, a bacterium capable of inserting single stranded DNA molecules into the plant genome. Due to the relative simplicity of introducing genes for mass production of proteins and peptides, this methodology is becoming increasingly popular as an alternative protein expression system.

The plant cell expressed recombinant hGCD (taliglucerase alfa) is similar to the human enzyme, β-glucocerebrosidase (β-D-glucosyl-N-acylsphingosine glucohydrolase, E.C. 3.2.1.45), a lysosomal glycoprotein enzyme, which catalyses the hydrolysis of the glycolipid glucocerebroside to glucose and ceramide. The amino acid sequence of the taliglucerase alfa has two extra amino acids (E and F) at the N-terminal and additional 7 amino acids at the C-terminal (DLLVDTM) flanking the core amino acid sequence of the human enzyme. These extra amino acids allow for specific targeting of Gaucher cells.

It was demonstrated in non-clinical studies that taliglucerase alfa uptake is specifically mediated by mannose receptors on macrophages and that, once in the macrophage, the enzyme is active on its substrate. Moreover, in the pivotal clinical trial (PB-06-001), efficacy of taliglucerase alfa for the treatment of Gaucher disease has been demonstrated.

To address the safety of taliglucerase alfa, Protalix performed single dose toxicity studies in the mouse and cynomolgus monkey, repeat dose studies (7, 14, and 29 days) in the marmoset monkey, repeat dose studies (28 days and 39 weeks) in the cynomolgus monkey, and Segment I and II reproductive toxicity studies in the rat and rabbit. Following single or repeated IV dosing, minimal to no systemic toxicity was observed. Effects were largely limited to the site of injection; the changes were related to the IV dosing technique and not to taliglucerase alfa. The drug did not adversely affect fertility and did not produce developmental malformations.

Taliglucerase alfa was evaluated for safety and pharmacokinetics in a Phase 1 study performed in six healthy subjects (P-01-2005) and for safety, efficacy and pharmacokinetics in a single pivotal, Phase 3 study performed in 32 patients with Gaucher disease (PB-06-001). The pivotal study was a multicenter, double blind, randomized, parallel group, dose ranging study in which

taliglucerase alfa was administered as an intravenous infusion every two weeks for 38 weeks (20 infusions). Patients were randomized to receive either 30 units/kg or 60 units/kg. Patients were enrolled in 11 centers around the world.

The primary efficacy analysis in study PB-06-001 demonstrated that taliglucerase alfa treatment significantly reduced spleen volume from screening to the Month 9 timepoint (taliglucerase alfa 30 units/kg, 26.91%; taliglucerase alfa 60 units/kg, 38.01%; $p<0.0001$) and at the Month 6 timepoint (taliglucerase alfa 30 units/kg, 22.21%; taliglucerase alfa 60 units/kg, 29.94%; $p<0.0001$) in patients with Gaucher disease.

The secondary efficacy analysis demonstrated that taliglucerase alfa treatment significantly increased hemoglobin level from the baseline at Month 9 timepoint (taliglucerase alfa 30 units/kg, 1.6 g/dL; taliglucerase alfa 60 units/kg, 2.2 g/dL; $p<0.0001$). A significant reduction in liver volume from screening was also observed in both taliglucerase alfa dose groups at the end of the study (taliglucerase alfa 30 units/kg, 10.48%, $P=0.0041$; taliglucerase alfa 60 units/kg, 11.11%, $p<0.0001$). In addition, a significant increase in platelet count from baseline was observed in the 60 units/kg dose group at Month 9 (41,494/mm^3, $p=0.0031$) and a clinically relevant improvement in platelet count at Month 9 was also observed for the taliglucerase alfa 30 units/kg dose group (11,427/mm^3, $P=0.0460$). Improvements were also observed at the 6 month visit. At the end of study, all taliglucerase alfa treated patients had at least a 10% reduction in spleen volume.

Overall in PB-06-001, 23 taliglucerase alfa treated patients (30 units/kg, 12; 60 units/kg, 11) experienced 137 AEs (30 units/kg, 65; 60 units/kg, 72). Eight of these patients (30 units/kg, 3; 60 units/kg, 5) experienced 28 events (30 units/kg, 12; 60 units/kg, 16) which were considered by the investigator treatment related. All AEs were mild or moderate in intensity and the majority of the events resolved by the end of the study. No deaths or SAEs occurred during the study. Two patients (taliglucerase alfa 30 units/kg, 10-003; taliglucerase alfa 60 units/kg, 10-002) discontinued from the study due to a hypersensitivity reaction.

Adverse events judged to be related to treatment with taliglucerase alfa and occurring at an increased frequency were hypersensitivity, headache, and pruritis. These events occurred in a similar frequency in the 30 and 60 unit/kg dose groups. Other reported adverse events are abdominal pain, feeling hot, arthralgia, muscle spasms, dizziness, glycosuria and skin irritation.

A second clinical study (PB-06-002) has been initiated in patients with Gaucher disease and is ongoing. This study is an open label study in which patients previously treated with Cerezyme® (imiglucerase) are switched over to the same dose of taliglucerase alfa after establishing that their disease control is stable. Patients are being treated with taliglucerase alfa every 2 weeks for 9 months. Subjects aged 2 to < 18 years are eligible for enrolment in this study.

Study PB-06-003 is an extension study enrolling patients who have completed PB-06-001 and PB-06-002 and is ongoing. The patients continue to receive the same dose of taliglucerase alfa for an additional period of 15 or more months.

Study PB-06-004 is a treatment protocol enrolling patients who were previously treated with Cerezyme®, but who were put on a reduced dose regimen or discontinued because of a shortage of the drug. The protocol has been initiated and is ongoing.

5 STUDY OBJECTIVES

The efficacy objectives of this trial are to assess the efficacy of taliglucerase alfa in subjects between 2 to < 18 years old with Gaucher disease, as measured by percent change in haemoglobin, chitotriosidase or CCL18, spleen and liver volume evaluated by MRI (or ultrasound), and platelet count, and change in growth and development (height, weight, Tanner Stage, bone age), bone disease, and Quality of Life from baseline.

The safety objectives are to assess the safety of taliglucerase alfa by clinical laboratory, physical examination, echocardiography, adverse events and anti-taliglucerase alfa antibodies

6 INVESTIGATIONAL PLAN

6.1 Overall Study Design and Plan – Description

This will be a multi-center, double-blind trial to assess the efficacy and safety of taliglucerase alfa in 10 untreated subjects between 2 to < 18 years old with Gaucher disease randomized to either 30 or 60 units/kg. Subjects will receive intravenous infusion of taliglucerase alfa every two weeks for 12 months. Subjects who tolerate the infusions well for three months will be eligible for home therapy based on approval of the investigator and the Medical Director. The total duration of treatment will be 12 months. At the end of the 12-month treatment period eligible subjects will be offered enrollment in an open-label extension study if taliglucerase alfa is not commercially available.

6.2 Discussion of Study Design and Choice of Control Group(s)

This study represents one of the first pediatric exposures to taliglucerase alfa. Subjects will have a definitive diagnosis of Gaucher disease based on glucocerebrosidase activity with significant manifestations of the disease not previously treated with enzyme replacement therapy, or not treated in the last 12 months. This study presents an opportunity to assess the efficacy of taliglucerase alfa in a disease that does not improve spontaneously in this population. The parameters chosen as endpoints for this study are the most relevant parameters to the disease and allow a significant and relevant evaluation of improvement as a result of treatment for 12 months. Improvement in the clinical manifestations of the disease leads to the achievement of the therapeutic goals of Gaucher disease treatment in the medical community, which are enhanced quality of life and decreased risk for morbidities.

The study of two dose levels may provide clinically important information on whether there is a difference in treatment effect or a difference in rate of onset of treatment effect.

6.3 Selection of Study Population

6.3.1 Inclusion Criteria

The subjects must meet the following inclusion criteria:

1. Male and female 2 years and <18 years old.
2. Diagnosis of Gaucher disease with leukocyte acid β-glucosidase activity ≤30% of the mean of the reference range for healthy subjects.
3. Subjects who have not received enzyme replacement therapy in the past or who have not received ERT in the past 12 months and have a negative anti-glucocerebosidase assay assessment.
4. Subjects who have not received substrate reduction therapy (SRT) in the past 12 months.
5. Subjects whose clinical condition, in the opinion of the investigator, requires treatment with enzyme replacement therapy (ERT)

6.3.2 Exclusion Criteria

The presence of any of the following excludes a subject from study enrollment:

1. Subjects currently taking another investigational drug for any condition.
2. The presence of neurological signs and symptoms characteristic of Gaucher disease with complex neuronopathic features other than longstanding oculomotor gaze palsy.
3. Presence of unresolved anemia due to iron, folic acid, or vitamin B12 deficiency.
4. Previous hypersensitivity reaction to Cerezyme® (imiglucerase) or Ceredase® (alglucerase).
5. History of allergy to carrots.
6. The presence of HIV, HBsAg or hepatitis C infections.
7. The subject's parent(s) or legal guardian(s) are unable to understand the nature, scope and possible consequences of the study.
8. The presence of a medical, emotional, behavioral or psychological condition that, in the judgment of the Investigator, would interfere with the subject's compliance with the requirements of the study.

6.3.3 Removal of Subjects from Therapy or Assessment

Reasons for permanent discontinuation include the following:

- The subject experiences two or more Grade 3 toxicities or one or more Grade 4 toxicity considered by the investigator associated with taliglucerase alfa treatment (see Sections 8.2.4 Adverse Events and 13.5, Appendix 5 WHO Common Toxicity Criteria)

- Subjects who experience progressive hypersensitivity or severe hypersensitivity will be treated appropriately and withdrawn from the study (see Appendix 2 and Appendix 3)
- The subject requests to discontinue treatment
- Investigator feels that it is not in the best interest of the subject to continue treatment and/or if the investigator believes the subject can no longer be compliant with the requirements of the study.

For any discontinuation, the Investigator will obtain all the required details and document the date and the main reason for the premature termination. If the reason for discontinuation is an adverse event, the specific event or the main laboratory abnormality will be recorded in the CRF. The Investigator will make thorough efforts to document the outcome. The Investigator will attempt to continue to follow the subject for the full duration of the study or at least for 30 days following discontinuation. If circumstances prevent the subject from completing all visits, every attempt will be made to complete all procedures listed in Section 9 for Visit 28.

6.3.4 Replacement Policy

Withdrawn subjects will not be replaced.

7 STUDY PRODUCT

7.1 Study Medication Supply

Reconstitution of each taliglucerase alfa vial (212 units) with sterile water for injection (5.1 mL) yields a final volume of 5.3 mL human taliglucerase alfa (40 U/mL), which provides a withdrawal volume of 5.0 mL (200 units). The solution must be mixed gently until clear.

7.2 Description of Study Product

Human taliglucerase alfa is a purified recombinant, plant cells-expressed glucocerebrosidase, which is described in detail in the Investigator Brochure.

Each 200 unit vial contains the following lyophilized contents:

212 units of taliglucerase alfa

195 mg mannitol

35 mg sodium citrate

0.53 mg polysorbate 80, NF

7.3 Description of Comparator Product

Not applicable.

7.4 Study Drug Administration

For initial infusions in all subjects, taliglucerase alfa will be administered at a rate of 1.2 mL/min over 2 hours. The tolerability of the infusion will be determined by signs and symptoms during the infusion for one hour after the infusion in the clinic and by telephone contact the day after the infusion. If this rate of infusion is well tolerated, the rate of infusion may be increased up to 2.25 mL/min to deliver the 135 mL volume over one hour for all subsequent dosing after discussion with the Medical Director. The infusion rate may be adjusted according to individual subject symptoms and signs (see Section 13.2, Appendix 2).

7.5 Packaging and Labeling

Lyophilized drug powder is stored in 13.5 mL borosilicate glass (Type 1) bottles (Forma Vitrum AG, Hungary). Lyophilization stoppers (Helvoet Pharma, Belgium) composed of two-leg brombutyl rubber are sealed with aluminum Snap Caps and polypropylen discs (Helvoet Pharma, Belgium).

The label is in Appendix 1.

7.6 Conditions for Storage and Use

The product is stored at 2-8°C (36-46°F).

7.7 Method of Assigning Subjects to Treatment Groups

This is a randomized study and all subjects will be assigned to treatment with taliglucerase alfa 30 or 60 units/kg. A unique screening number (formatted as xxS5yyy [x: site number, 5: protocol number, y: subject number]) will be assigned to each screened subject. Once a subject is eligible for randomization, including successful completion of screening and prior to visit 1, a subject randomization number will be generated that will include the site and subject number (formatted as xx-5yyy [x: site number, 5: protocol number, y: randomization number]). Subjects will be randomized to one of the two treatment groups (30 units/kg or 60 units/kg) based on a centralized computer-generated randomization code.

7.8 Dispensing, Compliance and Accountability

Protalix will provide drug accountability forms to assist the pharmacist in maintaining current and accurate inventory records covering receipt, dispensing, and the return of investigational drug supplies. When a shipment is received, the pharmacist will verify the quantities received and return the acknowledgment to the study monitor or designee. The pharmacist investigational drug accountability record includes the identification of the person to whom the drug is dispensed, the quantity and the date of dispensing and any returned or unused drug. This record is in addition to any drug accountability information recorded on the Case Report Form (CRF). These records will be readily available for inspection by a monitor or Protalix audits and are open to regulatory authority inspection at any time.

The investigator is responsible for maintaining accountability for the receipt, dispensing, and return of all study medication.

7.9 Prior and Concomitant Therapy

Medications having the potential to interfere with the evaluation of efficacy are excluded throughout the trial.

The following medications are strictly prohibited during the study:

- Zavesca® (miglustat)
- Ceredase® (algucerase)
- Cerezyme® (imiglucerase)

The following medications are allowed and expected during the study:

- Treatments for hypersensitivity, anaphylaxis, or anaphylactoid reactions; e.g., epinephrine, norepinephrine, glucagons, albuterol
- Treatments for anemia; e.g., iron, folic acid, vitamin B12
- Treatments for bone disease; e.g., biphosphonates

- Analgesics; e.g., nonsteroidal anti-inflammatory drugs (note: aspirin may be contraindicated due to its effects on platelets and risk for bleeding)

8 EFFICACY AND SAFETY ASSESSMENTS

8.1 Efficacy Variable(s)

- The primary efficacy variable is median percentage and the interquartile range for change from baseline in haemoglobin
- Secondary efficacy variables are:
  - Percent change from baseline in chitotriosidase or CCL18
  - Percent change from baseline in spleen and liver volume (see Appendix 4)
  - Percent change from baseline in platelet count
- Exploratory endpoints are:
  - Change in height and weight
  - Change in Tanner Stage
  - Change in bone age by X-ray of left hand and wrist
  - Change in bone density by DEXA
  - Occurrence of bone crises
  - Qualify of Life using the Child Health Questionnaire™ (CHQ) PF-28 (valid for subjects aged 5 to 18 years)

8.2 Safety Variables

The safety of taliglucerase alfa will be assessed by clinical laboratory, echocardiography, anti-taliglucerase alfa antibodies, and adverse events.

8.2.1 Clinical Laboratory

- Haematology: complete blood count; total white blood cell count, differential count (neutrophils, lymphocytes, monocytes, eosinophils and basophils), red blood cells, (hemoglobin, hematocrit, mean corpuscular volume, mean corpuscular hemoglobin and mean corpuscular hemoglobin concentration), and platelets
- Biochemistry: sodium, potassium, glucose, blood urea nitrogen, creatinine, calcium, phosphate (inorganic), uric acid, total protein, albumin, bilirubin (total), alkaline phosphatase, aspartate transaminase, alanine transaminase, gamma-glutamyl transferase, lactate dehydrogenase
- Urinalysis: dipstick for presence of glucose, ketones and protein

8.2.2 Echocardiography

Doppler Flow Echocardiography is a noninvasive imaging modality for detecting pulmonary hypertension by evaluating the grade of tricuspid insufficiency, a condition potentially associated with Gaucher disease.

8.2.3 Anti-Taliglucerase alfa Antibodies

Anti-taliglucerase alfa antibodies, including neutralizing antibodies in subjects having a positive antibody response, will be assessed using a validated analytical method.

8.2.4 Adverse Events

8.2.4.1 Adverse Events (AE) and Serious Adverse Events (SAE)

An adverse event (AE) is any untoward medical occurrence in a subject participating in a clinical trial. An adverse event can be any unfavorable and unintended sign, symptom or disease temporally associated with the use of the study medication, whether or not considered related to the study medication. AEs will be collected from the start of treatment until 30 days following the final visit dose. Any events occurring prior to treatment will be recorded on the medical history page with the event name and onset date and end date if not continuing. Pre-existing, known clinically significant conditions observed at screening should be recorded as medical history.

This definition also includes accidental injuries, reasons for any change in medication (drug and/or dose) other than planned titration, reasons for admission to a hospital, or reasons for surgical procedures (unless for minor elective surgery for a pre-existing condition). It also includes adverse events commonly observed and adverse events anticipated based on the pharmacological effect of the study medication. Any laboratory abnormality assessed as clinically significant by the Investigator must be recorded as an adverse event.

A treatment emergent adverse event is any adverse event occurring after start of study medication and within the time of residual drug effect, or a pre-treatment adverse event or pre-existing medical condition that worsens in intensity after start of study medication and within the time of residual drug effect.

Adverse events should be recorded as diagnoses, if available. If not, separate sign(s) and symptom(s) are recorded. One diagnosis/symptom should be entered per record.

Note that death is not an event, but the cause of death is. An exception is the event of sudden death of unknown cause. Note that hospitalization is not an event; however, the reason for hospitalization is. Procedures are not events; the reasons for conducting the procedures are. In general, only the reason for conducting the procedure will be captured as an adverse event. However, if deemed necessary by the Investigator, a procedure can be captured along with the reason for conducting the procedure.

An overdose or medication error is not an adverse event unless it is temporally associated with an unfavourable or unintended sign or symptom.

Each AE is to be classified by the investigator as serious or non-serious. A serious adverse event (SAE) is any untoward medical occurrence or effect that occurs at any dose:

- Results in death
- Is life-threatening (i.e., an immediate risk of death)
- Requires in-patient hospitalization or prolongation of existing hospitalization
- Results in persistent or significant disability/incapacity
- Is associated with a congenital anomaly/birth defect
- Is an important medical event

An adverse event caused by an overdose or medication error is considered serious if a criterion listed in the definition above is fulfilled.

Important adverse events that may not result in death, may not be life-threatening, or do not require hospitalization may be considered serious when, based upon appropriate medical judgment, they may jeopardize the subject's safety or may require medical or surgical intervention to prevent one of the outcomes listed above.

Serious adverse events also include any other event that the investigator or sponsor judges to be serious or which is defined as serious by the regulatory agency.

The investigator is to report all directly observed adverse events and all adverse events spontaneously reported by the trial subject using concise medical terminology. In addition, each trial subject will be questioned about adverse events. The question asked will be "Since you began taking the study medication, have you had any health problems?"

8.2.4.2 Procedures for Assessing, Recording, and Reporting Adverse Events and Serious Adverse Events

Throughout the duration of the study, the Investigator will closely monitor each subject for evidence of drug intolerance and for the development of clinical or laboratory evidence of adverse events. All adverse events (expected or unexpected) which occur during the course of the study, whether observed by the Investigator or by the subject, and whether or not thought to be drug-related, will be reported and followed until resolution or until they become stable.

The description of the adverse event will include description of event, start date, stop date, intensity, if it was serious, relationship to test drug, change in test drug dosage, if the subject died, and if treatment was required.

Events will be coded to one of the following intensity categories below:

Severity	Definition
Mild	Awareness of signs or symptoms, but no disruption of usual activity
Moderate	Event sufficient to affect usual activity (disturbing)
Severe	Event causes inability to work or perform usual activities (unacceptable)

Events will be coded into one of the following causality categories as defined below:

Category	Definition
Unrelated	Clearly and incontrovertibly due only to extraneous causes, and does not meet criteria listed under possible or probable.
Unlikely	Does not follow a reasonable temporal sequence from administration. May have been produced by the subject's clinical state or by environmental factors or other therapies administered.
Possible	Follows a reasonable temporal sequence from administration, but may have been also produced by the subject's clinical state, environmental factors or other therapies administered.
Probable	Clear-cut temporal association with administration with improvement on cessation of investigational medicinal product or reduction in dose. Reappears upon rechallenge. Follows a known pattern of response to the investigational medicinal product.

Adverse events with the causality assessed as unrelated or unlikely are categorized as not related to study medication.

Adverse events with the causality assessed as possible or probable are categorized as related to study medication and are called adverse drug reactions.

All SAEs must be reported **immediately (no more than 24 hours after becoming aware of the event)**. The investigator must complete the eCRF SAE Report and contact the Safety Monitor (Dr Glen Park) IMMEDIATELY to 212-681-2100, according to the Serious Adverse Event Report Form Instructions.

9 STUDY PROCEDURES AND FLOW CHART

9.1 Study Flow Chart

Activity	Visit 0<br>Screening	Visit 1<br>Baseline	Visits 2-6	Visit 7<br>Month 3	Visits 8-13	Visit 14<br>Month 6	Visits 15-19	Visit 20<br>Month 9	Visits 21-27	Visit 28
	Day<br>-25±10	Day 1	Weeks 2,<br>4[1], 6, 8[1], 10	Week 12	Weeks 14, 16,<br>18, 20, 22, 24	Week 26	Weeks 28,<br>30, 32, 34, 36	Week 38	Weeks 40, 42,<br>44, 46, 48, 50	Week 52
Sign informed consent	X									
Demographics	X									
Medical history	X									
Current/Concomitant medications	X	X	X	X	X	X	X	X	X	X
Weight/height	X	X		X		X		X		X
Physical examination	X	X		X		X		X		X
Glucocerebrosidase activity	X									
Platelet count / hemoglobin (local lab)	X[2]	X	X	X		X		X		X
Haematology	X	X	X	X		X		X		X
Biochemistry	X	X	X	X		X		X		X
Serology (HIV, HBsAg, HCV)	X									
TSH, transferrin, B12 and folic acid	X									
Beta HCG (post-menarchal girls)	X									
Urinalysis	X	X	X	X		X		X		X
Review inclusion/exclusion criteria	X									
Anti-taliglucerase alfa antibodies		X		X		X		X		X
Organ volumes (MRI/ultrasound)		X								X
Tanner Stage		X				X				X
Echocardiography		X								X
Left Hand and wrist X-ray evaluation		X								X
Quality of Life (CHQ PF-28)		X				X				X
Chitotriosidase or CCL18 biomarker		X		X		X		X		X
Mutation analysis		X								
DEXA		X								X
IV Infusion (ERT)		X	X	X	X	X	X	X	X	X
Adverse Events		X	X	X	X	X	X	X	X	X

[1]Haemoglobin and platelets (local laboratory), haematology, biochemistry and urinalysis will be performed at Screening, Baseline, Weeks 4, 8 and 12 and then every 3 months.

[2]Include blood smear

9.2 Study Visits

9.2.1 Screening (Visit 0, Day -25 ± 10)

1. Obtain written informed consent from the subject and/or parent or gaurdian
2. Assign screening number
3. Demographics
4. Medical history
5. Physical examination, including weight and height
6. Current medications
7. Clinical laboratory tests
   - Blood platelet count including blood smear and hemoglobin (local)
   - Haematology
   - Biochemistry
   - Urinalysis
   - Thyroid stimulating hormone (TSH)
   - Transferrin
   - Vitamin B12
   - Folic Acid
   - Glucocerebrosidase activity
   - Blood pregnancy test (beta-HCG) for female subject of child-bearing potential
   - Serology for HIV, HBsAg, HCV
8. Review all inclusion and exclusion criteria and determine subject's eligibility
9. Request for subject approval and randomization

9.2.2 Visit 1 (Baseline, Day 1)

1. Assign to randomized treatment group
2. Concomitant medications
3. Physical examination including weight and height
4. MRI or ultrasound for spleen and liver volume
5. Echocardiography (for tricuspid insufficiency gradient)
6. Clinical laboratory tests
   - Blood platelet count and hemoglobin (local)
   - Haematology
   - Biochemistry
   - Urinalysis
   - Anti-taliglucerase alfa antibody
   - Biomarkers: chitotriosidase or CCL18
   - Mutation analysis
7. Left hand and wrist X ray for bone age
8. Tanner Stage
9. DEXA
10. Quality of Life (CHQ PF-28)
11. Taliglucerase alfa infusion

12. Adverse events

Perform the following procedures for the taliglucerase alfa dosing:

- Observe the subject clinically during and for a minimum of 1 hour after dosing.
- Evaluate vital signs every 15 minutes for the first hour and then every 30 minutes if the subject tolerates the infusion
- Evaluate the injection site
- A follow up telephone call with the subject will be held the day after the infusion

The subject should be reminded of the date of their next visit.

9.2.3 Visits 2 – 6 (Weeks 2, 4, 6, 8, 10 ± 3 Days)

Subjects will receive their taliglucerase alfa infusion at the selected medical center. Perform the following procedures for the taliglucerase alfa dosing:

- Observe the subject clinically for a minimum of 1 hour after dosing.
- Evaluate vital signs every 30 minutes if the subject has tolerated well previous infusions. Otherwise, evaluate vital signs every 15 minutes for the first hour.
- Evaluate the injection site.

Record adverse events and concomitant medications at each visit.

Laboratory tests [haemoglobin and platelets (local laboratory), haematology, biochemistry, urinalysis and anti-taliglucerase alfa antibody test] will be performed at visits 3 and 5.

Remind the subject of the date of their next visit.

9.2.4 Visit 7 (Month 3, Week 12 ± 7 Days)

Perform the following assessments:

1. Adverse events and concomitant medications
2. Physical examination including weight and height
3. Clinical laboratory tests
   - Blood platelet count and hemoglobin (local lab)
   - Haematology
   - Biochemistry
   - Biomarkers: chitotriosidase or CCL18
   - Anti-taliglucerase alfa antibody test
   - Urinalysis
4. Taliglucerase alfa infusion

Perform the following procedures for taliglucerase alfa dosing:

- Observe the subject clinically for 1 hour after dosing.
- Evaluate vital signs every 30 minutes
- Evaluate the injection site.

Subjects who tolerate the infusions well are eligible after this visit for transfer to home or infusion center treatment at the discretion of the investigator and Medical Director. The procedures to be followed will depend on local standards of care and procedures established at the center for administration of home care.

Remind the subject of the date of their next visit.

9.2.5 Visits 8 – 13 (Weeks 14, 16, 18, 20, 22, 24 ± 3 days)

Subjects will receive their taliglucerase alfa infusion at the selected medical center. Perform the following procedures for taliglucerase alfa dosing:

- Observe the subject clinically for 1 hour after dosing.
- Evaluate vital signs every 30 minutes
- Evaluate the injection site.

Record adverse events and concomitant medications at each visit.

Remind the subject of the date of their next visit.

9.2.6 Visit 14 (Month 6, Week 26 ± 7 days)

1. Adverse events and concomitant medications
2. Physical examination including body weight and height
3. Clinical laboratory tests
   - Blood platelet count and hemoglobin (local lab)
   - Haematology
   - Biochemistry
   - Biomarkers: chitotriosidase or CCL18
   - Anti human taliglucerase alfa antibody test
   - Urinalysis
4. Tanner Stage
5. Quality of Life
6. Taliglucerase alfa infusion

Perform the following procedures for taliglucerase alfa dosing:

- Observe the subjects clinically for 1 hour after dosing.
- Evaluate vital signs every 30 minutes
- Evaluate the injection site.

Remind the subject of the date of their next visit.

9.2.7 Visit 15 – 19 (Weeks 28, 30, 32, 34, 36 ± 3 days)

Subjects will receive their taliglucerase alfa infusion at the selected medical center. Perform the following procedures for taliglucerase alfa dosing:

- Observe the subjects clinically for 1 hour after dosing.
- Evaluate vital signs every 30 minutes
- Evaluate the injection site.

Record adverse events and concomitant medications at each visit.

Remind the subject of the date of their next visit.

9.2.8 Visit 20 (Month 9, Week 38 ± 7 days)

Perform the following assessments:

1. Adverse events and concomitant medications
2. Physical examination including weight and height.
3. Clinical laboratory tests
    - Blood platelet count and hemoglobin (local lab)
    - Haematology
    - Biochemistry
    - Urinalysis
    - Anti-taliglucerase alfa antibodies
    - Biomarkers: Chitotriosidase or CCL18
4. Taliglucerase alfa infusion

Perform the following procedures for taliglucerase alfa dosing:

- Observe the subject clinically for a minimum of 1 hour after dosing
- Evaluate vital signs every 30 minutes
- Evaluate the injection site.

Remind the subject of the date of their next visit.

9.2.9 Visit 21 – 27 (Weeks 40, 42, 44, 46, 48, 50 ± 3 days)

Subjects will receive their taliglucerase alfa infusion at the selected medical center. Perform the following procedures for taliglucerase alfa dosing:

- Observe the subject clinically for 1 hour after dosing.
- Evaluate vital signs every 30 minutes
- Evaluate the injection site.

Record adverse events and concomitant medications at each visit.

Remind the subject of the date of their next visit.

9.2.10 Visit 28 (Month 12, Week 52 ± 7 days)

Perform the following assessments:

1. Adverse events and concomitant medications
2. Physical examination including weight and height.
3. Clinical laboratory tests
   - Blood platelet count and hemoglobin (local lab)
   - Haematology
   - Biochemistry
   - Urinalysis
   - Anti-taliglucerase alfa antibodies
   - Biomarkers: Chitotriosidase or CCL18
4. Quality of life
5. Echocardiography
6. Spleen and liver volume by MRI or ultrasound
7. Tanner Staging
8. Left hand and wrist X-ray for bone age
9. DEXA
10. Taliglucerase alfa infusion

Perform the following procedures for taliglucerase alfa dosing:

- Observe the subject clinically for a minimum of 1 hour after dosing
- Evaluate vital signs every 30 minutes
- Evaluate the injection site.

10 STATISTICAL METHODS PLANNED AND SAMPLE SIZE

11 QUALITY CONTROL AND QUALITY ASSURANCE

11.1 Source Data and Records

Source data are all the information in original records and certified copies of original records of clinical findings, observations, laboratory reports, data sheets provided by the sponsor or other activities in the study, which are necessary for the reconstruction and evaluation of the study. The investigator will permit study-related monitoring, audit(s), IRB review(s) and regulatory inspection(s), with direct access to all the required source records.

All study records will be retained for a period of time as defined by the regulatory authority for the country in which the investigation is conducted. Generally, this means at least 2 years following the date on which the drug is approved by the regulatory authority for marketing for the purposes that were the subject of the investigation. In other situations (e.g., where the investigation is not in support of or as part of an application for a research or marketing permit), a period of 2 years following the date on which the entire clinical program is completed, terminated or discontinued or the investigational application under which the investigation is being conducted is terminated or withdrawn by the regulatory authorities.

In the event the Investigator retires, relocates or for any other reason withdraws from the responsibility for maintaining records for the period of time required, custody of the records may be transferred to any other person who will accept responsibility for the records. Notice of such a transfer must be given in writing to the Sponsor. The Investigator must contact the Sponsor prior to disposal of any records related to this study.

11.2 Reporting of Results

The Case Report Form (CRF) is an integral part of the study and subsequent reports. The CRF must be used to capture all study data recorded in the subject's medical record. The CRF must be kept current to reflect subject status during the course of the study. Only a subject screening and randomization number and subject initials will be used to identify the subject.

The monitor is responsible for performing on-site monitoring at regular intervals throughout the study to verify adherence to the protocol; verify adherence to local regulations on the conduct of clinical research; and ensure completeness, accuracy, and consistency of the data entered in the CRF.

Protalix Ltd. or their designee will monitor completed Case Report Forms (CRFs). A case report form will be provided for each screened subject.

All protocol-required information collected during the study must be entered by the Investigator, or designated representative, in the Target e*CRF™, an Internet-based electronic data collection system. All details of the CRF completion and correction will be explained to the investigator. The management module of Target e*CRF™ includes edit check and query systems that seamlessly integrate with the data entry system. All modifications to the data in the eCRF are tracked by an electronic audit trail (date and identity of the person making the change are instantaneously recorded). Target e*CRF™ is 21CFR Part 11 compliant.

If the Investigator authorizes other persons to make entries in the CRF, the names, positions, and signatures of these persons must be supplied to the sponsor.

The Investigator, or designated representative, should complete the eCRF as soon as possible after information is collected, preferably on the same day that a study subject is seen for an examination, treatment, or any other study procedure. Any outstanding entries must be completed immediately after the final examination. By design, an explanation must be provided for all missing data, altered data, and/or out of range data.

The completed case report form must be reviewed and signed by the Investigator named in the study protocol or by a designated sub investigator.

Final monitored and audited eCRFs will be provided by the Sponsor to the sites at the end of the study in the format of a PDF file.

11.3 Confidentiality of Subject Data

The investigator will ensure that the confidentiality of the subjects' data will be preserved. In the CRF or any other documents submitted to the sponsor, the subjects will not be identified by their names, but by an identification system, which consists of their initials and number in the study. The investigator will maintain documents not meant for submission to the sponsor, e.g., the confidential subject identification code and the signed informed consent forms, in strict confidence.

12 REPORTING AND PUBLICATION

12.1 Confidentiality of Study Data

Any information relating to the study product or the study, including any data and results from the study, will be the exclusive property of the sponsor. The investigator and any other persons involved in the study will protect the confidentiality of this proprietary information belonging to Protalix Ltd..

12.2 Publication Policy

Protalix Ltd. agrees to make the report of the multicenter study results available to investigators for preparing a publication of the results in meeting abstract or medical journal form. Protalix Ltd. will have 30 days to review any proposed publication of the data for accuracy and proprietary information.

13 Appendices

13.1 Appendix 1. Vial Label

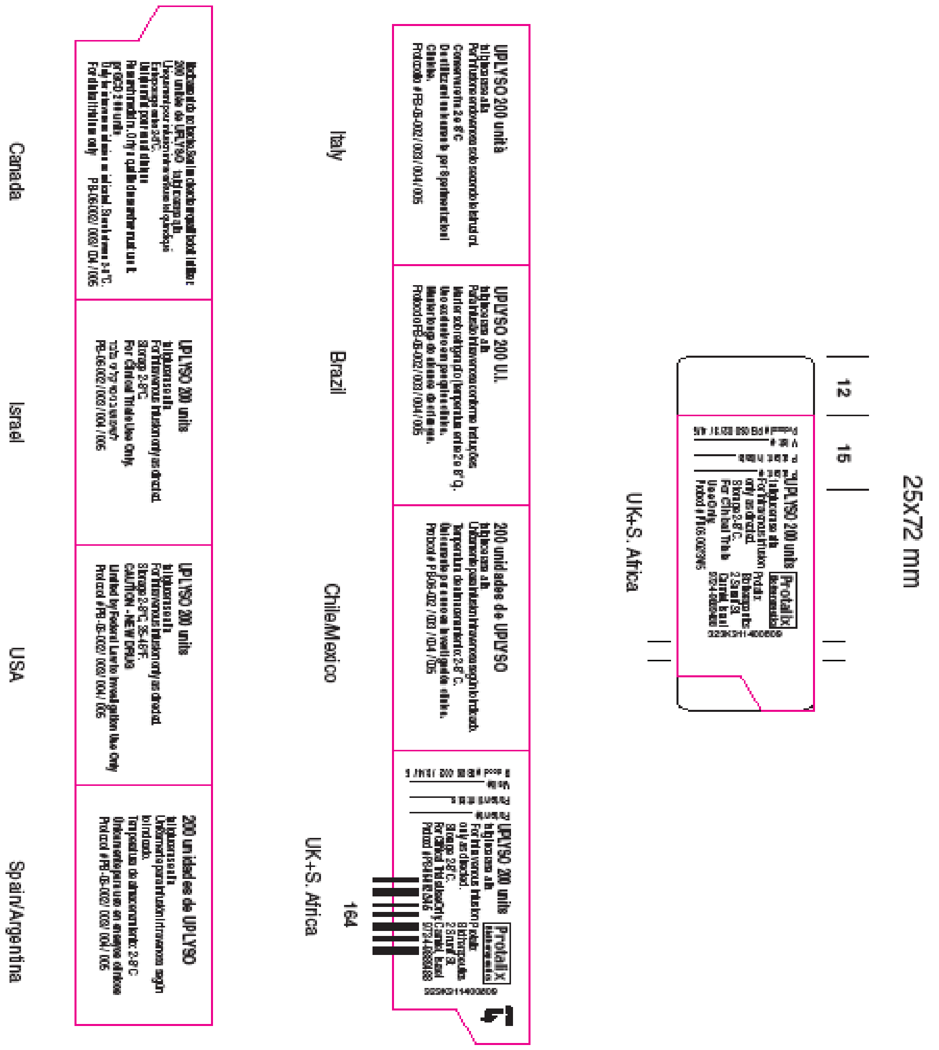

13.2 Appendix 2. Infusion Rate Algorithm

The infusion rate (IR) may be adjusted according to individual subject symptoms and signs. The assumptions with respect to adverse experiences to the infusion are:

1. Most of the subjects will tolerate the infusion without any special symptom or event. The infusion rate may be increased over time to the maximal rate 2.25 ml/minor after discussion with the Medical Monitor (an infusion duration of 1 hour).
2. Subjects presenting symptoms and signs of **severe** hypersensitivity will be evaluated according to the WHO Drug Toxicity criteria and there may be a discontinuation of treatment according to the protocol.
3. Subjects may present signs and symptoms that will respond to reducing of the infusion rate and may not appear at the next infusion.
4. Tolerability and the subject specific infusion rate will be assessed and decided by the Investigator according to vital signs and clinical status of the subject.

Definitions to be applied regarding tolerability of infusions are as follows:

**Good tolerability**	**Partial tolerability**	**Poor tolerability**
Infusion was performed without Signs and symptoms (such as burning, pruritus, flushing, discomfort, or change in vital signs).	Signs and symptoms appeared during the infusion **and resolved after slowing infusion rate** or at the end of the infusion.	Signs and symptoms meeting the definitions of WHO Grade 1 or 2 toxicity responding to reduction of infusion rate or responding to treatment (example, antihistamine for urticaria).

The specific algorithm to be followed is below.

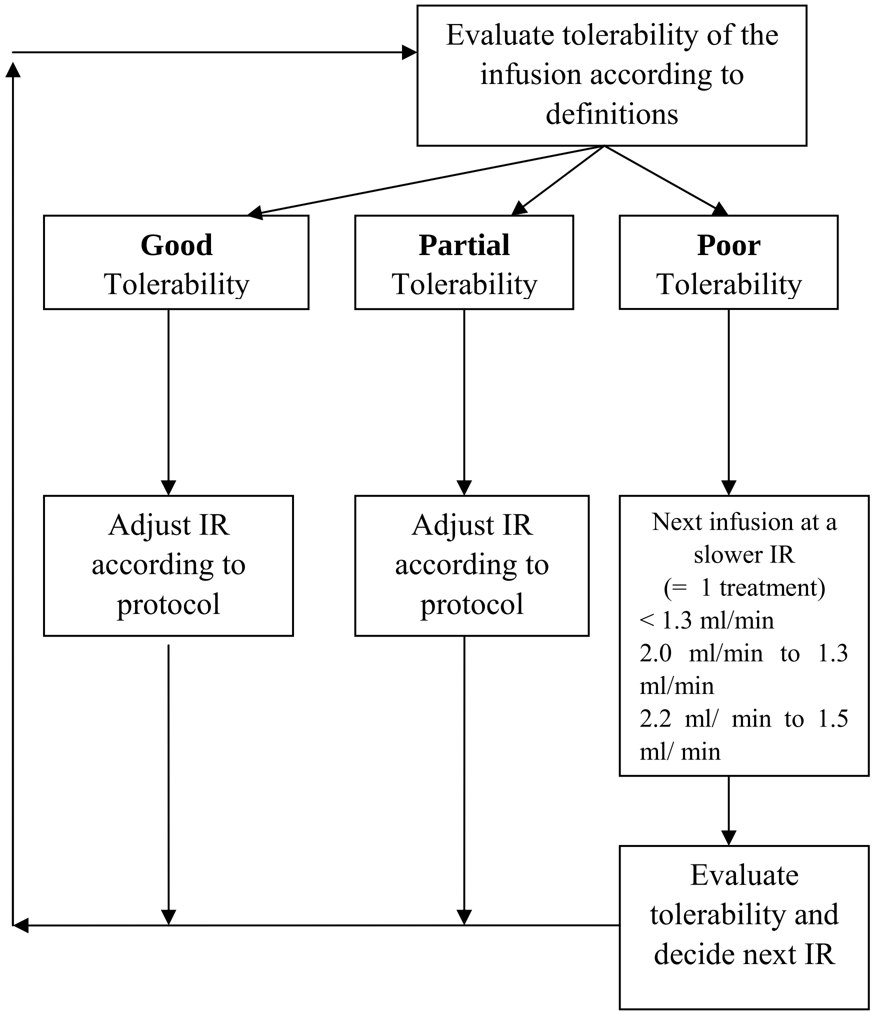
Evaluate tolerability of the infusion according to definitions
Good Tolerability
Partial Tolerability
Poor Tolerability
Adjust IR according to protocol
Adjust IR according to protocol
Next infusion at a slower IR (= 1 treatment) < 1.3 ml/min 2.0 ml/min to 1.3 ml/min 2.2 ml/ min to 1.5 ml/ min
Evaluate tolerability and decide next IR

13.3 Appendix 3. Taliglucerase alfa Hypersensitivity Evaluation and Treatment Algorithm

During and after infusion of taliglucerase alfa, the following algorithm will be followed to monitor and manage the occurrence of hypersensitivity, anaphylaxis, or anaphylactoid reactions.

Clinical signs

Early

- Sensation of warmth and itching
- Feelings of anxiety or panic

Moderate

- Pruritus
- Flushing
- Urticaria
- Chest discomfort
- Mild Hypotension

Progressive

- Erythematous or massive urticarial rash
- Edema of face, neck, soft tissues

Severe

- Hypotension
- Bronchospasm (wheezing)
- Laryngeal edema (dyspnea, stridor, aphonia, drooling)
- Arrhythmias

Treatment algorithm

With the onset of any of the above clinical signs, immediately discontinue study medication administration and initiate the following monitoring.

- Continuous electrocardiographic monitoring
- Continuous pulse oximetry
- Measure blood pressure every 5 minutes
- Perform chest auscultation every 5 minutes

- Blood samples need to be collected for Tryptase (29-33) and antibodies. Tryptase samples need to be withdrawn at:

    - 1st sample taken 0.25-3 hours after onset of symptoms
    - 2nd sample taken between 3-6 hours
    - 3rd sample taken 24-48 hours to verify the return to baseline.

In the case of progressive or severe hypersensitivity, treat appropriately and withdraw the subject from the study.

Treat as follows:

Urticaria or edema of the face, neck, or soft tissues

- Epinephrine 1:1000 solution, 0.5 mL subcutaneously, repeat as needed every 5-10 minutes
- Antihistamines
- Corticosteroids

Hypotension (systolic blood pressure (SBP) ≤ 90 mmHg)

- Isotonic sodium chloride solution, 1 L every 30 minutes as needed to maintain SBP > 90 mmHg
- Epinephrine 1:10,000 solution given IV at 1 µg/minute initially, then 2-10 µg/minute to maintain SBP > 90 mmHg
- Norepinephrine 4 mg in 1 L 5% dextrose in water given IV at 2-12 µg/min to maintain SBP > 90 mmHg
- Glucagon 1 mg in 1 L 5% dextrose in water give IV at 5-15 µg/minute for refractory hypotension

Bronchospasm

- Oxygen by face mask at 6-8 L/minute to maintain oxygen saturation at > 90%
- Epinephrine 1:1000 solution, 0.5 mL subcutaneously
- Albuterol 0.5 mL of 0.5% solution in 2.5 mL of sterile saline every 15 minutes up to three doses
- Inhaled beta-agonists
- Corticosteroids

Laryngeal edema

- Epinephrine 1:1000 solution, 0.5 mL subcutaneously, repeat as needed every 5 to 10 minutes
- Corticosteroids

Premedication

Premedication for subsequent taliglucerase alfa infusions may be considered at the discretion of the investigator and Medical Director for subjects experiencing early clinical signs of hypersensitivity or rash/urticaria that responds promptly to oral antihistamine administration (see also Appendix 2 for adjustment of infusion rate). The premedication will be applied according to the following steps as needed to prevent progressive hypersensitivity:

1. Antihistamine (H1 blocker: diphenhydramine, hydroxyzine, cetrizine, loratadine, desloratidine) at a standard dose 12 hours and 2 hours before the start of the infusion.

2. H1 blocker plus H2 blocker (ranitidine, cimetidine, famotidine) at standard doses 12 hours and 2 hours before the start of the infusion.

3. H1 blocker plus H2 blocker plus prednisone up to 50 mg administered 12 hours and 2 hours before the start of the infusion.

13.4 Appendix 4. MRI

13.4.1 Subjects and sites

Approximately 10 subjects will be enrolled in this trial by 5 to 10 sites world wide.

13.4.2 Magnetic Resonance Imaging (MRI) data

Each subject enrolled in this trial will have 2 MRI timepoints during the course of the trial at Baseline and Month 12.

For each timepoint, the same set of T1 and T2-weighted MRI sequences will be acquired. These sequences will be defined based on their equipment and abilities to provide sufficient image quality and contrast for organ (spleen and liver) detection and quantification, and also full spleen and liver coverage in Gaucher subjects. No contrast agent will be used. If baseline MRI cannot be obtained, organ volumes can be obtained by ultrasound technology with approval of the Medical Director.

13.4.3 MRI evaluation parameters

The following MRI parameters will be evaluated during this trial:

- Volume of spleen (in cm^3)
- Volume of liver (in cm^3)

13.4.4 Sites and image data management

All image management activities will be centralized and conducted by an independent imaging Contract Research Organization (imaging CRO) with operational capabilities in Europe and the United States in compliance with all regulatory requirements. An overview of the main activities performed by the imaging CRO is provided in the next sections.

13.4.4.1 Standardization of image acquisition, initial site qualification

The image acquisition procedure will be standardized by the imaging CRO among all participating sites. The same image acquisition and management procedure will be used by all sites. This procedure will be defined by the imaging CRO and approved by the Sponsor. The sites will be trained and qualified by the imaging CRO prior to start of subject enrolment. Each site will provide test MRI scan(s) during the initial site qualification phase. The source of the test scan(s) will be (in order of preference) a subject volunteer, a healthy volunteer, or the screening image from the first subject tested at the site. All images will be anonymized by the sites (in order to remove any subject-related nominative information) and provided in digital format (DICOM). Only digital images will be centrally processed by the imaging CRO.

13.4.4.2 Subject Sedation

Pediatric subjects may require sedation in order to obtain the high quality images required. Sites may use standard sedation protocols approved by the institution.

13.4.4.3 Quality control of image data and site Quality Assurance during the course of the trial

The image data will be collected and quality controlled by the imaging CRO for checking the technical adequacy, the compliance of data acquisition with the study imaging protocol, the anonymization of the images and the diagnostic quality of the images (their appropriateness for centralized evaluations). If any quality-related issue is detected by the imaging CRO, specific queries will be sent to the sites to implement appropriate corrective (such as potential repeat scans whenever possible) and preventive actions.

13.4.5 Image processing and centralized analysis

13.4.5.1 Spleen and liver segmentation

In order to improve the quality of centralized image evaluations, the anatomical structures of interest (liver and spleen) will be pre-detected using automated three-dimensional (3D) segmentation software. 3D segmentation consists in classifying image voxels in multiple tissue classes based on the MRI signal intensities and also the piecewise contiguity of image voxels representing given organs (liver and spleen in this case). The resulting contours representing the liver and the spleen will be manually edited and quality controlled by an expert technician at the imaging CRO. The resulting liver and spleen contours will be submitted for final approval to independent and blinded readers.

13.4.5.2 Centralized Image Review by Independent Readers

The MRI data will be centrally evaluated in a fully blinded manner by an independent reader. The reading sessions will be organized at the imaging CRO site. The same image evaluation procedure will be used for all subjects' MRI scans in this trial.

Expertise of independent readers, training sessions

The reader will be a Senior Radiologists with a significant experience in liver and spleen imaging and MRI. The reader will provide a detailed, signed and dated CV including scientific references. The imaging CRO will subcontract with the reader for the payment of honoraria.

The reader will be trained prior to start of centralized image review sessions. S/he will be provided with a Reader's Kit including an Image Evaluation Software User Guide. Test cases representing non-study Gaucher subjects (provided by the Sponsor) will be used as well as

potential additional MRI scans from other indications for the training of the readers. Intra- and inter-reader reproducibility rates will be estimated for the quantification of liver and spleen volumes. The potential causes of variability and the main consensus issues (delineation of liver and spleen, specific issues related to Gaucher subjects, technical issues such as partial volume effect, potential movements of organs and image artifacts, etc.) will be discussed with the readers and documented.

Conduct of centralized image review sessions

The reader will be fully blinded with regard to treatment groups, subject's ID and site number. Each MRI timepoint will be evaluated by for the quantification of the liver and spleen volumes. All timepoints will be fully randomized and displayed separately. All evaluations will be performed after collection of Baseline and month 12 timepoint of a pre-defined number of subjects before starting the review sessions. In order to leave the reader blinded with respect to the time sequence, the reader will not be aware of the order of the MRI timepoints which will be fully randomized by the imaging CRO.

All evaluation results including spleen and liver contours, and the imaging CRO electronic Case Report Forms (eCRFs) will be saved in the trial database. In compliance with regulatory requirements (including FDA's 21 CFR Part 11), audit trails will be generated for all image manipulation and evaluation steps and the readers will use an electronic signature system to authenticate themselves for the evaluation of each MRI timepoint. The trial specific Image Review Software will be developed, validated and documented by the imaging CRO in compliance with regulatory requirements.

13.4.6 Data and report transfers to Sponsor

- Review sessions will be exported to the Sponsor using a pre-defined, standardized and secure data transfer procedure.
- Subject and MRI data tracking information (study progress reports) will be communicated to the Sponsor using a pre-defined, standardized and secure data transfer procedure.
- The final Study database will be submitted to the Sponsor in digital format.
- Spleen and liver volumes will be directly computed from organ masks validated by the blinded reader. The result of this quantification will be stored in the database. The file containing final results will be automatically generated from the values of the database, thus avoiding any result to be modified after the review sessions.

13.4.7 Direct access to Study data

- A Direct access to Study data will be made possible by the imaging CRO for audit purposes.
- Such Study data include:

- Information related to interactions between the imaging CRO and the sites (Queries, Data Clarification Forms, test data submitted by the sites, etc.)
- Native MRI data
- Data processed and generated by the imaging CRO
- Data generated by the reader (including image masks of liver and spleen and eCRFs)
- Audit trails

13.4.8 Unevaluable MRI:

Unevaluable MRI data can result from a poor quality image, due to subject motion, improper organ coverage, technical problems with the image transmission to the imaging CRO, etc. The imaging CRO procedures for ensuring quality images are meant to reduce or eliminate such poor quality images.

If an adequate subject image cannot be obtained for a given time point in the study, the problem with the image will be documented at the imaging CRO and in the e*CRF™. In addition, the imaging CRO will document all attempted corrective actions with the investigative site imaging centre.

13.5 Appendix 5: WHO Common Toxicity Criteria

Category	Toxicity	Grade0	Grade1	Grade2	Grade3	Grade4
Haematology	WBC (x103/l)	4	3.0 - 3.9	2.0 - 2.9	1.0 - 1.9	< 1.0
Haematology	Platelets (x103/l)	WNL	75.0 - normal	50.0 - 74.9	25.0 - 49.9	< 25.0
Haematology	Haemoglobin (g/dl)	WNL	10.0 - normal	8.0 - 9.9	6.5 - 7.9	< 6.5
Haematology	Granulocytes/ Bands (x103/l)	2	1.5 - 1.9	1.0 - 1.4	0.5 - 0.9	< 0.5
Haematology	Lymphocytes (x103/l)	2	1.5 - 1.9	1.0 - 1.4	0.5 - 0.9	< 0.5
Haematology	Haemorrhage	none	mild, no	gross, 1 - 2 units transfusion per episode	gross, 3 - 4 units transfusion per episode	massive, > 4 units transfusion per episode
Coagulation	Fibrinogen	WNL	0.99 - 0.75 x N	0.74 - 0.50 x N	0.49 - 0.25 x N	< 0.25 x N
Coagulation	Prothrombin time(Quick)	WNL	1.01 - 1.25 x N	1.26 - 1.50 x N	1.51 - 2.00 x N	> 2.00 x N
Coagulation	Partial thrombo-plastin time	WNL	1.01 - 1.66 x N	1.67 - 2.33 x N	2.34 - 3.00 x N	> 3.00 x N
Metabolic	Hyperglycaemia (mg/dl)	< 116	116 - 160	161 - 250	251 - 500	> 500 or ketoacidosis
Metabolic	Hypoglycaemia (mg/dl)	> 64	55 – 64	40 - 54	30 - 39	< 30
Metabolic	Amylase	WNL	< 1.5 x N	1.5 - 2.0 x N	2.1 - 5.0 N	> 5.0 x N
Metabolic	Hypercalcaemia (mg/dl)	< 10.6	10.6 - 11.5	11.6 - 12.5	12.6 - 13.4	13.5
Metabolic	Hypocalcaemia (mg/dl)	> 8.4	8.4 - 7.8	7.7 - 7.0	6.9 - 6.1	6
Metabolic	Hypomagnesaemia (mg/dl)	> 1.4	1.4 - 1.2	1.1 - 0.9	0.8 - 0.6	0.5

**Category**	**Toxicity**	**Grade0**	**Grade1**	**Grade2**	**Grade3**	**Grade4**
Gastrointestinal	Nausea	none	able to eat reasonable intake	intake significantly decreased but can eat	no significant intake	—
Gastrointestinal	Vomiting	none	1 episode in 24 hrs	2 - 5 episodes in 24 hrs	6 - 10 episodes in 24 hrs	> 10 episodes in 24 hrs or requiring paren- teral support
Gastrointestinal	Diarrhoea	none	increase of 2 - 3 stools / day over pre-Rx	increase of 4 - 6 stools / day, or nocturnal stools, or moderate cramping	increase of 7 - 9 stools / day, or incontinence, or severe cramping	increase of > 10 stools / day or grossly bloody diarrhoea, or need for paren- teral support
Gastrointestinal	Stomatitis	none	painless ulcers, erythema, or mild soreness	painful erythema, oedema, or ulcers but can eat solids	painful erythema, oedema, or ulcers and cannot eat solids	requires parenteral or enteral support for alimentation
Liver	Bilirubin (N = 17 µmol/L)	WNL	-----	< 1.5 x N	1.5 - 3.0 x N	> 3.0 x N
Liver	Transaminase (SGOT, SGPT)	WNL	2.5 x N	2.6 - 5.0 x N	5.1 - 20.0 x N	> 20.0 x N
Liver	Alk Phos or 5 nucleotidase	WNL	< 2.5 x N	2.6 - 5.0 x N	5.1 - 20.0 x N	> 20.0 x N
Liver	Liver- clinical	No change from baseline	-----	-----	precoma	hepatic coma
Kidney, bladder	Creatinine	WNL	< 1.5 x N	1.5 - 3.0 x N	3.1 - 6.0 x N	> 6.0 x N
Kidney, bladder	Proteinuria	No change	1 (+) or < 0.3 g% or 3 g/L	2 - 3 (+) or 0.3 - 1.0 g% or 3 - 10 g/L	4 (+) or > 1.0 g% or > 10g/L	nephrotic syndrome
Kidney, bladder	Haematuria	Negative	microscopic only	gross, no clots no Rx needed	gross and clots bladder irrigation	requires trans- fusion or cystectomy
Kidney, bladder	Weight gain/ loss	< 5.0 %	5.0 - 9.9 %	10.0 - 19.9 %	20.00%	-----
Pulmonary	Pulmonary	none or no change	asymptomatic, with abnormal- ity in PFTs	dyspnoea on significant exertion	dyspnoea at normal level of activity	dyspnoea at rest

**Category**	**Toxicity**	**Grade0**	**Grade1**	**Grade2**	**Grade3**	**Grade4**
Cardiac	Cardiac arrhythmias	none	asymptomatic, transient, requiring no therapy	recurrent or persistent, no therapy required	requires treatment	requires monitoring; or hypotension, or ventricular tachycardia or fibrillation
Cardiac	Cardiac function	none	asymptomatic, decline of resting ejection fraction by less than 20 % of baseline value	asymptomatic, decline of resting ejection fraction by more than 20 % of baseline value	mild CHF, responsive to therapy	severe of refractory CHF
Cardiac	Cardiac ischaemia	none	non-specific T- wave flattening	asymptomatic, ST and T wave changes suggesting ischaemia	angina without evidence of infraction	acute myocardial infarction
Cardiac	Cardiac- pericardial	none	asymptomatic effusion, no intervention required	pericarditis (rub, chest pain, ECG changes)	symptomatic effusion; drainage required	tamponade; drainage urgently required
Cardiac	Hypertension	none or no change	asymptomatic, transient increase by greater than 20 mm Hg (D) or to > 150 / 100 if previously WNL. No treatment required.	recurrent or persistent increase by greater than 20 mm HG (D) or to > 150 / 100 if previously WNL. No treatment required.	requires therapy	hypertensive crisis
Cardiac	Hypotension	none or no change	changes requiring no therapy (including transient orthostatic hypotension)	requires fluid replacement or other therapy but not hospitalisation	requires therapy and hospitalisation; resolves within 48 hours of stopping the agent	requires therapy and hospitalisation for > 48 hrs after stopping the agent
Neurologic	Neuro: sensory	none or no change	mild paraesthesias; loss of deep tendon reflexes	mild or moderate objective sensory loss moderate paraesthesias	severe objective sensory loss or paraesthesias that interfere with function	-----
Neurologic	Neuro: motor	none or no change	subjective weakness; no objective findings	mild objective weakness without significant impairment of function	objective weakness with impairment of function	paralysis

Category	Toxicity	Grade0	Grade1	Grade2	Grade3	Grade4
Neurologic	Neuro: cortical	none	mild somnolence or agitation	moderate somnolence or agitation	severe somnolence, (>50 % waking hours), agitation, confusion, disorientation or hallucinations	coma, seizures, toxic psychosis
Neurologic	Neuro: cerebellar	none	slight incoordination, dysdiadochokinesia	intention tremor, dysmetria, slurred speech, nystagmus	locomotor ataxia	cerebellar necrosis
Neurologic	Neuro: mood	no change	mild anxiety or depression	moderate anxiety or depression	severe anxiety or depression	suicidal ideation
Neurologic	Neuro: headache	none	mild	moderate or severe but transient	unrelenting and severe	-----
Neurologic	Neuro: constipation	none or no change	mild	moderate	severe	ileus > 96 hrs
Neurologic	Neuro: hearing	none or no change	asymptomatic, hearing loss on audiometry only	tinnitus	hearing loss interfering with function but correctable with hearing aid	deafness not correctable
Neurologic	Neuro: vision	none or no change	-----	-----	symptomatic subtotal loss of vision	blindness
Pain	Pain	none	mild	moderate	severe	requires narcotics
Skin	Skin	none or no change	scattered macular ot papular eruption or erythema that is asymptomatic	scattered macular or papular eruption or erythema with pruritus or other associated symptoms	generalised symptomatic macular, papular or vesicular eruption	exfoliative dermatitis or ulcerating dermatitis
Alopecia	Alopecia	no loss	mild hair loss	pronounced or total hair loss	-----	-----

Category	Toxicity	Grade0	Grade1	Grade2	Grade3	Grade4
Allergy	Allergy	none	transient rash, drug fever < 38°C (100.4°F)	urticaria, drug fever 38°C (100.4°F), mild bronchospasm	serum sickness, bronchospasm requiring parenteral medication	anaphylaxis
Local	Local	none	pain	pain and swelling with inflammation or phlebitis	ulceration	plastic surgery indicated
Fever of unknown origin	Fever of unknown origin	none	37.1 - 38.0° C 98.7° - 100.4° F	38.1 - 40.0°C 100.5 - 104°F	> 40.0°C > 104.0°F for less than 24hrs	> 40.0°C (>104°F) for more than 24 hrs or accompanied by hypotension
Infection	Infection	none	mild	moderate	severe	life-threatening
Additional events	Asthenia	Analogous to Karnofsky index (WHO grading)				
Additional events	Chills	Analogous to fever				
Additional events	Peripheral oedema	analogous to weight gain				
Additional events	Anorexia	analogous to weight loss				
