# Supplementary material for: Immunogenicity of glycans on biotherapeutic drugs produced in plant expression systems—The taliglucerase alfa story
Source: PLoS One. 2017 Oct 31;12(10):e0186211. doi: 10.1371/journal.pone.0186211 (PMC5663370; doi:10.1371/journal.pone.0186211)
Supplement: S4 File — (PDF) [file pone.0186211.s004.pdf]

	Patient #	Binding ADA		Anti-plant glycans ADA	
		Anti taliglucerase alfa IgG results	Highest titer	Anti-plant glycan results	% Immunodepletion
ERT naive adults (PB-06-001)	01-001	Positive at Visits 7,16,18, 20	8094 at Visit 20	Negative	NA
	01-002	Positive at visit 11 and end of study (Discontinued after Visit 12)	1097 at Visit 11	Negative	NA
	01-003	Positive at Visit 2	96 at Visit 2	Negative	NA
	01-004	Positive at Visits 2, 3, 9, 11, 18, 20	1119 at Visit 18	Positive at Visits 2, 3, 9, 11	45.4-51.9%
	01-005	Positive at Visits 9, 11, 13, 14, 16, 18, 20	1117 at Visit 11	Negative	NA
	01-006	Positive at Visit 7 only	100 at Visit 7	Negative	NA
	01-007	Positive at Visits 3, 4,5,7, 13, 14, 16,18, 20	1467 at Visit 14	Positive at visit 3 only	37.50%
	01-008	Positive at Visit 13	84 at Visit 13	Negative	NA
	01-009	Positive at Visits 13, 14, 16, 18	452 at Visit 13	Negative	NA
	01-010	Positive at Visits 14, 20	ND	Negative	NA
	01-011	Positive at Visits 13, 14, 16, 20	1597 at Visit 16	Negative	NA
	01-012	Positive at Visits 13, 14, 18, 20	6355 at Visit 16	Positive at visit 35 only	31.60%
	01-013	Positive at Visits 13, 16	695 at Visit 13	Negative	NA
	01-014	Positive at all Visits	184 at Visit 18	Positive at all IgG^+ Visits	66.67-84.00%
	01-015	Positive at Visit 4,5	339 at Visit 4	Negative	NA
	01-016	Positive at Visits 16, 20	525 at Visit 20	Negative	NA
	01-017	Positive at Visit 1	NA	Negative	NA
	01-018	Positive at Visits 9,11,16	6999 at Visit 9	Negative	NA
	01-019	Negative	NA	Negative	NA
	01-020	Negative	NA	Negative	NA
	01-021	Negative	NA	Negative	NA
	01-022	Negative	NA	Negative	NA
	01-023	Negative	NA	Negative	NA
	01-024	Negative	NA	Negative	NA
	01-025	Positive at Visit 3 only	ND	Negative	NA
	01-026	Negative	NA	Negative	NA
	01-027	Negative	NA	Negative	NA
	01-028	Negative	NA	Negative	NA
	01-029	Negative	NA	Negative	NA
	01-030	Negative	NA	Negative	NA
	01-031	Negative	NA	Negative	NA
	01-032	Negative	NA	Negative	NA
ERT experienced adults (PB-06-002)	02-001	Positive sample at Visits 1, 3, 14, 17, 20	3542 at Visit 14	Negative	NA
	02-002	Positive sample at Visits 10, 14, 17, 20	23045 at Visit 20	Negative	NA
	02-003	Positive sample at Visits 14, 17, 20	2768 at Visit 17	Negative	NA
	02-004	Negative	NA	Negative	NA
	02-005	Negative	NA	Negative	NA
	02-006	Negative	NA	Negative	NA
	02-007	Negative	NA	Negative	NA
	02-008	Negative	NA	Negative	NA
	02-009	Negative	NA	Negative	NA
	02-010	Negative	NA	Negative	NA
	02-011	Negative	NA	Negative	NA
	02-012	Negative	NA	Negative	NA
	02-013	Negative	NA	Negative	NA
	02-014	Negative	NA	Negative	NA
	02-015	Negative	NA	Negative	NA
	02-016	Negative	NA	Negative	NA
	02-017	Negative	NA	Negative	NA
	02-018	Negative	NA	Negative	NA
	02-019	Positive sample at Visit 5 only	ND	Negative	NA
	02-020	Negative	NA	Negative	NA
	02-021	Negative	NA	Negative	NA
	02-022	Positive sample at Screen and Visit 1	978 at Visit 1	Positive predosing	58.58%, 61.13%
	02-023	Negative	NA	Negative	NA
	02-024	Negative	NA	Negative	NA
	02-025	Negative	NA	Negative	NA
	02-026	Negative	NA	Negative	NA
ERT experienced pediatric (PB-06-002)	02-027	Negative	NA	Negative	NA
	02-026	Positive at Visits 1, 3, 5	145 at Visit 1	Positive at Visits 1, 3, 5	71.02-87.17%
	02-027	Negative	NA	Negative	NA
	02-026	Positive at Visits 1, 3	67 at Visit 1	Positive at Visits 1, 3	27.20%, 31.10%
	02-027	Negative	NA	Negative	NA
ERT naive pediatric (PB-02-005)	05-001	Positive at Visit 7 only	189 at Visit 7	Positive at visits 7 only	74.86%
	05-002	Positive at Visits 14, 20, 27	524 at Visit 20	Negative	NA
	05-003	Positive at Visits 1, 3, 7	233 at Visit 5	Positive at Visits 1, 3, 7	61.96-73.57%
	05-004	Negative	NA	Negative	NA
	05-005	Negative	NA	Negative	NA
	05-006	Negative	NA	Negative	NA
	05-007	Negative	NA	Negative	NA
	05-008	Negative	NA	Negative	NA
	05-009	Negative	NA	Negative	NA
	05-010	Negative	NA	Negative	NA
	05-011	Negative	NA	Negative	NA

NA Not Applicable
ND Not Determined
